# Associations between trajectories of plasma biomarkers for Alzheimer’s disease, brain structures, and cognitive function: a prospective cohort study in the UK Biobank

**DOI:** 10.1038/s41380-025-03166-y

**Published:** 2025-08-28

**Authors:** Xin Huang, Xiaolei Han, Hongli Chang, Tianyu Yu, Yi Dong, Ming Mao, Jiahao Ding, Xuewei Li, Huanjun Liu, Minle Tian, Xiaomeng Li, Yumei Wang, Yanping Bao, Yifeng Du, Chengxuan Qiu, Lin Lu, Yongxiang Wang

**Affiliations:** 1 Key Laboratory of Endocrine Glucose & Lipids Metabolism and Brain Aging, Ministry of Education, Department of Neurology, Shandong Provincial Hospital affiliated to Shandong First Medical University, Jinan, Shandong P.R. China; 2 Shandong Institute of Brain Science and Brain-inspired Research; Medical Science and Technology Innovation Center, Shandong First Medical University & Shandong Academy of Medical Sciences, Jinan, Shandong P.R. China; 3 Department of Neurology, Shandong Provincial Hospital affiliated to Shandong University, Jinan, Shandong P.R. China; 4 Department of Clinical Psychology, Shandong Provincial Hospital affiliated to Shandong First Medical University, Jinan, Shandong P.R. China; 5 National Institute on Drug Dependence and Beijing Key Laboratory of Drug Dependence Research, Peking University, Beijing, P.R. China; 6 Aging Research Center, Department of Neurobiology, Care Sciences and Society, Karolinska Institutet-Stockholm University, Solna, Sweden

**Keywords:** Predictive markers, Neuroscience, Prognostic markers

## Abstract

Plasma amyloid-β(Aβ)42/40 ratio, glial fibrillary acidic protein (GFAP), neurofilament light (NfL), and phosphorylated tau181(p-tau181) are promising biomarkers for Alzheimer’s disease (AD)-related pathology. We aimed to explore the longitudinal trajectories of these biomarkers in association with changes in structural brain markers and cognition, and the impact of cognitive reserve on their associations with cognitive function. This cohort study included 1270 individuals (mean age 59.7 years; 58.9% women) derived from the UK Biobank who had data on plasma biomarkers available at baseline (2014–2020); of these, data were available in 904 individuals for brain MRI scans and in 1183 for cognitive function. In 2021–2022, follow-up brain MRI markers and cognitive function were assessed. Plasma Aβ, GFAP, NfL, and p-tau181 were quantified using single-molecule array technology. Brain MRI scans were used to assess atrophic brain measures and white matter microstructures. Cognitive reserve was indexed by education, with college degree or above being defined as high cognitive reserve. Higher baseline plasma GFAP and NfL were significantly associated with brain atrophy and impaired white matter microstructure. The longitudinal increase in plasma GFAP was correlated with accelerated deterioration in processing speed (β = −0.041, *P* = 0.002) and visual attention (β = −0.048, *P* = 0.001), and with impaired white matter microstructure. Importantly, high cognitive reserve significantly mitigated the association between increases in plasma GFAP and NfL and accelerated decline in processing speed. These results indicate that plasma GFAP and NfL are surrogate biomarkers for structural brain health and cognitive health, and that high cognitive reserve may modify the cognitive trajectories associated with Alzheimer’s pathology.

## Introduction

Alzheimer’s disease (AD), as the most common cause of dementia, is pathologically characterized by the deposition of amyloid plaques and formation of neurofibrillary tangles in the brain parenchyma [1]. Clinical studies have shown that AD-related plasma biomarkers, such as plasma amyloid-β (Aβ) 42/40 ratio, phosphorylated tau181 (p-tau181), glial fibrillary acidic protein (GFAP), and neurofilament light chain (NfL), are correlated with the load of AD pathology in central nervous system (CNS) and accelerated progression of the disease [2–7]. Brain atrophy and impaired white matter microstructural integrity are known to be predictive of occurrence and progression of AD. Thus, early detection of structural brain changes is crucial for identifying high-risk individuals for AD, and thus, for implementing preventive interventions to delay AD onset at the early stage.

The amyloid cascade hypothesis proposes that Aβ pathology is an upstream event in AD, driving neocortical tau pathology and neurodegeneration [8]. Additionally, the plasma Aβ42/40 ratio has been shown to be strongly correlated with Aβ-PET imaging status [9, 10]. Several clinic-based studies have shown that plasma NfL and GFAP, which reflect axonal degeneration and reactive astrocytes, respectively [11, 12], are correlated with Aβ pathology in CNS and could predict future cognitive declines and progression of neurodegeneration [13, 14]. However, population-based studies have rarely examined the AD-related plasma biomarkers in relation to brain structures and function of various cognitive domains. In particular, no research has investigated the association of longitudinal trajectories of these plasma biomarkers with dynamic changes in brain structure and cognitive function over time in middle-aged and older adults. This is important not only for clarifying the relationships of trajectories of dynamic changes in AD-related plasma biomarkers, brain structure, and cognitive phenotypes, but also for generalizing the findings from clinical settings of diverse patient populations to the general population.

The meta-analysis of PET studies revealed that Alzheimer’s pathologies (e.g., Aβ) begin to accumulate in the brain 2–3 decades before the clinical onset of AD [15], suggesting that compensation mechanisms could partly explain disparities between clinical and neuropathological phenotypes. Cognitive reserve, which accumulates through lifelong intellectual activities and enriched experiences, has been proposed to explain such disparities [16]. Educational attainment is a widely used proxy for cognitive reserve [17]. Numerous studies have reported that high educational attainment is associated with reduced risk of dementia and AD as well as a slow memory decline [18], in which education is proposed to modulate the impact of AD pathology on cognitive phenotypes. Indeed, autopsy-verified studies have shown that individuals with high educational attainment experience slower cognitive decline even in the presence of substantial AD pathology or gross infarcts [19]. However, the extent to which cognitive reserve may mitigate the relationships between the emerging peripheral biomarkers for AD and cognitive phenotypes remains unclear.

The UK Biobank, a unique resource for biomedical research, offers comprehensive longitudinal data on plasma AD-related biomarkers, specific-domain cognitive function, high-quality brain MRI scans, and measurements of the socioeconomic environment. The UK Biobank brain imaging data included three structural modalities, resting and task-based fMRI, and diffusion imaging [20–22]. The white matter fiber integrity measures in the UK Biobank include the conventional fractional anisotropy (FA) and mean diffusivity (MD) metrics and neurite orientation dispersion and density imaging (NODDI) measures [23]. These measures assessed the integrity of white matter microstructure, neurite density (i.e., intracellular volume fraction; ICVF), extracellular water diffusion (i.e., isotropic volume fraction; ISOVF), and tract complexity or fanning (i.e., orientation dispersion, OD). Leveraging the comprehensive resources of the UK Biobank, we aimed to: (1) investigate the associations of trajectories of plasma AD-related biomarkers (Aβ42/40 ratio, GFAP, NfL, and p-tau181) with longitudinal changes in cognitive function and brain structures; and (2) examine whether cognitive reserve moderates the associations of plasma AD-related biomarkers with cognitive decline in dementia-free middle-aged and older adults.

## Methods

### Study design and study participants

This population-based cohort study used data from the UK Biobank Coronavirus Disease (COVID)-19 repeat imaging study. From 2014–2020, the UK Biobank initiated a multi-modal imaging substudy, with over 45,000 participants undergoing cognitive assessments, structural brain MRI scans, and blood sample collection [21]. Between 2021 and 2022, participants who had previously participated in the UK Biobank imaging study were invited to the COVID-19 repeat imaging study, a substudy that was established to investigate the potential effects of SARS-CoV-2 infection on internal organs by comparing imaging scans taken from participants before and after infection. Compared to participants in the UK Biobank imaging study, those in the COVID-19 repeat imaging study were approximately five years younger and slightly more educated (college or above, 50.8% vs. 48.0%, *P* = 0.049), but the two groups had no significant differences in the distribution of sex or ethnicity. For detailed information about the COVID-19 repeat imaging study, please refer to Resources 2120 and Field 41000 (further details are provided in the online documentation: biobank.ndph.ox.ac.uk/ukb/ukb/docs/casecontrol_covidimaging.pdf). All the UK Biobank participants provided written informed consent, and the study was approved by the Multi-Centre Research Ethics Committee (REC number 11/NW/0382).

In brief, the baseline examination for this study was defined as the time of the first blood sampling for detection of plasma AD-related biomarkers (2014–2020), which included 1270 participants. Among them, data were available in 904 participants for cognitive tests and in 1183 participants for brain MRI scans. Of the 904 participants at baseline with available data on plasma AD-related biomarkers and cognitive tests, 836 (92.5%) had follow-up data on cognitive function; of these, 21 were excluded due to missing covariates, leaving 815 for the analysis involving cognitive outcomes. In addition, 996 (84.2%) of the 1183 baseline participants underwent follow-up brain MRI scans; of these, 14 were excluded due to missing data on covariates, resulting in 982 participants for the analysis of neuroimaging changes. Supplementary Fig. 1 shows the timelines and flowchart of study participants for this analysis.

### Measurements of plasma biomarkers

Blood samples were collected in ethylenediaminetetraacetic acid (EDTA)-coated tubes and immediately centrifuged at 2500 g for 10 min at 4 °C to isolate plasma [24, 25]. Subsequently, the supernatant was then divided into aliquots and stored at −80 °C as soon as possible until further processing. The EDTA plasma samples were transported on dry ice to the University College London UK Dementia Research Institute Fluid Biomarker Lab for assay. Concentrations of plasma AD-related biomarkers were measured on the single molecule array (Simoa) HD-X platform. Plasma p-tau181 was measured using the P-tau 181 V2 Simoa Advantage Assay and plasma Aβ40, Aβ42, GFAP, and NfL were measured using the Neurology 4-Plex E kit. Detailed sample processing, quality control, and storage procedures have been previously reported [26].

### Assessments of cognitive function

Cognitive function was assessed *via* touchscreen on the same day as the MRI scan. Six cognitive tests, including fluid intelligence, paired associate learning, pairs matching, reaction time, symbol digit substitution, and trail making tests (TMTs), were used in the current study [27]. The raw cognitive test scores derived from the UK Biobank database were standardized into z-scores for analysis. These tests were used to evaluate function of cognitive various domains: reasoning (fluid intelligence), memory (paired associate learning), processing speed (reaction time; symbol digit substitution), visual attention (trail making), and executive function (pairs matching). Detailed descriptions of these cognitive tests are available in the Supplementary Methods, and Supplementary Table 1 lists the field IDs.

### Brain MRI acquisition and processing protocol

All neuroimaging biomarkers used in this study were derived from the UK Biobank brain MRI substudy, which was acquired on a standard Siemens Skyra 3.0 T scanner equipped with a standard 32-channel RF receive head coil. T1-weighted images, T2-weighted fluid-attenuated inversion recovery (FLAIR) images, and diffusion MRI data were used for this study [20–22].

Detailed information about the image acquisition, processing, and quality control procedures can be found on the website at https://biobank.ctsu.ox.ac.uk/crystal/crystal/docs/brain_mri.pdf and elsewhere [26, 28]. Total gray matter volume, total white matter volume, and 68 cortical regional parameters were extracted from T1-weighted imaging according to the Desikan-Killiany atlas, and 16 subcortical regional parameters were extracted according to the ASEG atlas. Total gray matter volume and total white matter volume were normalized for head size [28]. The total intracranial volume, as determined by the ASEG atlas and adjusted for components other than gray and white matter, was utilized as a covariate in the neuroimaging analyses. Furthermore, we extracted 135 white matter weighted tract-averaged measures, including FA, MD, and three NODDI indices [23] (ICVF, ISOVF, and OD), from the preprocessed diffusion MRI by averaging parameters across 27 white matter tract regions.

### Measurement of cognitive reserve

Cognitive reserve was indexed by educational attainment, the most frequently used proxy for cognitive reserve. Participants were classified into two groups based on their educational level: (1) higher cognitive reserve if the participants received a college degree or above; and (2) lower cognitive reserve that included a national vocational qualification, higher national diploma, higher national certificate, a levels/advanced subsidiary levels or equivalent examinations, or no educational qualifications [29].

### Assessments of covariates

We considered the following factors assessed at baseline as covariables [30–32]: age (Field 21022), sex (Field 31), ethnicity (White or non-White; Field 21000), the townsend deprivation index (Field 189; referring to an area-based measure of socioeconomic deprivation), smoking status (never, former, or current; Field 20116), alcohol intake (daily or almost daily, 3–4 times a week, 1–2 times a week, 1–3 times a month, on occasions, or never; Field 1558), and health-related factors. Health-related factors included hypertension (yes or no), diabetes (yes or no), cardiovascular arterial diseases (CAD; yes or no), apolipoprotein E (*APOE*) ε4 allele (carriers or non-carriers), and a history of COVID-19 infection (yes or no; Field 41000). Hypertension was defined as systolic blood pressure (SBP) ≥ 140 mmHg (Field 4080) or diastolic blood pressure (DBP) ≥ 90 mmHg (Field 4079), use of antihypertension medication (Field 6177), or medical records (ICD-10 codes I10 to I13 and I15). Diabetes was determined based on medical records (ICD-10 codes E10 to E14), glycated hemoglobin ≥6.5% (Field 30750), or the use of antidiabetic drugs (Field 6177). CAD was identified using medical records (ICD-10 codes I20 to I25). The *APOE* genotypes were determined using single nucleotide polymorphism data for rs429358 and rs7412.

### Statistical analysis

We conducted descriptive analyses to examine the characteristics of study participants by sex. We presented the mean and standard deviation (SD) for the continuous variables, and the number and proportion for categorical variables. We employed t-test to compare normally distributed continuous variables and chi-square test for categorical variables. Participants were included in the analytical sample if they had data available in at least one of the five plasma AD-related biomarkers (Aβ40, Aβ42, GFAP, NfL, and p-tau181), but those with missing data on outcome variables or covariates were excluded from the analysis (Supplementary Fig. 1).

To study associations of plasma biomarkers with longitudinal changes in brain structures and cognition, we used linear mixed-effects (LME) models with the interaction term between time and plasma biomarker as the independent variable. The random effect included random intercept, allowing individual differences to be reflected at baseline. The observational time was defined as the number of years from the date of baseline examination to the date of follow-up assessment. To facilitate comparisons across different scales, all continuous independent variables were standardized into z-scores before being included in the models. Plasma GFAP, NfL, and p-tau181 concentrations were log-transformed before standardization to address skewed distributions and improve model fit. All models were adjusted for baseline age, sex, ethnicity, townsend deprivation index, assessment center, smoking status, alcohol use, *APOE* ε4 allele, hypertension, diabetes, and coronary artery disease (CAD), and history of COVID-19 infection. For the analysis involving structural brain measures, we further adjusted for total intracranial volume to account for individual variability in brain size.

We further explored the potential effect modification of age, sex, history of COVID-19 infection, and educational attainment on the associations between plasma AD-related biomarkers and longitudinal cognitive changes using LME models, in which the three-way interaction term of age (<65 and ≥65 years), sex (male and female), COVID-19 infection (yes and no) or educational attainment (high and low) with plasma AD-related biomarker levels and follow-up time was included together with the individual factors in the models. Stratified analyses were conducted when the statistically significant interaction (*P* for interaction term <0.05) was detected.

The Benjamini-Hochberg false discovery rate (FDR) method was used to correct for multiple comparisons, and the FDR-corrected *P* value < 0.05 was considered statistically significant. R version 4.2.3 (R Foundation for Statistical Computing, Vienna, Austria) was used for all statistical analyses, and LME models were fitted using the lme4 package in R [33].

## Results

### Baseline characteristics of study participants

The cohort study comprised 1270 individuals (mean age 59.71 years, 58.88% female) at baseline and the median follow-up period was 2.68 years (range: 1.04–7.22 years). Compared to females, male participants were older and more likely to consume alcohol and have hypertension, diabetes, and CAD. However, there were no significant sex differences in educational level, racial distribution, *APOE* ε4 allele, smoking, or history of COVID-19 infection (Table 1).

**Table 1:** Baseline characteristics of study participants.

Characteristics	Total sample	Female	Male	*P*-value^*^
	(*n* = 1270)	(*n* = 672)	(*n* = 598)	
Age (years)	59.71 (7.44)	58.88 (6.84)	60.65 (7.97)	<0.001
Education, n (%)				0.520
College or above	641 (50.8)	346 (51.7)	295 (49.7)	
Others	629 (49.2)	326 (48.3)	303 (51.3)	
Townsend deprivation index	−1.57 (2.81)	−1.47 (2.87)	−1.69 (2.74)	0.167
Ethnicity, n (%)				
White	1168 (92.0)	622 (92.6)	546 (91.3)	0.473
Non-White	102 (8.0)	50 (7.4)	52 (8.7)	
*APOE* ε4 carrier, n (%)	257 (20.2)	141 (21.0)	116 (19.4)	0.528
Smoking status, n (%)				0.075
Never	816 (64.7)	450 (67.4)	366 (61.6)	
Former	402 (31.9)	199 (29.8)	203 (34.2)	
Current	44 (3.5)	19 (2.8)	25 (4.2)	
Alcohol intake frequency, n (%)				<0.001
Daily or almost daily	199 (15.7)	92 (13.8)	107 (18.0)	
3–4 times/week	382 (30.2)	182 (27.2)	200 (33.6)	
1–2 times/week	366 (29.0)	193 (28.8)	173 (29.1)	
1–3 times/month	149 (11.8)	90 (13.5)	59 (9.9)	
Occasions	110 (8.7)	82 (12.3)	28 (4.7)	
Never	58 (4.6)	30 (4.5)	28 (4.7)	
COVID-19, n (%)	636 (50.1)	336 (50.0)	300 (50.2)	0.997
Hypertension, n (%)	645 (50.8)	260 (38.7)	385 (64.4)	<0.001
Diabetes, n (%)	51 (4.0)	15 (2.2)	36 (6.0)	0.001
CAD, n (%)	58 (4.6)	16 (2.4)	42 (7.0)	<0.001

Data are mean (standard deviation), unless otherwise specified.

*APOE*, apolipoprotein E gene, *CAD* cardiovascular arterial disease.

^*^P-value is for the test of differences between males and females.

### Associations of plasma AD-related biomarkers with cognitive decline

**Fig. 1:**
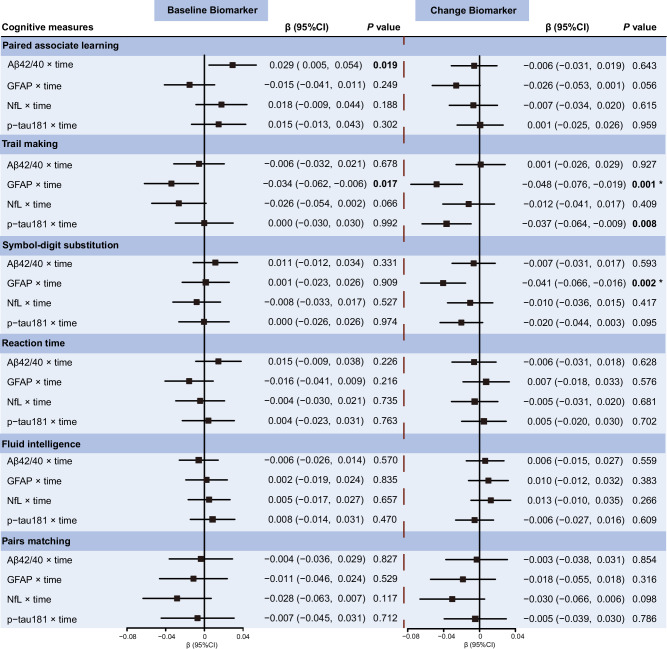
Associations of baseline and longitudinal changes in plasma AD-related biomarkers with cognitive decline over time by using linear mixed-effects models. Beta-coefficients (95% confidence intervals) were derived from the linear mixed effects-models that were adjusted for sex, age, ethnicity, townsend deprivation index, assessment center, smoking status, alcohol use, *APOE* ε4 allele, hypertension, diabetes, cardiovascular arterial disease, and history of COVID-19 infection. Significance levels were corrected for multiple comparisons using the Benjamini-Hochberg FDR method: ^*^FDR-corrected *P* < 0.05. Aβ amyloid-β, GFAP glial fibrillary acidic protein, NfL neurofilament light chain, p-tau181 phosphorylated tau 181, CI confidence interval.

We first examined the longitudinal associations of plasma AD-related biomarkers assessed at baseline with changes in cognitive function from baseline to follow-up. Controlling for sex, age, ethnicity, townsend deprivation index, assessment center, smoking status, alcohol use, *APOE* ε4 allele, hypertension, diabetes, CAD, and history of COVID-19 infection, a lower plasma Aβ42/40 ratio at baseline was significantly associated with a faster decline in memory performance, as measured by the paired associate learning test (β = −0.029, *P* = 0.019) (Fig. 1). Additionally, elevated plasma GFAP at baseline was associated with a greater rate of decline in visual attention, assessed through the TMTs (β = −0.034, *P* = 0.017). However, these associations were no longer statistically significant after FDR correction for multiple comparisons. Overall, baseline plasma p-tau181 and NfL were not significantly associated with longitudinal changes in cognitive function. Corrected *P*-values for these findings are detailed in Supplementary Table 2. We detected a statistical interaction between plasma NfL and p-tau181 with age groups on the decline in processing speed (*P* for interaction = 0.024 and 0.041, respectively). Further stratified analysis revealed that the associations of elevated plasma NfL and p-tau181 with processing speed decline were significant only in individuals aged over 65 years (Supplementary Tables 3). We did not observe any interaction effect between plasma biomarkers and sex on cognitive decline (Supplementary Tables 4). We observed an interaction between plasma GFAP and COVID-19 infection on the decline in visual attention (*P* for interaction = 0.029), with elevated plasma GFAP significantly associated with visual attention decline only in individuals without a history of COVID-19 infection (Supplementary Table 5).

Next, we further examined the correlations of longitudinal changes in plasma AD-related biomarkers with changes in cognitive function over time. We found that an increase in plasma GFAP over time was significantly correlated with accelerated declines in processing speed and visual attention, as assessed by the symbol-digit substitution and TMTs, respectively (β = −0.041, *P* = 0.002 for processing speed; β = −0.048, *P* = 0.001 for visual attention; Fig. 1). Similarly, an increase in plasma p-tau181 over time was correlated with an accelerated decline in visual attention, as measured by the TMTs (β = −0.037, *P* = 0.008; Fig. 1). After applying FDR corrections for multiple comparisons, these associations remained statistically significant. Corrected *P*-values for these findings are detailed in Supplementary Table 5.

### Associations of plasma AD-related biomarkers with structural brain changes

**Fig. 2:**
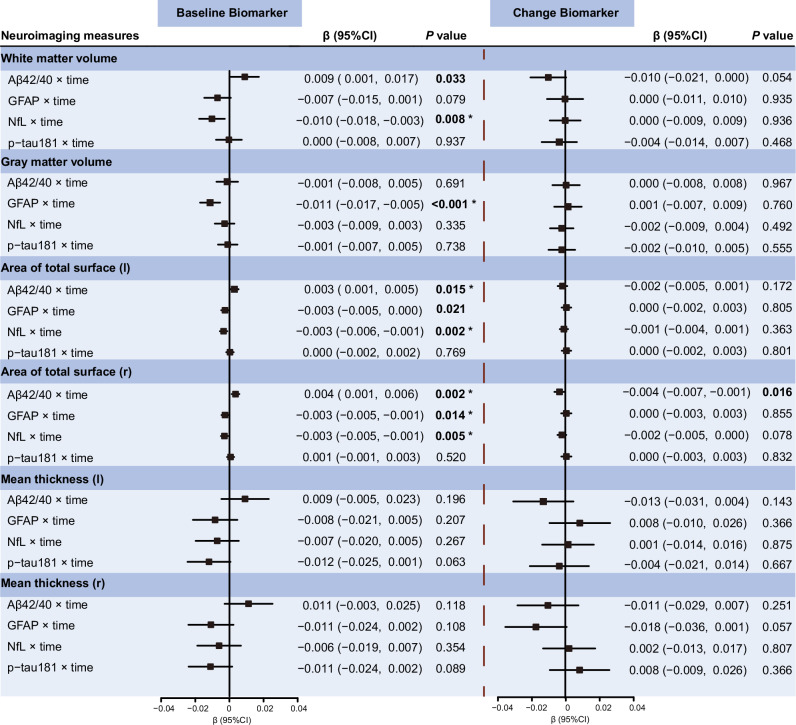
Associations of baseline and longitudinal changes in plasma AD-related biomarkers with longitudinal changes in global structural brain measures over time by using linear mixed-effects models. Beta-coefficients (95% confidence intervals) were derived from the linear mixed effects-models that were adjusted for sex, age, ethnicity, townsend deprivation index, assessment center, smoking status, alcohol use, APOE ε4 allele, hypertension, diabetes, cardiovascular arterial disease, history of COVID-19 infection, and total intracranial volume. Significance levels were corrected for multiple comparisons using the Benjamini-Hochberg FDR method: ^*^FDR-corrected *P* < 0.05. Aβ amyloid-β, GFAP glial fibrillary acidic protein, NfL neurofilament light chain, p-tau181 phosphorylated tau 181, CI confidence interval.

We first examined the relationship between baseline plasma AD-related biomarkers and longitudinal changes in global structural brain MRI measures (total gray matter volume, total white matter volume, total cortical area, and total cortical thickness). Elevated baseline plasma GFAP was significantly associated with reductions in total gray matter volume (β = −0.011, *P* < 0.001) and total cortical area (left hemisphere: β = −0.003, *P* = 0.021; right hemisphere: β = −0.003, *P* = 0.014) after adjusting for covariates. Similarly, higher baseline plasma NfL was correlated with reduced total white matter volume (β = −0.010, *P* < 0.001) and total cortical area (left hemisphere: β = −0.003, *P* = 0.002; right hemisphere: β = −0.003, *P* = 0.005). In addition, a lower plasma Aβ42/40 ratio was correlated with reduced total white matter volume (β = 0.009, *P* = 0.033) and total cortical area (left hemisphere: β = 0.003, *P* = 0.015; right hemisphere: β = 0.004, *P* = 0.002) (Fig. 2; Supplementary Table 6). However, when examining correlations between changes in plasma AD biomarkers with changes in structural brain measures, longitudinal changes in plasma AD-related biomarkers were not significantly correlated with longitudinal changes in any of the examined global structural brain MRI measures (Supplementary Table 7).

**Fig. 3:**
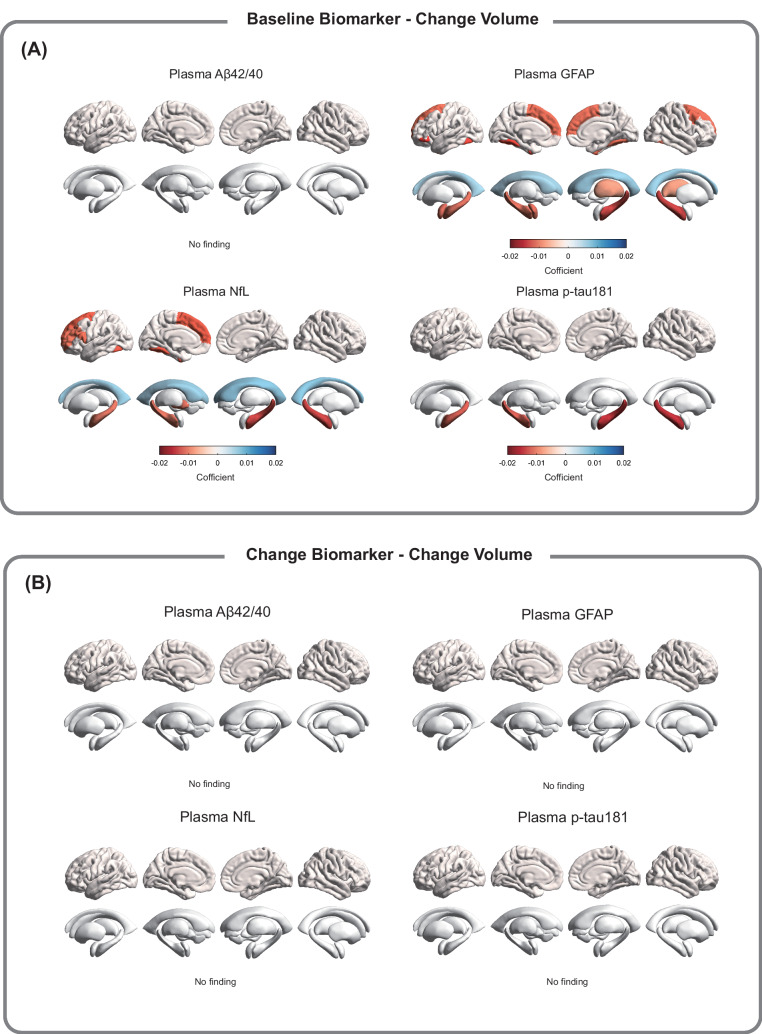
Associations of baseline and longitudinal changes in plasma AD-related biomarkers with longitudinal changes in brain volume. Panel **A** demonstrates the association between baseline plasma levels and cortical and subcortical volume changes, while panel **B** illustrates the association between changes in plasma levels over time and corresponding changes in cortical and subcortical volumes (^*^FDR-corrected *P* < 0.05). Beta-coefficients (95% confidence intervals) were derived from the linear mixed effects-models that were adjusted for sex, age, ethnicity, townsend deprivation index, assessment center, smoking status, alcohol use, APOE ε4 allele, hypertension, diabetes, cardiovascular arterial disease, history of COVID-19 infection, and total intracranial volume. Aβ amyloid-β, GFAP glial fibrillary acidic protein, NfL neurofilament light chain, p-tau181, phosphorylated tau 181.

Secondly, we examined the associations between baseline plasma AD-related biomarkers and longitudinal changes in specific regional brain MRI measures (e.g., 68 cortical regions and 16 subcortical regions). Elevated plasma GFAP and NfL at baseline were significantly associated with accelerated cognitive decline in the cortical volumes of the fusiform cortex, superior frontal gyrus, and pars opercularis after FDR correction (*P* < 0.05) (Fig. 3A; Supplementary Table 8) as well as with accelerated cognitive decline in cortical areas in the fusiform cortex, superior frontal gyrus, and pars opercularis (Supplementary Table 9). Additionally, increased plasma GFAP, NfL, and p-tau181 at baseline were significantly associated with reduced hippocampal volumes after FDR correction (*P* < 0.05) (Fig. 3A; Supplementary Table 10). However, no significant associations were observed between longitudinal changes in plasma AD-related biomarkers and changes in brain cortical and subcortical volumes (Fig. 3B; Supplementary Tables 10, 11).

**Fig. 4:**
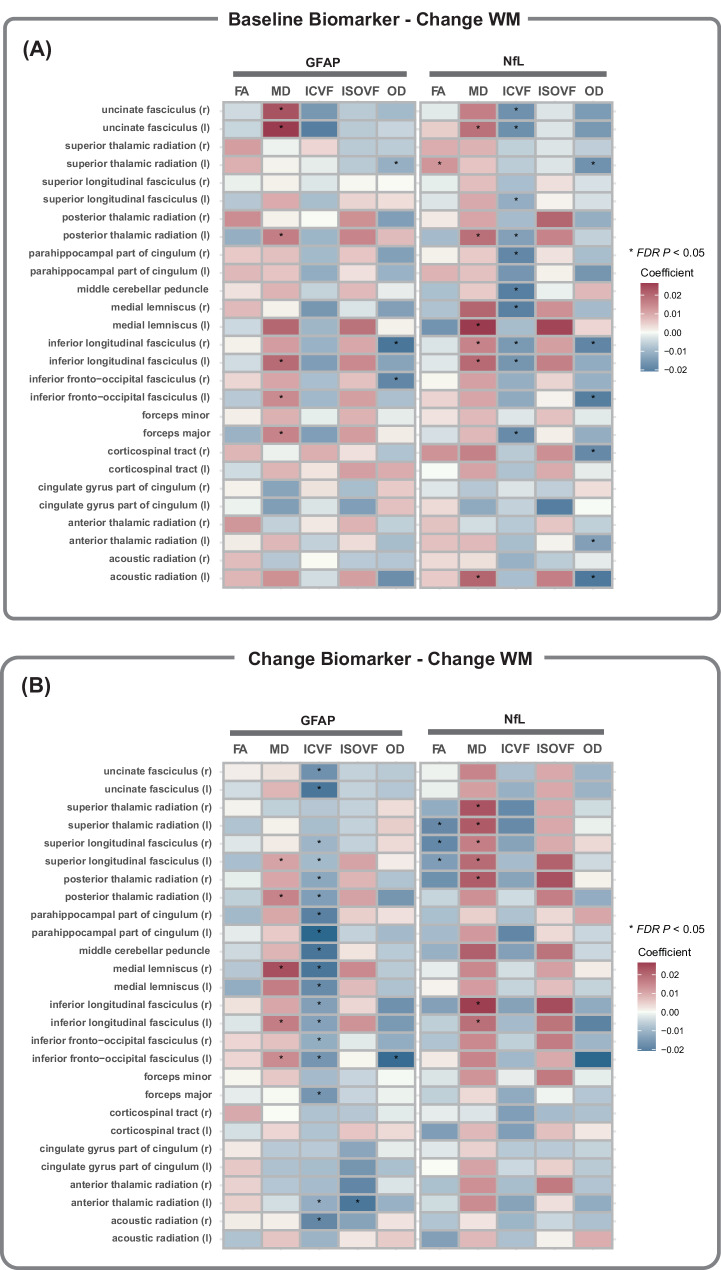
Associations of baseline and longitudinal changes in plasma AD-related biomarkers with longitudinal changes in white matter microstructure. Panel **A** demonstrates the association between baseline plasma levels and changes in white matter microstructure, while panel **B** illustrates the association between changes in plasma levels over time and corresponding changes in white matter microstructure (^*^FDR-corrected *P* < 0.05). Beta-coefficients (95% confidence intervals) were derived from the linear mixed effects-models that were adjusted for sex, age, ethnicity, townsend deprivation index, assessment center, smoking status, alcohol use, APOE ε4 allele, hypertension, diabetes, cardiovascular arterial disease, history of COVID-19 infection, and total intracranial volume. GFAP glial fibrillary acidic protein, NfL neurofilament light chain, FA fractional anisotropy, MD mean diffusivity, ICVF intracellular volume fraction, ISOVF isotropic volume fraction, OD orientation dispersion, r right, l left.

Finally, we evaluated the relationship between baseline and longitudinal changes in plasma AD-related biomarkers and longitudinal changes in 135 white matter integrity phenotypes, including FA, MD, ICVF, ISOVF, and OD that were extracted by averaging parameters across 27 white matter tract regions. Higher plasma GFAP at baseline was significantly associated with an increased MD in six white matter tract regions, with the uncinate fasciculus showing the strongest association, followed by forceps minor, and superior longitudinal fasciculus (Fig. 4A; Supplementary Table 12). Similarly, plasma NfL at baseline showed strong associations with MD and ICVF across various brain regions, particularly in uncinate fasciculus, posterior thalamic radiation and inferior longitudinal fasciculus. Moreover, longitudinal increases in plasma GFAP over time were significantly associated with reduced ICVF in 18 white matter tract regions (Fig. 4B; Supplementary Table 13).

### Modifying effect of cognitive reserve on the association of plasma AD-related biomarkers with cognitive decline

**Fig. 5:**
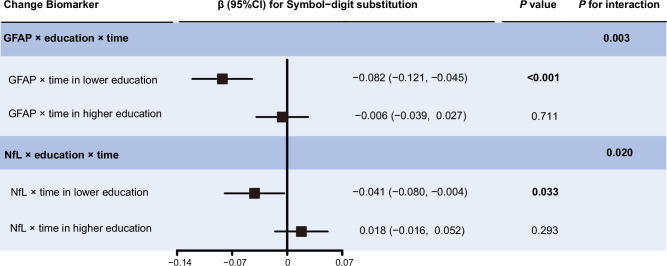
Interactive associations of educational attainment and elevated plasma AD-related biomarkers on cognitive decline by using linear mixed-effects models. Beta-coefficients (95% confidence intervals) were derived from the linear mixed effects-models that were adjusted for sex, age, ethnicity, townsend deprivation index, assessment center, smoking status, alcohol use, APOE ε4 allele, hypertension, diabetes, cardiovascular arterial disease, and history of COVID-19 infection. GFAP glial fibrillary acidic protein, NfL neurofilament light chain, CI confidence interval.

We detected a statistical interaction between cognitive reserve with elevated plasma GFAP and NfL on the decline in processing speed (for the interaction term: *P* = 0.003 and *P* = 0.020, respectively) (Fig. 5; Supplementary Table 14). Further stratified analysis by cognitive reserve levels revealed that increased plasma GFAP and NfL were significantly associated with an accelerated decline in processing speed z-scores in individuals with lower cognitive reserve (for high plasma GFAP, β = −0.082, *P* < 0.001; for high plasma NfL, β = −0.041, *P* = 0.033), but not in those with higher cognitive reserve (for high plasma GFAP, β = −0.006, *P* = 0.711; for high plasma NfL, β = 0.018, *P* = 0.293) (Fig. 5).

## Discussion

In this prospective population-based cohort study within the UK Biobank, we found that (1) longitudinal increases in plasma GFAP were correlated with accelerated declines in processing speed and visual attention, as well as impaired integrity of white matter tracts; (2) higher baseline plasma GFAP and NfL were associated with lower cerebral cortical and subcortical volumes as well as reduced microstructural integrity of white matter tracts; and (3) higher educational attainment could mitigate the associations of increased plasma NfL and GFAP with an accelerated decline in processing speed. Taken together, our cohort study provides additional evidence that trajectories of plasma AD-related biomarkers are associated with impaired white matter microstructural integrity, brain atrophy, and accelerated cognitive decline in a cognitively normal middle-aged and older population and that cognitive reserve could mitigate the association between plasma AD-related biomarkers and cognitive phenotypes.

Previous studies have shown that higher plasma GFAP is associated with global cognitive decline in older adults [7, 34]. Our study extends these findings by demonstrating that longitudinal increases in plasma GFAP are associated with accelerated cognitive declines specifically in the domains of processing speed and visual attention among a relatively young cohort. These two cognitive domains are particularly sensitive to pathological changes in brain regions such as the frontal and fusiform areas, which are highly vulnerable to AD-related pathology, and thus, are related to plasma GFAP. Similarly, we further found that a lower plasma Aβ42/40 ratio was associated with memory loss, and that elevated plasma p-tau181 was correlated with accelerated decline in visual attention, which is in line with the previous reports of studies in the clinical settings of patients with cognitive impairment [35–37]. Of note, some of our observed associations, particularly those involving the Aβ42/Aβ40 ratio and plasma p-tau181, could not survive the FDR correction, partly due to the facts that our study cohort was relatively young and healthy, and had relatively small variations in their cognitive tests, thus, our analytical sample might not be large enough to reveal a weak-to-moderately strong association that could survive the multiple comparison correction. In addition, we found no association between plasma NfL levels and cognitive decline, which is inconsistent with previous findings from clinic-based studies of patients with mild cognitive impairment [38]. Differences in the study settings and characteristics of study populations might partly contribute to the discrepant findings. For instance, studies from the clinic settings usually included patients with cognitive disorders who were at advanced clinical stages with a high neuropathological burden, whereas our study cohort consisted of young and dementia-free participants who had a relatively low burden of neurodegenerative pathology in the brain. Indeed, our research also found that elevated plasma NfL and p-tau181 were linked to declines in processing speed only in older adults (i.e., ≥65 years of age), but not in younger adults (<65 years of age), further emphasizing the potential role of age in amplifying the association between neuropathological loads and cognitive phenotypes.

Additionally, the comprehensive data on structural brain measures in the UK Biobank could help capture the complexity of brain structure and function. Our study revealed that baseline plasma GFAP and NfL were associated with longitudinal changes in specific brain regions that are critical to AD occurrence and progression, such as the fusiform gyrus, frontal lobes, and hippocampus [39, 40]. Consistent with the reports from studies of older adults [41–44], findings from our study of a younger cohort highlight the potential of plasma GFAP and NfL as biomarkers for early structural changes in these key brain regions. Furthermore, we found that plasma p-tau181 was also predictive of hippocampal atrophy, which aligns with previous findings [45] and further emphasizes the potential of plasma p-tau181 as a biomarker for AD progression. It is worth noting that the longitudinal increase in plasma biomarkers (e.g., GFAP) was correlated with accelerated cognitive decline in processing speed and visual attention but not with brain atrophy. This suggests that cognitive measures may be more sensitive than structural brain measures to dynamic changes in plasma GFAP. In addition, we found that higher baseline plasma biomarkers (e.g., GFAP, NfL, and p-tau181) were associated with brain atrophy but not with cognitive decline. This suggests that plasma GFAP, NfL, and p-tau181 appear to be predictive structural brain alterations in middle-ages and older adults and that the relatively young age of the cohort and a short follow-up period may partly contribute to the lack of longitudinal associations of these plasma biomarkers with cognitive deterioration. Taken together, the mechanisms underlying the complex relationships between longitudinal trajectories of plasma biomarkers and structural brain changes and cognitive decline are not fully understood and warrant further investigation in large-scale long-term follow-up studies.

Notably, we expanded our analysis beyond traditional DTI metrics such as FA and MD by incorporating advanced measures from NODDI to comprehensively assess white matter microstructure such as ICVF, ISOVF, and OD [23]. We found that increased plasma GFAP was consistently associated with higher MD across six distinct white matter tract regions. Among these brain regions, the uncinate fasciculus is crucial in visual attention [46, 47], while the forceps minor, uncinate fasciculus, and superior longitudinal fasciculus are related to AD [48, 49]. Furthermore, longitudinal increases in plasma GFAP were correlated with a more rapid loss of ICVF in these brain regions. Additionally, elevated plasma NfL was strongly associated with greater free water diffusion in damaged tissues as indicated by increased MD and with greater loss of neurite density as indicated by decreased ICVF across various brain regions. Taken together, our results support the notion that plasma GFAP and NfL are sensitive biomarkers for neurodegeneration, including brain atrophy and impaired integrity of white matter microstructure.

We further found that cognitive reserve could modify the association between plasma AD-related biomarkers and cognitive decline, such that elevated plasma NfL and GFAP were associated with an accelerated decline in processing speed only among individuals with lower cognitive reserve, but not among those with higher cognitive reserve. This finding aligns with the view that high cognitive reserve could enhance an individual’s ability to cope with neuropathological accumulations in the brain such as Aβ, thereby maintaining cognitive function [50, 51]. Our study further extends the previous findings by showing that higher cognitive reserve might modify the cognitive phenotypes due to increased plasma NfL and GFAP, which reflect the accumulation of neurodegeneration and neuroinflammation in the brain. This supports the view that cognitive reserve could help maintain cognitive function among middle-aged and older adults who are free of dementia. The mechanisms underlying the compensation effect of cognitive reserve on the plasma NfL- and GFAP-related cognitive consequences are not fully understood. Cognitive reserve may induce various neuroplastic responses, such as synaptogenesis and neurogenesis, to compensate for the negative impacts of neuropathology, allowing individuals to withstand more severe damage in the brain before manifesting symptoms of cognitive impairment [52]. Additionally, higher cognitive reserve can promote cognitive function and enhance the efficiency, capacity, or flexibility of neural networks in the brain [53]. Consequently, individuals with higher cognitive reserve may deploy more alternative neural networks to maintain cognitive function.

The UK Biobank included multidimensional data, such as repeated measurements of plasma biomarkers, cognitive function, and multimodal brain imaging markers. Thus, we were able to comprehensively evaluate the complex relationships of trajectories of plasma AD-related plasma biomarkers, cognitive function, and brain structures. Nevertheless, some limitations of our study should be acknowledged and discussed. First, the generalizability of research findings from the UK Biobank might be limited, particularly given the less deprived living conditions of the study participants compared to the broader UK population [54]. Furthermore, participants of the COVID-19 repeat imaging study were slightly healthier than those of the overall UK Biobank cohort, which might lead to underestimation of the true associations of plasma biomarkers with measures of cognitive trajectories and structural brain alterations. Second, some data on health status and lifestyles were ascertained through self-report, which might lead to measurement errors. Third, despite our adjustments for a range of potential confounders, residual confounding from unmeasured factors (e.g., genetic predispositions and environmental exposures) and from imperfect assessments of some confounders (e.g., self-reported lifestyle factors) could not be completely ruled out. Fourth, participants of this study were longitudinally assessed only at two time points. Additional follow-up visits would enable a more detailed characterization of neurodegenerative, brain structural, and cognitive trajectories. Finally, although educational attainment is the most important source and widely used proxy for cognitive reserve, it may not adequately capture the diversity and depth of cognitive reserve. Therefore, future research that adopts a more comprehensive assessment approach of cognitive reserve would enhance our understanding of its role in modulating the relationships of neuropathological load in the brain and cognitive phenotypes.

## Conclusion

This population-based prospective cohort study demonstrates that trajectories of plasma GFAP and NfL are associated with brain atrophy, impaired white matter microstructure integrity, and accelerated cognitive decline in middle-aged and older adults, and that cognitive reserve can modify the associations between these plasma biomarkers and cognitive phenotypes. These findings suggest that plasma AD-related biomarkers are valuable prognostic indicators for the progression of neuropathology in the brain and cognitive phenotypes and that high cognitive reserve may contribute to cognitive health in pathological brain aging.

## Supplementary information

Supplementary Materials

## Data Availability

The analyses presented in this study were conducted under UK Biobank application number 91982. The data used in this research is subject to the following access restrictions: The UK Biobank is a comprehensive biomedical resource containing detailed genetic and health data from over 500,000 participants across the UK. This database is continuously updated with new information and is accessible to authorized researchers worldwide, supporting research on common and life-threatening diseases. To access these datasets, please visit https://www.ukbiobank.ac.uk/.

## Code availability

The code used for linear mixed-effects models is an adaptation of the R package lme4 (https://github.com/lme4/lme4) and has been made available through the GitHub repository: https://github.com/huangdouz/ukb_biomrker.
