# Supplementary material for: Associations between trajectories of plasma biomarkers for Alzheimer’s disease, brain structures, and cognitive function: a prospective cohort study in the UK Biobank
Source: Mol Psychiatry. 2025 Aug 28;31(2):749–60. doi: 10.1038/s41380-025-03166-y (PMC12815683; doi:10.1038/s41380-025-03166-y)
Supplement: Supplementary file 1 — Supplementary Materials [file 41380_2025_3166_MOESM1_ESM.docx]

Supplementary Materials

**Associations between trajectories of plasma biomarkers for Alzheimer’s disease, brain structures, and cognitive function: a prospective cohort study in the UK Biobank**

Huang X, et al.

**Contents**

[Supplementary Methods 3](#_Toc188870551)

[Supplementary Tables 4](#_Toc188870552)

[Supplementary Table 1. The UK Biobank showcase variables used in the study. 4](#_Toc188870553)

[Supplementary Table 2. Associations of baseline plasma AD-related biomarkers with longitudinal cognitive decline by using linear mixed-effects models. 5](#_Toc188870554)

[Supplementary Table 3. Interactive associations of age and elevated plasma AD-related biomarkers on cognitive decline. 6](#_Toc188870555)

[Supplementary Table 4. Interactive associations of sex and elevated plasma AD-related biomarkers on cognitive decline. 7](#_Toc188870556)

[Supplementary Table 5. Interactive associations of COVID-19 and elevated plasma AD-related biomarkers on cognitive decline. 8](#_Toc188870557)

[Supplementary Table 6. Associations of longitudinal plasma AD-related biomarkers with longitudinal cognitive decline by using linear mixed-effects models. 9](#_Toc188870558)

[Supplementary Table 7. Associations of baseline plasma AD-related biomarkers with longitudinal changes in total brain structure by using linear mixed-effects models. 10](#_Toc188870559)

[Supplementary Table 8. Associations of longitudinal plasma AD-related biomarkers with longitudinal changes in total brain structure by using linear mixed-effects models. 11](#_Toc188870560)

[Supplementary Table 9. Associations of baseline plasma AD-related biomarkers with longitudinal changes in cortical gray volumes by using linear mixed-effects models. 12](#_Toc188870561)

[Supplementary Table 10. Associations of baseline plasma AD-related biomarkers with longitudinal changes in cortical areas by using linear mixed-effects models. 19](#_Toc188870562)

[Supplementary Table 11. Associations of baseline plasma AD-related biomarkers with longitudinal changes in subcortical brain volumes by using linear mixed-effects models. 26](#_Toc188870563)

[Supplementary Table 12. Associations of longitudinal plasma AD-related biomarkers with longitudinal changes in cortical gray volumes by using linear mixed-effects models. 28](#_Toc188870564)

[Supplementary Table 13. Associations of longitudinal plasma AD-related biomarkers with longitudinal changes in subcortical brain volumes by using linear mixed-effects models. 35](#_Toc188870565)

[Supplementary Table 14. Associations of baseline plasma AD-related biomarkers with longitudinal changes in white matter microstructure by using linear mixed-effects models. 37](#_Toc188870566)

[Supplementary Table 15. Associations of longitudinal plasma AD-related biomarkers with longitudinal changes in white matter microstructure by using linear mixed-effects models. 50](#_Toc188870567)

[Supplementary Table 16. Interactive associations of educational attainment and elevated plasma AD-related biomarkers on cognitive decline. 63](#_Toc188870568)

[Supplementary Figure 1. Study timeline (A) and flowchart (B) of study participants. 64](#_Toc188870569)

# Supplementary Methods

**Assessments of cognitive function**

**1. Fluid Intelligence:** This assessment evaluated problem-solving skills, logic, and reasoning ability. It consisted of a 13-item task presented on a touch screen.

**2. Paired Associate Learning Test:** Participants were presented with 12 pairs of words for 30 seconds. After an interval, they were shown the first word of 10 pairs and asked to select the matching second word from four alternatives.

**3. Pair Matching Task:** In this task, participants were tasked with memorizing the positions of six pairs of matching cards presented simultaneously. They then had to identify the locations of the matching pairs with minimal attempts. Errors were recorded to assess performance.

**4. Symbol Digit Substitution Task:** Participants were presented with two grids: one linking symbol to single-digit integers, and another containing only symbols. They were required to indicate the numbers attached to each symbol in the second grid using the first grid as a reference.

**5. Reaction Time Task:** Similar to the card game Snap, participants were instructed to press a button when two simultaneously presented cards matched.

**6. Trail-Making Task:** Participants were presented with sets of digits or letters in circles scattered around the screen and asked to click on them sequentially according to a specific algorithm.

# Supplementary Tables

# Supplementary Table 1. The UK Biobank showcase variables used in the study.

| Cognitive function | **Field ID** | **Instance** |
| --- | --- | --- |
| Fluid intelligence | 20016 | Instance2 Instance3 |
| Paired associate learning | 20197 | Instance2 Instance3 |
| Pairs matching | 399 | Instance2 Instance3 |
| Reaction time | 20023 | Instance2 Instance3 |
| Symbol digit substitution | 23324 | Instance2 Instance3 |
| Trail making | 6348 | Instance2 Instance3 |

# Supplementary Table 2. Associations of baseline plasma AD-related biomarkers with longitudinal cognitive decline by using linear mixed-effects models.

| **Plasma biomarkers** | **Cognitive function** | **β (95% CI)** | ***P*-value** | **FDR-corrected *P*** |
| --- | --- | --- | --- | --- |
| Aβ42/40 | Reaction time | 0.015 (-0.009, 0.038) | 0.226 | 0.746 |
| Aβ42/40 | Symbol digit substitution | 0.011 (-0.012, 0.034) | 0.331 | 0.795 |
| Aβ42/40 | Trail making | -0.006 (-0.032, 0.021) | 0.678 | 0.954 |
| Aβ42/40 | Pairs matching | -0.004 (-0.036, 0.029) | 0.827 | 0.954 |
| Aβ42/40 | Paired associate learning | 0.029 (0.005, 0.054) | 0.019 | 0.233 |
| Aβ42/40 | Fluid intelligence | -0.006 (-0.026, 0.014) | 0.570 | 0.954 |
| GFAP | Reaction time | -0.016 (-0.041, 0.009) | 0.216 | 0.746 |
| GFAP | Symbol digit substitution | 0.001 (-0.023, 0.026) | 0.909 | 0.992 |
| GFAP | Trail making | -0.034 (-0.062, -0.006) | 0.017 | 0.233 |
| GFAP | Pairs matching | -0.011 (-0.046, 0.024) | 0.529 | 0.954 |
| GFAP | Paired associate learning | -0.015 (-0.041, 0.011) | 0.249 | 0.746 |
| GFAP | Fluid intelligence | 0.002 (-0.019, 0.024) | 0.835 | 0.954 |
| NFL | Reaction time | -0.004 (-0.030, 0.021) | 0.735 | 0.954 |
| NFL | Symbol digit substitution | -0.008 (-0.033, 0.017) | 0.527 | 0.954 |
| NFL | Trail making | -0.026 (-0.054, 0.002) | 0.066 | 0.528 |
| NFL | Pairs matching | -0.028 (-0.063, 0.007) | 0.117 | 0.701 |
| NFL | Paired associate learning | 0.018 (-0.009, 0.044) | 0.188 | 0.746 |
| NFL | Fluid intelligence | 0.005 (-0.017, 0.027) | 0.657 | 0.954 |
| p-tau181 | Reaction time | 0.004 (-0.023, 0.031) | 0.763 | 0.954 |
| p-tau181 | Symbol digit substitution | 0.000 (-0.026, 0.026) | 0.974 | 0.992 |
| p-tau181 | Trail making | 0.000 (-0.030, 0.030) | 0.992 | 0.992 |
| p-tau181 | Pairs matching | -0.007 (-0.045, 0.031) | 0.712 | 0.954 |
| p-tau181 | Paired associate learning | 0.015 (-0.013, 0.043) | 0.302 | 0.795 |
| p-tau181 | Fluid intelligence | 0.008 (-0.014, 0.031) | 0.470 | 0.954 |

Abbreviations: Aβ, amyloid-β; GFAP, glial fibrillary acidic protein; NfL, neurofilament light chain; p-tau181, tau phosphorylated at threonine 181; CI, confidence interval; FDR, false discovery rates.

Model was adjusted for sex, age, ethnicity, townsend deprivation index, assessment center, smoking status, alcohol use, family history of dementia, *APOE* ε4 allele*,* hypertension, diabetes, cardiovascular arterial disease, and history of COVID-19 infection.

# Supplementary Table 3. Interactive associations of age and elevated plasma AD-related biomarkers on cognitive decline.

| **Plasma biomarkers** | **Cognitive function** | **β (95% CI)** | ***P-*value** |
| --- | --- | --- | --- |
| GFAP × age × time | Reaction time | -0.028 (-0.092, 0.036) | 0.394 |
| GFAP × age × time | Symbol digit substitution | -0.058 (-0.121, 0.004) | 0.066 |
| GFAP × age × time | Trail making | 0.022 (-0.049, 0.094) | 0.536 |
| GFAP × age × time | Pairs matching | 0.020 (-0.034, 0.075) | 0.465 |
| GFAP × age× time | Paired associate learning | -0.028 (-0.092, 0.036) | 0.394 |
| GFAP × age × time | Fluid intelligence | -0.058 (-0.121, 0.004) | 0.066 |
| NfL × age × time | Reaction time | 0.020 (-0.045, 0.084) | 0.556 |
| NfL × age × time | Symbol digit substitution | -0.073 (-0.136, -0.010) | **0.024** |
| **NfL × time in age < 65** | | **0.014 (-0.017, 0.046)** | **0.366** |
| **NfL × time in age ≥ 65** | | **-0.061 (-0.113, -0.009)** | **0.023** |
| NfL × age × time | Trail making | -0.015 (-0.086, 0.057) | 0.691 |
| NfL × age × time | Pairs matching | 0.019 (-0.071, 0.110) | 0.680 |
| NfL × age × time | Paired associate learning | 0.013 (-0.055, 0.080) | 0.712 |
| NfL × age× time | Fluid intelligence | 0.037 (-0.018, 0.093) | 0.188 |
| p-tau181 × age× time | Reaction time | 0.032 (-0.029, 0.093) | 0.309 |
| p-tau181 × age × time | Symbol digit substitution | -0.062 (-0.120, -0.003) | **0.041** |
| **p-tau181 × time in age < 65** | | **0.020 (-0.012, 0.052)** | **0.227** |
| **p-tau181× time in age ≥ 65** | | **-0.044 (-0.090, 0.003)** | **0.067** |
| p-tau181 × age × time | Trail making | -0.007 (-0.075, 0.060) | 0.832 |
| p-tau181 × age × time | Pairs matching | 0.027 (-0.025, 0.079) | 0.302 |
| p-tau181 × age× time | Paired associate learning | -0.034 (-0.090, 0.022) | 0.235 |
| p-tau181 × age × time | Fluid intelligence | 0.012 (-0.043, 0.067) | 0.670 |
| Aβ42/40 × age × time | Reaction time | 0.019 (-0.043, 0.081) | 0.553 |
| Aβ42/40 × age × time | Symbol digit substitution | 0.029 (-0.050, 0.107) | 0.473 |
| Aβ42/40 × age × time | Trail making | 0.019 (-0.039, 0.078) | 0.516 |
| Aβ42/40 × age × time | Pairs matching | -0.006 (-0.055, 0.042) | 0.797 |
| Aβ42/40 × age × time | Paired associate learning | 0.020 (-0.045, 0.084) | 0.556 |
| Aβ42/40 × age × time | Fluid intelligence | -0.073 (-0.136, -0.010) | 0.024 |

Abbreviations: Aβ, amyloid-β; GFAP, glial fibrillary acidic protein; NfL, neurofilament light chain; p-tau181, tau phosphorylated at threonine 181; CI, confidence interval.

Model was adjusted for sex, age, ethnicity, townsend deprivation index, assessment center, smoking status, alcohol use, *APOE* ε4 allele, hypertension, diabetes, cardiovascular arterial disease, and history of COVID-19 infection.

# Supplementary Table 4. Interactive associations of sex and elevated plasma AD-related biomarkers on cognitive decline.

| **Plasma biomarkers** | **Cognitive function** | **β (95% CI)** | ***P-*value** |
| --- | --- | --- | --- |
| GFAP × sex × time | Reaction time | 0.018 (-0.032, 0.069) | 0.484 |
| GFAP × sex × time | Symbol digit substitution | 0.037 (-0.012, 0.087) | 0.139 |
| GFAP × sex × time | Trail making | -0.012 (-0.065, 0.041) | 0.656 |
| GFAP × sex × time | Pairs matching | 0.018 (-0.031, 0.068) | 0.475 |
| GFAP × sex × time | Paired associate learning | -0.030 (-0.086, 0.027) | 0.301 |
| GFAP × sex × time | Fluid intelligence | 0.017 (-0.054, 0.088) | 0.647 |
| NfL × sex × time | Reaction time | 0.027 (-0.026, 0.079) | 0.325 |
| NfL × sex × time | Symbol digit substitution | 0.042 (-0.002, 0.085) | 0.060 |
| NfL × sex × time | Trail making | 0.005 (-0.050, 0.060) | 0.860 |
| NfL × sex × time | Pairs matching | 0.020 (-0.033, 0.073) | 0.462 |
| NfL × sex × time | Paired associate learning | 0.010 (-0.051, 0.071) | 0.753 |
| NfL × sex × time | Fluid intelligence | -0.021 (-0.098, 0.056) | 0.592 |
| p-tau181 × sex × time | Reaction time | -0.016 (-0.073, 0.041) | 0.583 |
| p-tau181 × sex × time | Symbol digit substitution | 0.002 (-0.045, 0.049) | 0.940 |
| p-tau181 × sex × time | Trail making | 0.001 (-0.047, 0.048) | 0.982 |
| p-tau181 × sex × time | Pairs matching | -0.027 (-0.074, 0.019) | 0.246 |
| p-tau181 × sex × time | Paired associate learning | 0.033 (-0.016, 0.082) | 0.187 |
| p-tau181 × sex × time | Fluid intelligence | 0.016 (-0.024, 0.057) | 0.430 |
| Aβ42/40 × sex × time | Reaction time | 0.018 (-0.032, 0.069) | 0.484 |
| Aβ42/40 × sex × time | Symbol digit substitution | 0.037 (-0.012, 0.087) | 0.139 |
| Aβ42/40 × sex × time | Trail making | -0.007 (-0.064, 0.050) | 0.805 |
| Aβ42/40 × sex × time | Pairs matching | -0.015 (-0.086, 0.056) | 0.674 |
| Aβ42/40 × sex × time | Paired associate learning | -0.012 (-0.065, 0.041) | 0.656 |
| Aβ42/40 × sex × time | Fluid intelligence | 0.033 (-0.010, 0.077) | 0.135 |

Abbreviations: Aβ, amyloid-β; GFAP, glial fibrillary acidic protein; NfL, neurofilament light chain; p-tau181, tau phosphorylated at threonine 181; CI, confidence interval.

Model was adjusted for sex, age, ethnicity, townsend deprivation index, assessment center, smoking status, alcohol use, *APOE ε4 allele*, hypertension, diabetes, cardiovascular arterial disease and history of COVID-19 infection.

# Supplementary Table 5. Interactive associations of COVID-19 and elevated plasma AD-related biomarkers on cognitive decline.

| Plasma biomarkers | Cognitive function | β (95% CI) | P-value |
| --- | --- | --- | --- |
| GFAP × COVID-19 × time | Reaction time | 0.019 (-0.031, 0.069) | 0.454 |
| GFAP × COVID-19 × time | Symbol digit substitution | -0.013 (-0.062, 0.035) | 0.588 |
| GFAP × COVID-19 × time | Trail making | 0.062 (0.006, 0.118) | **0.029** |
| **GFAP × time in COVID-19** | | -0.003 (-0.041, 0.034) | **0.866** |
| **GFAP × time in no COVID-19** | | -0.067 (-0.108, -0.026) | **0.002** |
| GFAP × COVID-19 × time | Pairs matching | -0.002 (-0.073, 0.068) | 0.946 |
| GFAP × COVID-19 × time | Paired associate learning | 0.015 (-0.037, 0.067) | 0.580 |
| GFAP × COVID-19 × time | Fluid intelligence | -0.021 (-0.064, 0.022) | 0.339 |
| NfL × COVID-19 × time | Reaction time | 0.043 (-0.008, 0.093) | 0.098 |
| NfL × COVID-19 × time | Symbol digit substitution | -0.047 (-0.097, 0.002) | 0.060 |
| NfL × COVID-19 × time | Trail making | 0.020 (-0.036, 0.076) | 0.484 |
| NfL × COVID-19 × time | Pairs matching | 0.028 (-0.043, 0.099) | 0.444 |
| NfL × COVID-19 × time | Paired associate learning | 0.012 (-0.041, 0.065) | 0.659 |
| NfL × COVID-19 × time | Fluid intelligence | 0.011 (-0.033, 0.054) | 0.633 |
| p-tau181 × COVID-19 × time | Reaction time | 0.019 (-0.035, 0.073) | 0.500 |
| p-tau181 × COVID-19 × time | Symbol digit substitution | -0.013 (-0.066, 0.039) | 0.616 |
| p-tau181 × COVID-19 × time | Trail making | 0.027 (-0.033, 0.087) | 0.379 |
| p-tau181 × COVID-19 × time | Pairs matching | -0.017 (-0.093, 0.058) | 0.653 |
| p-tau181 × COVID-19 × time | Paired associate learning | 0.000 (-0.056, 0.057) | 0.990 |
| p-tau181 × COVID-19 × time | Fluid intelligence | -0.045 (-0.091, 0.001) | 0.055 |
| Aβ42/40 × COVID-19 × time | Reaction time | 0.010 (-0.038, 0.057) | 0.690 |
| Aβ42/40 × COVID-19 × time | Symbol digit substitution | 0.003 (-0.043, 0.049) | 0.897 |
| Aβ42/40 × COVID-19 × time | Trail making | 0.010 (-0.043, 0.062) | 0.715 |
| Aβ42/40 × COVID-19 × time | Pairs matching | 0.015 (-0.051, 0.081) | 0.656 |
| Aβ42/40 × COVID-19 × time | Paired associate learning | -0.030 (-0.079, 0.019) | 0.229 |
| Aβ42/40 × COVID-19 × time | Fluid intelligence | 0.019 (-0.022, 0.059) | 0.359 |

Abbreviations: Aβ, amyloid-β; GFAP, glial fibrillary acidic protein; NfL, neurofilament light chain; p-tau181, tau phosphorylated at threonine 181; CI, confidence interval. Model was adjusted for sex, age, ethnicity, townsend deprivation index, assessment center, smoking status, alcohol use, APOE ε4 allele, hypertension, diabetes, cardiovascular arterial disease.

# Supplementary Table 6. Associations of longitudinal plasma AD-related biomarkers with longitudinal cognitive decline by using linear mixed-effects models.

| **Plasma biomarkers** | **Cognitive function** | **β (95% CI)** | ***P*-value** | **FDR-**  **corrected *P*** |
| --- | --- | --- | --- | --- |
| Aβ42/40 | Reaction time | -0.006 (-0.031, 0.018) | 0.628 | 0.843 |
| Aβ42/40 | Symbol digit substitution | -0.007 (-0.031, 0.017) | 0.593 | 0.843 |
| Aβ42/40 | Trail making | 0.001 (-0.026, 0.029) | 0.927 | 0.959 |
| Aβ42/40 | Pairs matching | -0.003 (-0.038, 0.031) | 0.854 | 0.932 |
| Aβ42/40 | Paired associate learning | -0.006 (-0.031, 0.019) | 0.643 | 0.843 |
| Aβ42/40 | Fluid intelligence | 0.006 (-0.015, 0.027) | 0.559 | 0.843 |
| GFAP | Reaction time | 0.007 (-0.018, 0.033) | 0.576 | 0.843 |
| GFAP | Symbol digit substitution | -0.041 (-0.066, -0.016) | 0.002 | 0.019 |
| GFAP | Trail making | -0.048 (-0.076, -0.019) | 0.001 | 0.019 |
| GFAP | Pairs matching | -0.018 (-0.055, 0.018) | 0.316 | 0.843 |
| GFAP | Paired associate learning | -0.026 (-0.053, 0.001) | 0.056 | 0.334 |
| GFAP | Fluid intelligence | 0.010 (-0.012, 0.032) | 0.383 | 0.843 |
| NFL | Reaction time | -0.005 (-0.031, 0.020) | 0.681 | 0.843 |
| NFL | Symbol digit substitution | -0.010 (-0.036, 0.015) | 0.417 | 0.843 |
| NFL | Trail making | -0.012 (-0.041, 0.017) | 0.409 | 0.843 |
| NFL | Pairs matching | -0.030 (-0.066, 0.006) | 0.098 | 0.393 |
| NFL | Paired associate learning | -0.007 (-0.034, 0.020) | 0.615 | 0.843 |
| NFL | Fluid intelligence | 0.013 (-0.010, 0.035) | 0.266 | 0.843 |
| p-tau181 | Reaction time | 0.005 (-0.020, 0.030) | 0.702 | 0.843 |
| p-tau181 | Symbol digit substitution | -0.020 (-0.044, 0.003) | 0.095 | 0.393 |
| p-tau181 | Trail making | -0.037 (-0.064, -0.009) | 0.008 | 0.068 |
| p-tau181 | Pairs matching | -0.005 (-0.039, 0.030) | 0.786 | 0.899 |
| p-tau181 | Paired associate learning | 0.001 (-0.025, 0.026) | 0.959 | 0.959 |
| p-tau181 | Fluid intelligence | -0.006 (-0.027, 0.016) | 0.609 | 0.843 |

Abbreviations: Aβ, amyloid-β; GFAP, glial fibrillary acidic protein; NfL, neurofilament light chain; p-tau181, tau phosphorylated at threonine 181; CI, confidence interval; FDR, false discovery rates.

Model was adjusted for sex, age, ethnicity, townsend deprivation index, assessment center, smoking status, alcohol use, family history of dementia, *APOE* ε4 allele, hypertension, diabetes, cardiovascular arterial disease, and history of COVID-19 infection.

# Supplementary Table 7. Associations of baseline plasma AD-related biomarkers with longitudinal changes in total brain structure by using linear mixed-effects models.

| **Plasma biomarkers** | **Total brain structure** | **β (95% CI)** | ***P*-value** | **FDR-**  **corrected *P*** |
| --- | --- | --- | --- | --- |
| Aβ42/40 | Grey matter volume | -0.001 (-0.008, 0.005) | 0.691 | 0.806 |
| Aβ42/40 | White matter volume | 0.009 (0.001, 0.017) | 0.033 | 0.102 |
| Aβ42/40 | Area of total surface (l) | 0.002 (0.000, 0.004) | 0.050 | 0.140 |
| Aβ42/40 | Mean thickness (l) | 0.009 (-0.005, 0.023) | 0.196 | 0.340 |
| Aβ42/40 | Area of total surface (r) | 0.003 (0.001, 0.005) | 0.015 | 0.061 |
| Aβ42/40 | Mean thickness (r) | 0.011 (-0.003, 0.025) | 0.118 | 0.221 |
| GFAP | Grey matter volume | -0.011 (-0.017, -0.005) | <0.001 | 0.003 |
| GFAP | White matter volume | -0.007 (-0.015, 0.001) | 0.079 | 0.185 |
| GFAP | Area of total surface (l) | -0.003 (-0.005, -0.001) | 0.006 | 0.043 |
| GFAP | Mean thickness (l) | -0.008 (-0.021, 0.005) | 0.207 | 0.340 |
| GFAP | Area of total surface (r) | -0.003 (-0.005, 0.000) | 0.021 | 0.073 |
| GFAP | Mean thickness (r) | -0.011 (-0.024, 0.002) | 0.108 | 0.216 |
| NFL | Grey matter volume | -0.003 (-0.009, 0.003) | 0.335 | 0.469 |
| NFL | White matter volume | -0.010 (-0.018, -0.003) | 0.008 | 0.043 |
| NFL | Area of total surface (l) | -0.004 (-0.006, -0.002) | <0.001 | 0.003 |
| NFL | Mean thickness (l) | -0.007 (-0.020, 0.005) | 0.267 | 0.415 |
| NFL | Area of total surface (r) | -0.003 (-0.006, -0.001) | 0.002 | 0.017 |
| NFL | Mean thickness (r) | -0.006 (-0.019, 0.007) | 0.354 | 0.472 |
| p-tau181 | Grey matter volume | -0.001 (-0.007, 0.005) | 0.738 | 0.827 |
| p-tau181 | White matter volume | 0.000 (-0.008, 0.007) | 0.937 | 0.954 |
| p-tau181 | Area of total surface (l) | -0.001 (-0.003, 0.001) | 0.411 | 0.501 |
| p-tau181 | Mean thickness (l) | -0.012 (-0.025, 0.001) | 0.063 | 0.161 |
| p-tau181 | Area of total surface (r) | 0.000 (-0.002, 0.002) | 0.769 | 0.829 |
| p-tau181 | Mean thickness (r) | -0.011 (-0.024, 0.002) | 0.089 | 0.191 |

Abbreviations: Aβ, amyloid-β; GFAP, glial fibrillary acidic protein; NfL, neurofilament light chain; p-tau181, tau phosphorylated at threonine 181; CI, confidence interval; l, left hemisphere; r, right hemisphere; FDR, false discovery rates.

Model was adjusted for sex, age, ethnicity, townsend deprivation index, assessment center, smoking status, alcohol use, family history of dementia, *APOE* ε4 allele, hypertension, diabetes, cardiovascular arterial disease, history of COVID-19 infection, and total intracranial volume.

# Supplementary Table 8. Associations of longitudinal plasma AD-related biomarkers with longitudinal changes in total brain structure by using linear mixed-effects models.

| **Plasma biomarkers** | **Total brain structure** | **β (95% CI)** | ***P*-value** | **FDR-**  **corrected *P*** |
| --- | --- | --- | --- | --- |
| Aβ42/40 | Grey matter volume | 0.000 (-0.008, 0.008) | 0.967 | 0.967 |
| Aβ42/40 | White matter volume | -0.010 (-0.021, 0.000) | 0.054 | 0.319 |
| Aβ42/40 | Area of total surface (l) | -0.002 (-0.005, 0.001) | 0.172 | 0.535 |
| Aβ42/40 | Mean thickness (l) | -0.013 (-0.031, 0.004) | 0.143 | 0.532 |
| Aβ42/40 | Area of total surface (r) | -0.004 (-0.007, -0.001) | 0.016 | 0.224 |
| Aβ42/40 | Mean thickness (r) | -0.011 (-0.029, 0.007) | 0.251 | 0.702 |
| GFAP | Grey matter volume | 0.001 (-0.007, 0.009) | 0.760 | 0.967 |
| GFAP | White matter volume | 0.000 (-0.011, 0.010) | 0.935 | 0.967 |
| GFAP | Area of total surface (l) | 0.000 (-0.002, 0.003) | 0.805 | 0.967 |
| GFAP | Mean thickness (l) | 0.008 (-0.010, 0.026) | 0.366 | 0.731 |
| GFAP | Area of total surface (r) | 0.000 (-0.003, 0.003) | 0.855 | 0.967 |
| GFAP | Mean thickness (r) | -0.018 (-0.036, 0.001) | 0.057 | 0.319 |
| NFL | Grey matter volume | -0.002 (-0.009, 0.004) | 0.492 | 0.861 |
| NFL | White matter volume | 0.000 (-0.009, 0.009) | 0.936 | 0.967 |
| NFL | Area of total surface (l) | -0.001 (-0.004, 0.001) | 0.363 | 0.731 |
| NFL | Mean thickness (l) | 0.001 (-0.014, 0.016) | 0.875 | 0.967 |
| NFL | Area of total surface (r) | -0.002 (-0.005, 0.000) | 0.078 | 0.364 |
| NFL | Mean thickness (r) | 0.002 (-0.013, 0.017) | 0.807 | 0.967 |
| p-tau181 | Grey matter volume | -0.002 (-0.010, 0.005) | 0.555 | 0.913 |
| p-tau181 | White matter volume | -0.004 (-0.014, 0.007) | 0.468 | 0.861 |
| p-tau181 | Area of total surface (l) | 0.000 (-0.002, 0.003) | 0.801 | 0.967 |
| p-tau181 | Mean thickness (l) | -0.004 (-0.021, 0.014) | 0.667 | 0.967 |
| p-tau181 | Area of total surface (r) | 0.000 (-0.003, 0.003) | 0.832 | 0.967 |
| p-tau181 | Mean thickness (r) | 0.008 (-0.009, 0.026) | 0.366 | 0.731 |

Abbreviations: Aβ, amyloid-β; GFAP, glial fibrillary acidic protein; NfL, neurofilament light chain; p-tau181, tau phosphorylated at threonine 181; CI, confidence interval; l, left hemisphere; r, right hemisphere; FDR, false discovery rates.

Model was adjusted for sex, age, ethnicity, townsend deprivation index, assessment center, smoking status, alcohol use, family history of dementia, *APOE* ε4 allele, hypertension, diabetes, cardiovascular arterial disease, history of COVID-19 infection, and total intracranial volume.

# Supplementary Table 9. Associations of baseline plasma AD-related biomarkers with longitudinal changes in cortical gray volumes by using linear mixed-effects models.

| **Plasma biomarkers** | **Cortical gray volumes** | **β (95% CI)** | ***P*-value** | **FDR-**  **corrected *P*** |
| --- | --- | --- | --- | --- |
| Aβ42/40 | (l) bankssts volume | -0.001 (-0.007, 0.005) | 0.832 | 0.872 |
| Aβ42/40 | (l) caudalanteriorcingulate volume | 0.001 (-0.009, 0.010) | 0.882 | 0.910 |
| Aβ42/40 | (l) caudalmiddlefrontal volume | -0.001 (-0.007, 0.005) | 0.740 | 0.801 |
| Aβ42/40 | (l) cuneus volume | 0.004 (-0.003, 0.011) | 0.306 | 0.531 |
| Aβ42/40 | (l) entorhinal volume | 0.006 (-0.006, 0.018) | 0.345 | 0.542 |
| Aβ42/40 | (l) frontalpole volume | 0.015 (0.001, 0.029) | 0.037 | 0.244 |
| Aβ42/40 | (l) fusiform volume | 0.007 (0.001, 0.014) | 0.033 | 0.244 |
| Aβ42/40 | (l) inferiorparietal volume | 0.003 (-0.002, 0.008) | 0.285 | 0.512 |
| Aβ42/40 | (l) inferiortemporal volume | 0.005 (0.000, 0.011) | 0.055 | 0.278 |
| Aβ42/40 | (l) insula volume | 0.005 (-0.005, 0.015) | 0.353 | 0.542 |
| Aβ42/40 | (l) isthmuscingulate volume | 0.003 (-0.003, 0.009) | 0.340 | 0.542 |
| Aβ42/40 | (l) lateraloccipital volume | 0.006 (-0.001, 0.013) | 0.097 | 0.356 |
| Aβ42/40 | (l) lateralorbitofrontal volume | 0.005 (-0.002, 0.012) | 0.189 | 0.450 |
| Aβ42/40 | (l) lingual volume | 0.006 (-0.002, 0.014) | 0.122 | 0.383 |
| Aβ42/40 | (l) medialorbitofrontal volume | 0.004 (-0.007, 0.016) | 0.482 | 0.636 |
| Aβ42/40 | (l) middletemporal volume | 0.002 (-0.005, 0.009) | 0.552 | 0.675 |
| Aβ42/40 | (l) paracentral volume | 0.006 (-0.003, 0.015) | 0.204 | 0.454 |
| Aβ42/40 | (l) parahippocampal volume | 0.008 (-0.002, 0.018) | 0.139 | 0.413 |
| Aβ42/40 | (l) parsopercularis volume | -0.003 (-0.009, 0.003) | 0.315 | 0.533 |
| Aβ42/40 | (l) parsorbitalis volume | 0.010 (0.001, 0.019) | 0.023 | 0.244 |
| Aβ42/40 | (l) parstriangularis volume | 0.002 (-0.004, 0.007) | 0.527 | 0.656 |
| Aβ42/40 | (l) pericalcarine volume | 0.001 (-0.005, 0.008) | 0.720 | 0.795 |
| Aβ42/40 | (l) postcentral volume | 0.003 (-0.005, 0.010) | 0.453 | 0.623 |
| Aβ42/40 | (l) posteriorcingulate volume | 0.008 (0.001, 0.016) | 0.029 | 0.244 |
| Aβ42/40 | (l) precentral volume | 0.005 (-0.004, 0.013) | 0.255 | 0.481 |
| Aβ42/40 | (l) precuneus volume | 0.007 (0.001, 0.013) | 0.031 | 0.244 |
| Aβ42/40 | (l) rostralanteriorcingulate volume | 0.000 (-0.007, 0.006) | 0.913 | 0.924 |
| Aβ42/40 | (l) rostralmiddlefrontal volume | -0.001 (-0.007, 0.005) | 0.699 | 0.795 |
| Aβ42/40 | (l) superiorfrontal volume | 0.004 (-0.003, 0.010) | 0.287 | 0.512 |
| Aβ42/40 | (l) superiorparietal volume | 0.007 (0.000, 0.014) | 0.042 | 0.252 |
| Aβ42/40 | (l) superiortemporal volume | 0.007 (0.001, 0.014) | 0.027 | 0.244 |
| Aβ42/40 | (l) supramarginal volume | 0.005 (0.000, 0.010) | 0.059 | 0.278 |
| Aβ42/40 | (l) transversetemporal volume | -0.001 (-0.008, 0.006) | 0.816 | 0.869 |
| Aβ42/40 | (r) bankssts volume | 0.003 (-0.004, 0.009) | 0.421 | 0.604 |
| Aβ42/40 | (r) caudalanteriorcingulate volume | 0.009 (0.001, 0.018) | 0.036 | 0.244 |
| Aβ42/40 | (r) caudalmiddlefronta volume | 0.004 (-0.003, 0.011) | 0.220 | 0.454 |
| Aβ42/40 | (r) cuneus volume | 0.004 (-0.004, 0.011) | 0.346 | 0.542 |
| Aβ42/40 | (r) entorhinal volume | -0.005 (-0.017, 0.008) | 0.468 | 0.630 |
| Aβ42/40 | (r) frontalpole volume | 0.006 (-0.010, 0.022) | 0.448 | 0.623 |
| Aβ42/40 | (r) fusiform volume | 0.007 (0.000, 0.013) | 0.037 | 0.244 |
| Aβ42/40 | (r) inferiorparietal volume | 0.005 (-0.001, 0.010) | 0.094 | 0.356 |
| Aβ42/40 | (r) inferiortemporal volume | 0.001 (-0.004, 0.006) | 0.718 | 0.795 |
| Aβ42/40 | (r) insula volume | 0.007 (-0.004, 0.018) | 0.222 | 0.454 |
| Aβ42/40 | (r) isthmuscingulate volume | 0.004 (-0.003, 0.011) | 0.234 | 0.454 |
| Aβ42/40 | (r) lateraloccipital volume | 0.002 (-0.005, 0.009) | 0.505 | 0.641 |
| Aβ42/40 | (r) lateralorbitofrontal volume | 0.002 (-0.008, 0.012) | 0.723 | 0.795 |
| Aβ42/40 | (r) lingual volume | 0.005 (-0.003, 0.013) | 0.230 | 0.454 |
| Aβ42/40 | (r) medialorbitofrontal volume | 0.000 (-0.010, 0.009) | 0.924 | 0.924 |
| Aβ42/40 | (r) middletemporal volume | 0.006 (0.000, 0.012) | 0.059 | 0.278 |
| Aβ42/40 | (r) paracentral volume | 0.007 (-0.001, 0.016) | 0.105 | 0.365 |
| Aβ42/40 | (r) parahippocampal volume | 0.010 (-0.001, 0.022) | 0.075 | 0.309 |
| Aβ42/40 | (r) parsopercularis volume | 0.005 (-0.001, 0.011) | 0.117 | 0.383 |
| Aβ42/40 | (r) parsorbitalis volume | 0.002 (-0.006, 0.011) | 0.586 | 0.703 |
| Aβ42/40 | (r) parstriangularis volume | 0.004 (-0.002, 0.010) | 0.178 | 0.450 |
| Aβ42/40 | (r) pericalcarine volume | 0.002 (-0.006, 0.009) | 0.628 | 0.740 |
| Aβ42/40 | (r) postcentral volume | 0.006 (-0.002, 0.014) | 0.148 | 0.413 |
| Aβ42/40 | (r) posteriorcingulate volume | 0.010 (0.001, 0.018) | 0.022 | 0.244 |
| Aβ42/40 | (r) precentral volume | 0.006 (-0.003, 0.015) | 0.191 | 0.450 |
| Aβ42/40 | (r) precuneus volume | 0.006 (0.000, 0.012) | 0.072 | 0.309 |
| Aβ42/40 | (r) rostralanteriorcingulate volume | 0.003 (-0.004, 0.010) | 0.395 | 0.593 |
| Aβ42/40 | (r) rostralmiddlefrontal volume | 0.006 (0.000, 0.012) | 0.034 | 0.244 |
| Aβ42/40 | (r) superiorfrontal volume | 0.005 (-0.002, 0.012) | 0.150 | 0.413 |
| Aβ42/40 | (r) superiorparietal volume | 0.003 (-0.006, 0.011) | 0.497 | 0.641 |
| Aβ42/40 | (r) superiortemporal volume | 0.005 (-0.002, 0.011) | 0.166 | 0.438 |
| Aβ42/40 | (r) supramarginal volume | 0.004 (-0.002, 0.011) | 0.217 | 0.454 |
| Aβ42/40 | (r) transversetemporal volume | 0.004 (-0.005, 0.013) | 0.416 | 0.604 |
| GFAP | (l) bankssts volume | -0.001 (-0.007, 0.004) | 0.640 | 0.741 |
| GFAP | (l) caudalanteriorcingulate volume | 0.007 (-0.002, 0.015) | 0.131 | 0.234 |
| GFAP | (l) caudalmiddlefrontal volume | -0.004 (-0.010, 0.001) | 0.125 | 0.234 |
| GFAP | (l) cuneus volume | -0.006 (-0.012, 0.001) | 0.107 | 0.228 |
| GFAP | (l) entorhinal volume | 0.005 (-0.006, 0.017) | 0.365 | 0.502 |
| GFAP | (l) frontalpole volume | -0.003 (-0.016, 0.011) | 0.680 | 0.750 |
| GFAP | (l) fusiform volume | -0.012 (-0.019, -0.006) | <0.001 | <0.001 |
| GFAP | (l) inferiorparietal volume | -0.004 (-0.009, 0.001) | 0.089 | 0.200 |
| GFAP | (l) inferiortemporal volume | -0.005 (-0.010, 0.000) | 0.054 | 0.187 |
| GFAP | (l) insula volume | 0.001 (-0.009, 0.010) | 0.906 | 0.906 |
| GFAP | (l) isthmuscingulate volume | -0.001 (-0.007, 0.005) | 0.682 | 0.750 |
| GFAP | (l) lateraloccipital volume | -0.006 (-0.013, 0.001) | 0.085 | 0.200 |
| GFAP | (l) lateralorbitofrontal volume | -0.006 (-0.013, 0.000) | 0.065 | 0.187 |
| GFAP | (l) lingual volume | -0.005 (-0.012, 0.002) | 0.170 | 0.281 |
| GFAP | (l) medialorbitofrontal volume | -0.008 (-0.019, 0.002) | 0.127 | 0.234 |
| GFAP | (l) middletemporal volume | -0.005 (-0.011, 0.001) | 0.126 | 0.234 |
| GFAP | (l) paracentral volume | -0.002 (-0.011, 0.006) | 0.581 | 0.724 |
| GFAP | (l) parahippocampal volume | -0.003 (-0.012, 0.007) | 0.594 | 0.726 |
| GFAP | (l) parsopercularis volume | -0.004 (-0.009, 0.001) | 0.138 | 0.234 |
| GFAP | (l) parsorbitalis volume | -0.013 (-0.021, -0.005) | 0.002 | 0.033 |
| GFAP | (l) parstriangularis volume | -0.003 (-0.008, 0.002) | 0.203 | 0.312 |
| GFAP | (l) pericalcarine volume | -0.003 (-0.009, 0.003) | 0.335 | 0.481 |
| GFAP | (l) postcentral volume | -0.005 (-0.012, 0.002) | 0.127 | 0.234 |
| GFAP | (l) posteriorcingulate volume | 0.003 (-0.005, 0.010) | 0.473 | 0.624 |
| GFAP | (l) precentral volume | -0.007 (-0.014, 0.001) | 0.091 | 0.200 |
| GFAP | (l) precuneus volume | -0.006 (-0.011, 0.000) | 0.036 | 0.158 |
| GFAP | (l) rostralanteriorcingulate volume | 0.004 (-0.002, 0.010) | 0.226 | 0.339 |
| GFAP | (l) rostralmiddlefrontal volume | -0.005 (-0.010, 0.000) | 0.065 | 0.187 |
| GFAP | (l) superiorfrontal volume | -0.009 (-0.015, -0.003) | 0.003 | 0.033 |
| GFAP | (l) superiorparietal volume | -0.006 (-0.012, 0.000) | 0.062 | 0.187 |
| GFAP | (l) superiortemporal volume | -0.008 (-0.014, -0.002) | 0.010 | 0.094 |
| GFAP | (l) supramarginal volume | -0.004 (-0.009, 0.000) | 0.057 | 0.187 |
| GFAP | (l) transversetemporal volume | -0.007 (-0.013, 0.000) | 0.035 | 0.158 |
| GFAP | (r) bankssts volume | -0.004 (-0.010, 0.002) | 0.231 | 0.339 |
| GFAP | (r) caudalanteriorcingulate volume | -0.002 (-0.010, 0.006) | 0.653 | 0.743 |
| GFAP | (r) caudalmiddlefronta volume | -0.010 (-0.016, -0.004) | 0.002 | 0.033 |
| GFAP | (r) cuneus volume | -0.005 (-0.012, 0.002) | 0.178 | 0.287 |
| GFAP | (r) entorhinal volume | -0.015 (-0.027, -0.003) | 0.015 | 0.110 |
| GFAP | (r) frontalpole volume | 0.005 (-0.010, 0.020) | 0.487 | 0.630 |
| GFAP | (r) fusiform volume | -0.010 (-0.016, -0.003) | 0.002 | 0.033 |
| GFAP | (r) inferiorparietal volume | -0.004 (-0.009, 0.001) | 0.135 | 0.234 |
| GFAP | (r) inferiortemporal volume | -0.006 (-0.011, -0.001) | 0.012 | 0.099 |
| GFAP | (r) insula volume | 0.001 (-0.010, 0.011) | 0.903 | 0.906 |
| GFAP | (r) isthmuscingulate volume | -0.006 (-0.013, 0.000) | 0.053 | 0.187 |
| GFAP | (r) lateraloccipital volume | -0.005 (-0.012, 0.002) | 0.138 | 0.234 |
| GFAP | (r) lateralorbitofrontal volume | -0.008 (-0.018, 0.001) | 0.082 | 0.200 |
| GFAP | (r) lingual volume | -0.007 (-0.014, 0.001) | 0.082 | 0.200 |
| GFAP | (r) medialorbitofrontal volume | -0.002 (-0.011, 0.007) | 0.622 | 0.733 |
| GFAP | (r) middletemporal volume | -0.007 (-0.012, -0.001) | 0.027 | 0.149 |
| GFAP | (r) paracentral volume | -0.001 (-0.009, 0.008) | 0.893 | 0.906 |
| GFAP | (r) parahippocampal volume | -0.004 (-0.015, 0.007) | 0.461 | 0.621 |
| GFAP | (r) parsopercularis volume | -0.006 (-0.011, 0.000) | 0.061 | 0.187 |
| GFAP | (r) parsorbitalis volume | -0.003 (-0.011, 0.005) | 0.516 | 0.655 |
| GFAP | (r) parstriangularis volume | -0.006 (-0.012, 0.000) | 0.035 | 0.158 |
| GFAP | (r) pericalcarine volume | -0.001 (-0.008, 0.006) | 0.820 | 0.859 |
| GFAP | (r) postcentral volume | -0.007 (-0.014, 0.001) | 0.080 | 0.200 |
| GFAP | (r) posteriorcingulate volume | -0.009 (-0.017, -0.001) | 0.026 | 0.149 |
| GFAP | (r) precentral volume | -0.008 (-0.017, 0.000) | 0.045 | 0.186 |
| GFAP | (r) precuneus volume | -0.007 (-0.012, -0.001) | 0.021 | 0.139 |
| GFAP | (r) rostralanteriorcingulate volume | -0.003 (-0.009, 0.003) | 0.344 | 0.483 |
| GFAP | (r) rostralmiddlefrontal volume | -0.005 (-0.010, 0.001) | 0.086 | 0.200 |
| GFAP | (r) superiorfrontal volume | -0.009 (-0.015, -0.003) | 0.003 | 0.033 |
| GFAP | (r) superiorparietal volume | -0.001 (-0.009, 0.007) | 0.757 | 0.819 |
| GFAP | (r) superiortemporal volume | -0.004 (-0.010, 0.002) | 0.200 | 0.312 |
| GFAP | (r) supramarginal volume | -0.001 (-0.007, 0.005) | 0.791 | 0.842 |
| GFAP | (r) transversetemporal volume | 0.002 (-0.006, 0.011) | 0.615 | 0.733 |
| NfL | (l) bankssts volume | -0.002 (-0.007, 0.004) | 0.517 | 0.685 |
| NfL | (l) caudalanteriorcingulate volume | 0.003 (-0.006, 0.011) | 0.497 | 0.685 |
| NfL | (l) caudalmiddlefrontal volume | -0.005 (-0.011, 0.000) | 0.060 | 0.293 |
| NfL | (l) cuneus volume | -0.008 (-0.015, -0.002) | 0.012 | 0.113 |
| NfL | (l) entorhinal volume | 0.003 (-0.008, 0.014) | 0.558 | 0.708 |
| NfL | (l) frontalpole volume | 0.002 (-0.011, 0.015) | 0.785 | 0.831 |
| NfL | (l) fusiform volume | -0.011 (-0.017, -0.005) | <0.001 | <0.001 |
| NfL | (l) inferiorparietal volume | -0.003 (-0.007, 0.002) | 0.242 | 0.537 |
| NfL | (l) inferiortemporal volume | -0.005 (-0.010, 0.000) | 0.035 | 0.257 |
| NfL | (l) insula volume | 0.001 (-0.007, 0.010) | 0.746 | 0.827 |
| NfL | (l) isthmuscingulate volume | -0.001 (-0.007, 0.004) | 0.639 | 0.791 |
| NfL | (l) lateraloccipital volume | -0.009 (-0.015, -0.002) | 0.012 | 0.113 |
| NfL | (l) lateralorbitofrontal volume | -0.002 (-0.008, 0.004) | 0.523 | 0.685 |
| NfL | (l) lingual volume | -0.008 (-0.015, -0.001) | 0.031 | 0.256 |
| NfL | (l) medialorbitofrontal volume | -0.004 (-0.015, 0.006) | 0.433 | 0.666 |
| NfL | (l) middletemporal volume | -0.005 (-0.011, 0.001) | 0.114 | 0.371 |
| NfL | (l) paracentral volume | -0.005 (-0.013, 0.003) | 0.244 | 0.537 |
| NfL | (l) parahippocampal volume | -0.006 (-0.015, 0.004) | 0.229 | 0.537 |
| NfL | (l) parsopercularis volume | -0.010 (-0.015, -0.004) | <0.001 | <0.001 |
| NfL | (l) parsorbitalis volume | -0.008 (-0.016, 0.000) | 0.047 | 0.293 |
| NfL | (l) parstriangularis volume | -0.003 (-0.008, 0.002) | 0.278 | 0.592 |
| NfL | (l) pericalcarine volume | -0.002 (-0.009, 0.004) | 0.420 | 0.666 |
| NfL | (l) postcentral volume | -0.006 (-0.012, 0.001) | 0.110 | 0.371 |
| NfL | (l) posteriorcingulate volume | -0.003 (-0.010, 0.003) | 0.331 | 0.620 |
| NfL | (l) precentral volume | 0.002 (-0.006, 0.009) | 0.691 | 0.791 |
| NfL | (l) precuneus volume | -0.005 (-0.010, 0.001) | 0.091 | 0.353 |
| NfL | (l) rostralanteriorcingulate volume | -0.001 (-0.007, 0.006) | 0.830 | 0.856 |
| NfL | (l) rostralmiddlefrontal volume | -0.009 (-0.014, -0.004) | 0.001 | 0.017 |
| NfL | (l) superiorfrontal volume | -0.011 (-0.017, -0.005) | <0.001 | <0.001 |
| NfL | (l) superiorparietal volume | -0.002 (-0.009, 0.004) | 0.503 | 0.685 |
| NfL | (l) superiortemporal volume | -0.004 (-0.009, 0.002) | 0.223 | 0.537 |
| NfL | (l) supramarginal volume | 0.000 (-0.004, 0.005) | 0.956 | 0.956 |
| NfL | (l) transversetemporal volume | -0.002 (-0.008, 0.004) | 0.474 | 0.685 |
| NfL | (r) bankssts volume | -0.003 (-0.009, 0.003) | 0.320 | 0.620 |
| NfL | (r) caudalanteriorcingulate volume | -0.003 (-0.011, 0.005) | 0.507 | 0.685 |
| NfL | (r) caudalmiddlefronta volume | -0.006 (-0.012, 0.000) | 0.064 | 0.293 |
| NfL | (r) cuneus volume | -0.003 (-0.010, 0.003) | 0.337 | 0.620 |
| NfL | (r) entorhinal volume | -0.006 (-0.017, 0.006) | 0.319 | 0.620 |
| NfL | (r) frontalpole volume | 0.002 (-0.012, 0.016) | 0.793 | 0.831 |
| NfL | (r) fusiform volume | -0.005 (-0.011, 0.001) | 0.118 | 0.371 |
| NfL | (r) inferiorparietal volume | -0.001 (-0.006, 0.004) | 0.673 | 0.791 |
| NfL | (r) inferiortemporal volume | -0.005 (-0.009, 0.000) | 0.057 | 0.293 |
| NfL | (r) insula volume | -0.005 (-0.015, 0.006) | 0.385 | 0.666 |
| NfL | (r) isthmuscingulate volume | -0.005 (-0.011, 0.001) | 0.134 | 0.385 |
| NfL | (r) lateraloccipital volume | -0.001 (-0.008, 0.005) | 0.672 | 0.791 |
| NfL | (r) lateralorbitofrontal volume | 0.002 (-0.007, 0.011) | 0.671 | 0.791 |
| NfL | (r) lingual volume | -0.007 (-0.014, 0.001) | 0.071 | 0.293 |
| NfL | (r) medialorbitofrontal volume | 0.001 (-0.007, 0.010) | 0.752 | 0.827 |
| NfL | (r) middletemporal volume | -0.004 (-0.009, 0.002) | 0.196 | 0.498 |
| NfL | (r) paracentral volume | 0.000 (-0.008, 0.008) | 0.927 | 0.941 |
| NfL | (r) parahippocampal volume | 0.004 (-0.006, 0.015) | 0.400 | 0.666 |
| NfL | (r) parsopercularis volume | -0.004 (-0.010, 0.001) | 0.127 | 0.381 |
| NfL | (r) parsorbitalis volume | 0.004 (-0.004, 0.012) | 0.338 | 0.620 |
| NfL | (r) parstriangularis volume | -0.002 (-0.008, 0.003) | 0.391 | 0.666 |
| NfL | (r) pericalcarine volume | -0.002 (-0.009, 0.005) | 0.529 | 0.685 |
| NfL | (r) postcentral volume | -0.005 (-0.012, 0.002) | 0.165 | 0.436 |
| NfL | (r) posteriorcingulate volume | -0.011 (-0.019, -0.003) | 0.004 | 0.053 |
| NfL | (r) precentral volume | -0.003 (-0.011, 0.005) | 0.464 | 0.685 |
| NfL | (r) precuneus volume | -0.005 (-0.011, 0.000) | 0.054 | 0.293 |
| NfL | (r) rostralanteriorcingulate volume | -0.006 (-0.012, 0.000) | 0.067 | 0.293 |
| NfL | (r) rostralmiddlefrontal volume | -0.002 (-0.008, 0.003) | 0.434 | 0.666 |
| NfL | (r) superiorfrontal volume | -0.004 (-0.010, 0.002) | 0.163 | 0.436 |
| NfL | (r) superiorparietal volume | -0.003 (-0.011, 0.004) | 0.412 | 0.666 |
| NfL | (r) superiortemporal volume | -0.001 (-0.007, 0.005) | 0.695 | 0.791 |
| NfL | (r) supramarginal volume | -0.001 (-0.007, 0.005) | 0.771 | 0.831 |
| NfL | (r) transversetemporal volume | 0.007 (-0.001, 0.015) | 0.098 | 0.359 |
| p-tau181 | (l) bankssts volume | -0.001 (-0.007, 0.004) | 0.691 | 0.845 |
| p-tau181 | (l) caudalanteriorcingulate volume | 0.005 (-0.004, 0.013) | 0.270 | 0.470 |
| p-tau181 | (l) caudalmiddlefrontal volume | -0.007 (-0.012, -0.001) | 0.018 | 0.108 |
| p-tau181 | (l) cuneus volume | -0.004 (-0.011, 0.002) | 0.203 | 0.460 |
| p-tau181 | (l) entorhinal volume | -0.007 (-0.018, 0.005) | 0.245 | 0.470 |
| p-tau181 | (l) frontalpole volume | -0.016 (-0.029, -0.003) | 0.015 | 0.108 |
| p-tau181 | (l) fusiform volume | -0.006 (-0.012, 0.000) | 0.063 | 0.219 |
| p-tau181 | (l) inferiorparietal volume | -0.007 (-0.011, -0.002) | 0.006 | 0.099 |
| p-tau181 | (l) inferiortemporal volume | -0.003 (-0.008, 0.002) | 0.209 | 0.460 |
| p-tau181 | (l) insula volume | -0.006 (-0.015, 0.003) | 0.177 | 0.433 |
| p-tau181 | (l) isthmuscingulate volume | -0.003 (-0.008, 0.003) | 0.341 | 0.512 |
| p-tau181 | (l) lateraloccipital volume | -0.007 (-0.014, -0.001) | 0.035 | 0.178 |
| p-tau181 | (l) lateralorbitofrontal volume | 0.000 (-0.006, 0.007) | 0.969 | 0.990 |
| p-tau181 | (l) lingual volume | -0.006 (-0.013, 0.001) | 0.097 | 0.278 |
| p-tau181 | (l) medialorbitofrontal volume | 0.000 (-0.010, 0.011) | 0.942 | 0.990 |
| p-tau181 | (l) middletemporal volume | -0.005 (-0.011, 0.001) | 0.125 | 0.344 |
| p-tau181 | (l) paracentral volume | -0.007 (-0.015, 0.001) | 0.085 | 0.255 |
| p-tau181 | (l) parahippocampal volume | -0.006 (-0.015, 0.003) | 0.205 | 0.460 |
| p-tau181 | (l) parsopercularis volume | -0.006 (-0.011, -0.001) | 0.017 | 0.108 |
| p-tau181 | (l) parsorbitalis volume | -0.008 (-0.016, 0.000) | 0.052 | 0.202 |
| p-tau181 | (l) parstriangularis volume | -0.003 (-0.008, 0.002) | 0.278 | 0.470 |
| p-tau181 | (l) pericalcarine volume | -0.002 (-0.009, 0.004) | 0.423 | 0.570 |
| p-tau181 | (l) postcentral volume | -0.008 (-0.015, -0.001) | 0.018 | 0.108 |
| p-tau181 | (l) posteriorcingulate volume | -0.001 (-0.008, 0.006) | 0.861 | 0.990 |
| p-tau181 | (l) precentral volume | -0.007 (-0.014, 0.001) | 0.085 | 0.255 |
| p-tau181 | (l) precuneus volume | -0.009 (-0.014, -0.003) | 0.002 | 0.066 |
| p-tau181 | (l) rostralanteriorcingulate volume | 0.002 (-0.005, 0.008) | 0.604 | 0.767 |
| p-tau181 | (l) rostralmiddlefrontal volume | -0.007 (-0.012, -0.001) | 0.017 | 0.108 |
| p-tau181 | (l) superiorfrontal volume | -0.010 (-0.016, -0.004) | 0.001 | 0.066 |
| p-tau181 | (l) superiorparietal volume | -0.008 (-0.015, -0.002) | 0.011 | 0.108 |
| p-tau181 | (l) superiortemporal volume | -0.003 (-0.009, 0.003) | 0.341 | 0.512 |
| p-tau181 | (l) supramarginal volume | -0.002 (-0.007, 0.002) | 0.289 | 0.477 |
| p-tau181 | (l) transversetemporal volume | 0.000 (-0.006, 0.006) | 0.932 | 0.990 |
| p-tau181 | (r) bankssts volume | 0.000 (-0.006, 0.006) | 0.988 | 0.990 |
| p-tau181 | (r) caudalanteriorcingulate volume | 0.003 (-0.005, 0.011) | 0.467 | 0.604 |
| p-tau181 | (r) caudalmiddlefronta volume | -0.008 (-0.014, -0.002) | 0.011 | 0.108 |
| p-tau181 | (r) cuneus volume | 0.000 (-0.006, 0.007) | 0.906 | 0.990 |
| p-tau181 | (r) entorhinal volume | -0.005 (-0.017, 0.006) | 0.369 | 0.541 |
| p-tau181 | (r) frontalpole volume | 0.000 (-0.014, 0.014) | 0.990 | 0.990 |
| p-tau181 | (r) fusiform volume | -0.002 (-0.008, 0.003) | 0.420 | 0.570 |
| p-tau181 | (r) inferiorparietal volume | -0.001 (-0.006, 0.004) | 0.618 | 0.770 |
| p-tau181 | (r) inferiortemporal volume | -0.001 (-0.005, 0.004) | 0.837 | 0.986 |
| p-tau181 | (r) insula volume | -0.007 (-0.017, 0.003) | 0.173 | 0.433 |
| p-tau181 | (r) isthmuscingulate volume | -0.003 (-0.009, 0.004) | 0.397 | 0.570 |
| p-tau181 | (r) lateraloccipital volume | -0.004 (-0.010, 0.002) | 0.220 | 0.462 |
| p-tau181 | (r) lateralorbitofrontal volume | 0.004 (-0.006, 0.013) | 0.439 | 0.579 |
| p-tau181 | (r) lingual volume | -0.007 (-0.014, 0.000) | 0.056 | 0.205 |
| p-tau181 | (r) medialorbitofrontal volume | 0.000 (-0.008, 0.009) | 0.955 | 0.990 |
| p-tau181 | (r) middletemporal volume | -0.003 (-0.009, 0.003) | 0.304 | 0.489 |
| p-tau181 | (r) paracentral volume | -0.003 (-0.011, 0.005) | 0.409 | 0.570 |
| p-tau181 | (r) parahippocampal volume | 0.006 (-0.004, 0.017) | 0.225 | 0.462 |
| p-tau181 | (r) parsopercularis volume | -0.008 (-0.014, -0.003) | 0.003 | 0.066 |
| p-tau181 | (r) parsorbitalis volume | 0.004 (-0.004, 0.012) | 0.278 | 0.470 |
| p-tau181 | (r) parstriangularis volume | -0.006 (-0.011, 0.000) | 0.049 | 0.202 |
| p-tau181 | (r) pericalcarine volume | 0.000 (-0.007, 0.007) | 0.982 | 0.990 |
| p-tau181 | (r) postcentral volume | -0.007 (-0.014, 0.001) | 0.079 | 0.255 |
| p-tau181 | (r) posteriorcingulate volume | -0.004 (-0.012, 0.003) | 0.268 | 0.470 |
| p-tau181 | (r) precentral volume | -0.005 (-0.013, 0.004) | 0.268 | 0.470 |
| p-tau181 | (r) precuneus volume | -0.006 (-0.011, 0.000) | 0.043 | 0.202 |
| p-tau181 | (r) rostralanteriorcingulate volume | 0.000 (-0.006, 0.006) | 0.975 | 0.990 |
| p-tau181 | (r) rostralmiddlefrontal volume | -0.005 (-0.011, 0.000) | 0.052 | 0.202 |
| p-tau181 | (r) superiorfrontal volume | -0.007 (-0.013, -0.001) | 0.031 | 0.171 |
| p-tau181 | (r) superiorparietal volume | -0.006 (-0.014, 0.002) | 0.133 | 0.351 |
| p-tau181 | (r) superiortemporal volume | -0.001 (-0.007, 0.005) | 0.836 | 0.986 |
| p-tau181 | (r) supramarginal volume | -0.003 (-0.009, 0.003) | 0.328 | 0.512 |
| p-tau181 | (r) transversetemporal volume | 0.005 (-0.003, 0.013) | 0.231 | 0.462 |

Abbreviations: Aβ, amyloid-β; GFAP, glial fibrillary acidic protein; NfL, neurofilament light chain; p-tau181, tau phosphorylated at threonine 181; CI, confidence interval; l, left hemisphere; r, right hemisphere; FDR, false discovery rates.

Model was adjusted for sex, age, ethnicity, townsend deprivation index, assessment center, smoking status, alcohol use, family history of dementia, *APOE* ε4 allele, hypertension, diabetes, cardiovascular arterial disease, history of COVID-19 infection, and total intracranial volume.

The brain regions are as defined in the Desikan-Killiany atlas (cortex).

# Supplementary Table 10. Associations of baseline plasma AD-related biomarkers with longitudinal changes in cortical areas by using linear mixed-effects models.

| **Plasma biomarkers** | **Cortical areas** | **β (95%CI)** | ***P*-value** | ***FDR-***  ***corrected P*** |
| --- | --- | --- | --- | --- |
| Aβ42/40 | (l) bankssts area | -0.002 (-0.006, 0.003) | 0.529 | 0.794 |
| Aβ42/40 | (l) caudalanteriorcingulate area | -0.002 (-0.007, 0.003) | 0.506 | 0.777 |
| Aβ42/40 | (l) caudalmiddlefrontal area | 0.000 (-0.005, 0.004) | 0.872 | 0.914 |
| Aβ42/40 | (l) cuneus area | 0.002 (-0.001, 0.006) | 0.198 | 0.523 |
| Aβ42/40 | (l) entorhinal area | 0.002 (-0.008, 0.012) | 0.628 | 0.829 |
| Aβ42/40 | (l) frontalpole area | 0.004 (-0.008, 0.016) | 0.474 | 0.746 |
| Aβ42/40 | (l) fusiform area | 0.004 (-0.001, 0.008) | 0.123 | 0.419 |
| Aβ42/40 | (l) inferiorparietal area | 0.001 (-0.002, 0.004) | 0.417 | 0.714 |
| Aβ42/40 | (l) inferiortemporal area | 0.002 (-0.002, 0.006) | 0.348 | 0.693 |
| Aβ42/40 | (l) insula area | 0.006 (-0.007, 0.018) | 0.376 | 0.693 |
| Aβ42/40 | (l) isthmuscingulate area | -0.001 (-0.006, 0.004) | 0.740 | 0.864 |
| Aβ42/40 | (l) lateraloccipital area | 0.004 (0.001, 0.008) | 0.008 | 0.176 |
| Aβ42/40 | (l) lateralorbitofrontal area | 0.001 (-0.006, 0.007) | 0.812 | 0.893 |
| Aβ42/40 | (l) lingual area | 0.002 (-0.002, 0.005) | 0.330 | 0.681 |
| Aβ42/40 | (l) medialorbitofrontal area | 0.001 (-0.011, 0.013) | 0.871 | 0.914 |
| Aβ42/40 | (l) middletemporal area | 0.002 (-0.002, 0.005) | 0.422 | 0.714 |
| Aβ42/40 | (l) paracentral area | 0.003 (-0.002, 0.008) | 0.289 | 0.636 |
| Aβ42/40 | (l) parahippocampal area | 0.001 (-0.007, 0.010) | 0.759 | 0.864 |
| Aβ42/40 | (l) parsopercularis area | -0.003 (-0.007, 0.001) | 0.161 | 0.446 |
| Aβ42/40 | (l) parsorbitalis area | 0.006 (0.000, 0.013) | 0.039 | 0.257 |
| Aβ42/40 | (l) parstriangularis area | 0.001 (-0.003, 0.005) | 0.717 | 0.864 |
| Aβ42/40 | (l) pericalcarine area | 0.001 (-0.002, 0.004) | 0.596 | 0.807 |
| Aβ42/40 | (l) postcentral area | 0.004 (0.000, 0.007) | 0.031 | 0.256 |
| Aβ42/40 | (l) posteriorcingulate area | -0.001 (-0.006, 0.005) | 0.773 | 0.865 |
| Aβ42/40 | (l) precentral area | 0.003 (-0.002, 0.008) | 0.248 | 0.594 |
| Aβ42/40 | (l) precuneus area | 0.004 (0.001, 0.007) | 0.021 | 0.242 |
| Aβ42/40 | (l) rostralanteriorcingulate area | -0.004 (-0.011, 0.003) | 0.289 | 0.636 |
| Aβ42/40 | (l) rostralmiddlefrontal area | -0.004 (-0.008, -0.001) | 0.026 | 0.245 |
| Aβ42/40 | (l) superiorfrontal area | -0.002 (-0.005, 0.002) | 0.320 | 0.681 |
| Aβ42/40 | (l) superiorparietal area | 0.006 (0.002, 0.009) | 0.002 | 0.066 |
| Aβ42/40 | (l) superiortemporal area | 0.001 (-0.002, 0.004) | 0.582 | 0.807 |
| Aβ42/40 | (l) supramarginal area | 0.005 (0.002, 0.008) | 0.001 | 0.066 |
| Aβ42/40 | (l) transversetemporal area | -0.002 (-0.008, 0.005) | 0.583 | 0.807 |
| Aβ42/40 | (r) bankssts area | 0.005 (-0.001, 0.011) | 0.098 | 0.404 |
| Aβ42/40 | (r) caudalanteriorcingulate area | 0.004 (-0.001, 0.008) | 0.127 | 0.419 |
| Aβ42/40 | (r) caudalmiddlefronta area | 0.002 (-0.004, 0.008) | 0.475 | 0.746 |
| Aβ42/40 | (r) cuneus area | 0.001 (-0.004, 0.006) | 0.729 | 0.864 |
| Aβ42/40 | (r) entorhinal area | -0.007 (-0.016, 0.003) | 0.159 | 0.446 |
| Aβ42/40 | (r) frontalpole area | -0.012 (-0.024, -0.001) | 0.039 | 0.257 |
| Aβ42/40 | (r) fusiform area | 0.005 (0.001, 0.009) | 0.013 | 0.215 |
| Aβ42/40 | (r) inferiorparietal area | 0.004 (0.001, 0.007) | 0.022 | 0.242 |
| Aβ42/40 | (r) inferiortemporal area | 0.001 (-0.003, 0.005) | 0.757 | 0.864 |
| Aβ42/40 | (r) insula area | 0.010 (-0.004, 0.024) | 0.162 | 0.446 |
| Aβ42/40 | (r) isthmuscingulate area | 0.001 (-0.005, 0.008) | 0.667 | 0.862 |
| Aβ42/40 | (r) lateraloccipital area | 0.002 (-0.001, 0.005) | 0.252 | 0.594 |
| Aβ42/40 | (r) lateralorbitofrontal area | -0.003 (-0.016, 0.009) | 0.599 | 0.807 |
| Aβ42/40 | (r) lingual area | 0.002 (-0.002, 0.007) | 0.247 | 0.594 |
| Aβ42/40 | (r) medialorbitofrontal area | -0.007 (-0.016, 0.002) | 0.114 | 0.419 |
| Aβ42/40 | (r) middletemporal area | 0.003 (0.000, 0.006) | 0.067 | 0.339 |
| Aβ42/40 | (r) paracentral area | 0.000 (-0.005, 0.005) | 0.977 | 0.992 |
| Aβ42/40 | (r) parahippocampal area | 0.008 (-0.001, 0.017) | 0.067 | 0.339 |
| Aβ42/40 | (r) parsopercularis area | 0.000 (-0.005, 0.005) | 0.996 | 0.996 |
| Aβ42/40 | (r) parsorbitalis area | -0.001 (-0.007, 0.005) | 0.711 | 0.864 |
| Aβ42/40 | (r) parstriangularis area | 0.004 (0.000, 0.008) | 0.077 | 0.339 |
| Aβ42/40 | (r) pericalcarine area | 0.002 (-0.002, 0.005) | 0.408 | 0.714 |
| Aβ42/40 | (r) postcentral area | 0.003 (-0.001, 0.007) | 0.157 | 0.446 |
| Aβ42/40 | (r) posteriorcingulate area | 0.003 (-0.003, 0.008) | 0.362 | 0.693 |
| Aβ42/40 | (r) precentral area | 0.005 (0.000, 0.011) | 0.069 | 0.339 |
| Aβ42/40 | (r) precuneus area | 0.003 (0.000, 0.007) | 0.076 | 0.339 |
| Aβ42/40 | (r) rostralanteriorcingulate area | 0.000 (-0.006, 0.006) | 0.946 | 0.976 |
| Aβ42/40 | (r) rostralmiddlefrontal area | 0.001 (-0.003, 0.005) | 0.679 | 0.862 |
| Aβ42/40 | (r) superiorfrontal area | 0.001 (-0.003, 0.005) | 0.550 | 0.807 |
| Aβ42/40 | (r) superiorparietal area | 0.002 (-0.003, 0.006) | 0.462 | 0.746 |
| Aβ42/40 | (r) superiortemporal area | 0.002 (-0.002, 0.005) | 0.378 | 0.693 |
| Aβ42/40 | (r) supramarginal area | 0.000 (-0.004, 0.005) | 0.835 | 0.903 |
| Aβ42/40 | (r) transversetemporal area | 0.007 (-0.002, 0.016) | 0.121 | 0.419 |
| GFAP | (l) bankssts area | 0.000 (-0.005, 0.004) | 0.983 | 0.983 |
| GFAP | (l) caudalanteriorcingulate area | 0.002 (-0.002, 0.007) | 0.326 | 0.582 |
| GFAP | (l) caudalmiddlefrontal area | -0.001 (-0.005, 0.003) | 0.651 | 0.767 |
| GFAP | (l) cuneus area | -0.001 (-0.005, 0.002) | 0.508 | 0.699 |
| GFAP | (l) entorhinal area | 0.005 (-0.004, 0.014) | 0.297 | 0.577 |
| GFAP | (l) frontalpole area | 0.007 (-0.004, 0.019) | 0.198 | 0.436 |
| GFAP | (l) fusiform area | -0.007 (-0.011, -0.003) | 0.002 | 0.033 |
| GFAP | (l) inferiorparietal area | -0.002 (-0.005, 0.000) | 0.091 | 0.315 |
| GFAP | (l) inferiortemporal area | -0.003 (-0.007, 0.001) | 0.140 | 0.385 |
| GFAP | (l) insula area | -0.003 (-0.015, 0.009) | 0.614 | 0.765 |
| GFAP | (l) isthmuscingulate area | 0.003 (-0.002, 0.008) | 0.184 | 0.421 |
| GFAP | (l) lateraloccipital area | -0.002 (-0.005, 0.001) | 0.166 | 0.406 |
| GFAP | (l) lateralorbitofrontal area | -0.007 (-0.013, -0.001) | 0.028 | 0.151 |
| GFAP | (l) lingual area | 0.002 (-0.002, 0.005) | 0.369 | 0.605 |
| GFAP | (l) medialorbitofrontal area | -0.005 (-0.016, 0.007) | 0.401 | 0.605 |
| GFAP | (l) middletemporal area | -0.002 (-0.006, 0.001) | 0.238 | 0.491 |
| GFAP | (l) paracentral area | 0.002 (-0.003, 0.007) | 0.489 | 0.687 |
| GFAP | (l) parahippocampal area | -0.002 (-0.010, 0.006) | 0.629 | 0.767 |
| GFAP | (l) parsopercularis area | -0.001 (-0.004, 0.003) | 0.665 | 0.770 |
| GFAP | (l) parsorbitalis area | -0.009 (-0.015, -0.003) | 0.003 | 0.040 |
| GFAP | (l) parstriangularis area | -0.001 (-0.005, 0.003) | 0.520 | 0.700 |
| GFAP | (l) pericalcarine area | 0.000 (-0.003, 0.003) | 0.888 | 0.945 |
| GFAP | (l) postcentral area | -0.001 (-0.005, 0.002) | 0.346 | 0.601 |
| GFAP | (l) posteriorcingulate area | 0.000 (-0.006, 0.005) | 0.881 | 0.945 |
| GFAP | (l) precentral area | 0.002 (-0.003, 0.006) | 0.403 | 0.605 |
| GFAP | (l) precuneus area | -0.002 (-0.005, 0.001) | 0.214 | 0.456 |
| GFAP | (l) rostralanteriorcingulate area | 0.000 (-0.006, 0.007) | 0.930 | 0.956 |
| GFAP | (l) rostralmiddlefrontal area | -0.004 (-0.007, -0.001) | 0.014 | 0.110 |
| GFAP | (l) superiorfrontal area | -0.003 (-0.006, 0.000) | 0.050 | 0.220 |
| GFAP | (l) superiorparietal area | -0.002 (-0.004, 0.001) | 0.306 | 0.577 |
| GFAP | (l) superiortemporal area | -0.006 (-0.009, -0.003) | 0.000 | 0.000 |
| GFAP | (l) supramarginal area | -0.002 (-0.005, 0.000) | 0.081 | 0.297 |
| GFAP | (l) transversetemporal area | -0.006 (-0.012, 0.000) | 0.065 | 0.264 |
| GFAP | (r) bankssts area | -0.002 (-0.007, 0.004) | 0.561 | 0.726 |
| GFAP | (r) caudalanteriorcingulate area | -0.005 (-0.009, -0.001) | 0.027 | 0.151 |
| GFAP | (r) caudalmiddlefronta area | -0.004 (-0.008, 0.001) | 0.151 | 0.399 |
| GFAP | (r) cuneus area | -0.003 (-0.008, 0.001) | 0.163 | 0.406 |
| GFAP | (r) entorhinal area | -0.014 (-0.023, -0.005) | 0.002 | 0.033 |
| GFAP | (r) frontalpole area | 0.005 (-0.006, 0.016) | 0.399 | 0.605 |
| GFAP | (r) fusiform area | -0.001 (-0.005, 0.003) | 0.582 | 0.739 |
| GFAP | (r) inferiorparietal area | 0.000 (-0.003, 0.002) | 0.779 | 0.871 |
| GFAP | (r) inferiortemporal area | -0.003 (-0.007, 0.000) | 0.068 | 0.264 |
| GFAP | (r) insula area | 0.006 (-0.007, 0.019) | 0.363 | 0.605 |
| GFAP | (r) isthmuscingulate area | -0.002 (-0.008, 0.004) | 0.449 | 0.659 |
| GFAP | (r) lateraloccipital area | 0.002 (-0.002, 0.005) | 0.321 | 0.582 |
| GFAP | (r) lateralorbitofrontal area | -0.010 (-0.022, 0.002) | 0.096 | 0.315 |
| GFAP | (r) lingual area | 0.000 (-0.004, 0.004) | 0.910 | 0.953 |
| GFAP | (r) medialorbitofrontal area | -0.001 (-0.010, 0.007) | 0.737 | 0.839 |
| GFAP | (r) middletemporal area | -0.004 (-0.007, -0.001) | 0.008 | 0.075 |
| GFAP | (r) paracentral area | -0.001 (-0.005, 0.004) | 0.803 | 0.883 |
| GFAP | (r) parahippocampal area | -0.004 (-0.012, 0.005) | 0.399 | 0.605 |
| GFAP | (r) parsopercularis area | -0.005 (-0.010, -0.001) | 0.030 | 0.151 |
| GFAP | (r) parsorbitalis area | -0.005 (-0.010, 0.001) | 0.105 | 0.315 |
| GFAP | (r) parstriangularis area | -0.003 (-0.007, 0.001) | 0.185 | 0.421 |
| GFAP | (r) pericalcarine area | -0.001 (-0.005, 0.002) | 0.473 | 0.679 |
| GFAP | (r) postcentral area | 0.000 (-0.004, 0.004) | 0.942 | 0.956 |
| GFAP | (r) posteriorcingulate area | -0.009 (-0.014, -0.004) | 0.001 | 0.033 |
| GFAP | (r) precentral area | -0.002 (-0.006, 0.003) | 0.539 | 0.711 |
| GFAP | (r) precuneus area | -0.002 (-0.006, 0.001) | 0.133 | 0.382 |
| GFAP | (r) rostralanteriorcingulate area | -0.008 (-0.013, -0.002) | 0.007 | 0.075 |
| GFAP | (r) rostralmiddlefrontal area | -0.004 (-0.008, 0.000) | 0.031 | 0.151 |
| GFAP | (r) superiorfrontal area | -0.004 (-0.008, 0.000) | 0.032 | 0.151 |
| GFAP | (r) superiorparietal area | 0.003 (-0.001, 0.008) | 0.103 | 0.315 |
| GFAP | (r) superiortemporal area | -0.001 (-0.004, 0.003) | 0.648 | 0.767 |
| GFAP | (r) supramarginal area | 0.002 (-0.002, 0.006) | 0.262 | 0.524 |
| GFAP | (r) transversetemporal area | -0.010 (-0.018, -0.002) | 0.015 | 0.110 |
| NfL | (l) bankssts area | -0.001 (-0.005, 0.003) | 0.656 | 0.821 |
| NfL | (l) caudalanteriorcingulate area | -0.001 (-0.006, 0.003) | 0.607 | 0.821 |
| NfL | (l) caudalmiddlefrontal area | -0.004 (-0.007, 0.000) | 0.073 | 0.241 |
| NfL | (l) cuneus area | -0.002 (-0.005, 0.002) | 0.356 | 0.613 |
| NfL | (l) entorhinal area | -0.002 (-0.011, 0.007) | 0.631 | 0.821 |
| NfL | (l) frontalpole area | 0.006 (-0.005, 0.017) | 0.280 | 0.578 |
| NfL | (l) fusiform area | -0.006 (-0.010, -0.002) | 0.006 | 0.044 |
| NfL | (l) inferiorparietal area | -0.001 (-0.003, 0.002) | 0.634 | 0.821 |
| NfL | (l) inferiortemporal area | -0.005 (-0.009, -0.001) | 0.012 | 0.066 |
| NfL | (l) insula area | -0.001 (-0.012, 0.011) | 0.911 | 0.911 |
| NfL | (l) isthmuscingulate area | 0.002 (-0.002, 0.007) | 0.311 | 0.592 |
| NfL | (l) lateraloccipital area | -0.003 (-0.006, 0.000) | 0.044 | 0.171 |
| NfL | (l) lateralorbitofrontal area | -0.007 (-0.013, -0.001) | 0.017 | 0.079 |
| NfL | (l) lingual area | -0.001 (-0.004, 0.003) | 0.660 | 0.821 |
| NfL | (l) medialorbitofrontal area | -0.009 (-0.020, 0.002) | 0.118 | 0.325 |
| NfL | (l) middletemporal area | -0.002 (-0.006, 0.001) | 0.194 | 0.453 |
| NfL | (l) paracentral area | -0.003 (-0.008, 0.002) | 0.199 | 0.453 |
| NfL | (l) parahippocampal area | -0.004 (-0.012, 0.004) | 0.323 | 0.592 |
| NfL | (l) parsopercularis area | -0.007 (-0.011, -0.003) | 0.001 | 0.017 |
| NfL | (l) parsorbitalis area | -0.009 (-0.014, -0.003) | 0.002 | 0.026 |
| NfL | (l) parstriangularis area | -0.004 (-0.007, 0.000) | 0.062 | 0.215 |
| NfL | (l) pericalcarine area | 0.000 (-0.003, 0.003) | 0.877 | 0.911 |
| NfL | (l) postcentral area | 0.001 (-0.002, 0.005) | 0.362 | 0.613 |
| NfL | (l) posteriorcingulate area | -0.001 (-0.006, 0.004) | 0.728 | 0.843 |
| NfL | (l) precentral area | 0.003 (-0.002, 0.007) | 0.246 | 0.541 |
| NfL | (l) precuneus area | -0.001 (-0.004, 0.003) | 0.722 | 0.843 |
| NfL | (l) rostralanteriorcingulate area | -0.002 (-0.009, 0.004) | 0.490 | 0.752 |
| NfL | (l) rostralmiddlefrontal area | -0.008 (-0.012, -0.005) | 0.000 | 0.000 |
| NfL | (l) superiorfrontal area | -0.006 (-0.009, -0.004) | 0.000 | 0.000 |
| NfL | (l) superiorparietal area | 0.001 (-0.002, 0.004) | 0.600 | 0.821 |
| NfL | (l) superiortemporal area | -0.005 (-0.008, -0.001) | 0.004 | 0.033 |
| NfL | (l) supramarginal area | 0.000 (-0.003, 0.003) | 0.903 | 0.911 |
| NfL | (l) transversetemporal area | -0.002 (-0.008, 0.004) | 0.442 | 0.712 |
| NfL | (r) bankssts area | -0.002 (-0.007, 0.004) | 0.566 | 0.821 |
| NfL | (r) caudalanteriorcingulate area | -0.003 (-0.008, 0.001) | 0.131 | 0.346 |
| NfL | (r) caudalmiddlefronta area | -0.001 (-0.006, 0.004) | 0.833 | 0.898 |
| NfL | (r) cuneus area | -0.004 (-0.009, 0.000) | 0.059 | 0.215 |
| NfL | (r) entorhinal area | -0.011 (-0.019, -0.002) | 0.014 | 0.071 |
| NfL | (r) frontalpole area | 0.002 (-0.008, 0.013) | 0.686 | 0.823 |
| NfL | (r) fusiform area | 0.000 (-0.004, 0.003) | 0.911 | 0.911 |
| NfL | (r) inferiorparietal area | -0.002 (-0.005, 0.001) | 0.262 | 0.558 |
| NfL | (r) inferiortemporal area | -0.005 (-0.009, -0.002) | 0.003 | 0.033 |
| NfL | (r) insula area | -0.007 (-0.019, 0.006) | 0.297 | 0.592 |
| NfL | (r) isthmuscingulate area | -0.005 (-0.010, 0.001) | 0.093 | 0.279 |
| NfL | (r) lateraloccipital area | -0.001 (-0.004, 0.002) | 0.488 | 0.752 |
| NfL | (r) lateralorbitofrontal area | -0.003 (-0.015, 0.008) | 0.594 | 0.821 |
| NfL | (r) lingual area | -0.002 (-0.006, 0.001) | 0.196 | 0.453 |
| NfL | (r) medialorbitofrontal area | -0.001 (-0.009, 0.007) | 0.812 | 0.898 |
| NfL | (r) middletemporal area | -0.004 (-0.007, -0.001) | 0.004 | 0.033 |
| NfL | (r) paracentral area | -0.001 (-0.005, 0.004) | 0.830 | 0.898 |
| NfL | (r) parahippocampal area | 0.002 (-0.006, 0.010) | 0.672 | 0.821 |
| NfL | (r) parsopercularis area | -0.006 (-0.010, -0.001) | 0.018 | 0.079 |
| NfL | (r) parsorbitalis area | -0.002 (-0.007, 0.004) | 0.544 | 0.816 |
| NfL | (r) parstriangularis area | -0.003 (-0.007, 0.001) | 0.092 | 0.279 |
| NfL | (r) pericalcarine area | -0.001 (-0.005, 0.002) | 0.394 | 0.650 |
| NfL | (r) postcentral area | -0.001 (-0.005, 0.003) | 0.655 | 0.821 |
| NfL | (r) posteriorcingulate area | -0.010 (-0.015, -0.005) | 0.000 | 0.000 |
| NfL | (r) precentral area | 0.002 (-0.003, 0.008) | 0.343 | 0.612 |
| NfL | (r) precuneus area | -0.004 (-0.008, -0.001) | 0.012 | 0.066 |
| NfL | (r) rostralanteriorcingulate area | -0.007 (-0.013, -0.002) | 0.007 | 0.046 |
| NfL | (r) rostralmiddlefrontal area | -0.004 (-0.008, -0.001) | 0.022 | 0.091 |
| NfL | (r) superiorfrontal area | -0.003 (-0.007, 0.001) | 0.152 | 0.386 |
| NfL | (r) superiorparietal area | -0.001 (-0.005, 0.004) | 0.784 | 0.892 |
| NfL | (r) superiortemporal area | -0.003 (-0.006, 0.001) | 0.114 | 0.325 |
| NfL | (r) supramarginal area | 0.000 (-0.004, 0.004) | 0.844 | 0.898 |
| NfL | (r) transversetemporal area | -0.004 (-0.012, 0.004) | 0.320 | 0.592 |
| p-tau181 | (l) bankssts area | 0.000 (-0.004, 0.004) | 0.999 | 0.999 |
| p-tau181 | (l) caudalanteriorcingulate area | 0.001 (-0.004, 0.005) | 0.760 | 0.896 |
| p-tau181 | (l) caudalmiddlefrontal area | -0.002 (-0.005, 0.002) | 0.431 | 0.884 |
| p-tau181 | (l) cuneus area | 0.002 (-0.001, 0.006) | 0.172 | 0.657 |
| p-tau181 | (l) entorhinal area | -0.002 (-0.011, 0.008) | 0.723 | 0.884 |
| p-tau181 | (l) frontalpole area | -0.001 (-0.012, 0.010) | 0.860 | 0.996 |
| p-tau181 | (l) fusiform area | 0.001 (-0.003, 0.005) | 0.684 | 0.884 |
| p-tau181 | (l) inferiorparietal area | -0.003 (-0.005, 0.000) | 0.052 | 0.657 |
| p-tau181 | (l) inferiortemporal area | -0.003 (-0.007, 0.001) | 0.105 | 0.657 |
| p-tau181 | (l) insula area | -0.002 (-0.014, 0.009) | 0.678 | 0.884 |
| p-tau181 | (l) isthmuscingulate area | 0.001 (-0.004, 0.005) | 0.742 | 0.890 |
| p-tau181 | (l) lateraloccipital area | -0.003 (-0.006, 0.000) | 0.071 | 0.657 |
| p-tau181 | (l) lateralorbitofrontal area | -0.003 (-0.009, 0.003) | 0.394 | 0.884 |
| p-tau181 | (l) lingual area | 0.003 (0.000, 0.006) | 0.057 | 0.657 |
| p-tau181 | (l) medialorbitofrontal area | 0.003 (-0.008, 0.014) | 0.631 | 0.884 |
| p-tau181 | (l) middletemporal area | -0.003 (-0.006, 0.000) | 0.071 | 0.657 |
| p-tau181 | (l) paracentral area | -0.003 (-0.008, 0.002) | 0.185 | 0.657 |
| p-tau181 | (l) parahippocampal area | -0.001 (-0.009, 0.006) | 0.722 | 0.884 |
| p-tau181 | (l) parsopercularis area | -0.001 (-0.005, 0.003) | 0.526 | 0.884 |
| p-tau181 | (l) parsorbitalis area | -0.005 (-0.011, 0.000) | 0.060 | 0.657 |
| p-tau181 | (l) parstriangularis area | 0.000 (-0.004, 0.004) | 0.955 | 0.999 |
| p-tau181 | (l) pericalcarine area | 0.002 (-0.001, 0.005) | 0.179 | 0.657 |
| p-tau181 | (l) postcentral area | 0.002 (-0.001, 0.005) | 0.221 | 0.695 |
| p-tau181 | (l) posteriorcingulate area | 0.002 (-0.003, 0.007) | 0.394 | 0.884 |
| p-tau181 | (l) precentral area | 0.002 (-0.003, 0.006) | 0.457 | 0.884 |
| p-tau181 | (l) precuneus area | -0.002 (-0.006, 0.001) | 0.121 | 0.657 |
| p-tau181 | (l) rostralanteriorcingulate area | -0.002 (-0.008, 0.005) | 0.565 | 0.884 |
| p-tau181 | (l) rostralmiddlefrontal area | -0.001 (-0.005, 0.002) | 0.483 | 0.884 |
| p-tau181 | (l) superiorfrontal area | 0.000 (-0.003, 0.003) | 0.899 | 0.999 |
| p-tau181 | (l) superiorparietal area | -0.001 (-0.004, 0.002) | 0.620 | 0.884 |
| p-tau181 | (l) superiortemporal area | -0.003 (-0.006, 0.000) | 0.059 | 0.657 |
| p-tau181 | (l) supramarginal area | 0.000 (-0.003, 0.003) | 0.950 | 0.999 |
| p-tau181 | (l) transversetemporal area | 0.001 (-0.005, 0.007) | 0.684 | 0.884 |
| p-tau181 | (r) bankssts area | -0.001 (-0.007, 0.004) | 0.688 | 0.884 |
| p-tau181 | (r) caudalanteriorcingulate area | -0.001 (-0.006, 0.003) | 0.566 | 0.884 |
| p-tau181 | (r) caudalmiddlefronta area | -0.001 (-0.006, 0.004) | 0.700 | 0.884 |
| p-tau181 | (r) cuneus area | 0.003 (-0.002, 0.007) | 0.240 | 0.720 |
| p-tau181 | (r) entorhinal area | -0.006 (-0.014, 0.003) | 0.176 | 0.657 |
| p-tau181 | (r) frontalpole area | 0.008 (-0.003, 0.019) | 0.150 | 0.657 |
| p-tau181 | (r) fusiform area | 0.000 (-0.004, 0.004) | 0.979 | 0.999 |
| p-tau181 | (r) inferiorparietal area | 0.001 (-0.002, 0.004) | 0.506 | 0.884 |
| p-tau181 | (r) inferiortemporal area | 0.000 (-0.003, 0.004) | 0.910 | 0.999 |
| p-tau181 | (r) insula area | -0.003 (-0.016, 0.010) | 0.659 | 0.884 |
| p-tau181 | (r) isthmuscingulate area | -0.004 (-0.009, 0.002) | 0.203 | 0.670 |
| p-tau181 | (r) lateraloccipital area | 0.001 (-0.002, 0.004) | 0.674 | 0.884 |
| p-tau181 | (r) lateralorbitofrontal area | 0.008 (-0.004, 0.020) | 0.175 | 0.657 |
| p-tau181 | (r) lingual area | 0.000 (-0.003, 0.004) | 0.878 | 0.999 |
| p-tau181 | (r) medialorbitofrontal area | 0.005 (-0.003, 0.013) | 0.262 | 0.752 |
| p-tau181 | (r) middletemporal area | -0.001 (-0.004, 0.002) | 0.435 | 0.884 |
| p-tau181 | (r) paracentral area | 0.000 (-0.005, 0.005) | 0.984 | 0.999 |
| p-tau181 | (r) parahippocampal area | 0.006 (-0.003, 0.014) | 0.189 | 0.657 |
| p-tau181 | (r) parsopercularis area | -0.005 (-0.010, -0.001) | 0.028 | 0.657 |
| p-tau181 | (r) parsorbitalis area | 0.001 (-0.004, 0.006) | 0.668 | 0.884 |
| p-tau181 | (r) parstriangularis area | -0.003 (-0.007, 0.001) | 0.101 | 0.657 |
| p-tau181 | (r) pericalcarine area | 0.001 (-0.003, 0.004) | 0.720 | 0.884 |
| p-tau181 | (r) postcentral area | 0.001 (-0.003, 0.005) | 0.633 | 0.884 |
| p-tau181 | (r) posteriorcingulate area | -0.004 (-0.009, 0.001) | 0.116 | 0.657 |
| p-tau181 | (r) precentral area | 0.002 (-0.003, 0.008) | 0.350 | 0.884 |
| p-tau181 | (r) precuneus area | -0.003 (-0.006, 0.001) | 0.123 | 0.657 |
| p-tau181 | (r) rostralanteriorcingulate area | 0.001 (-0.004, 0.006) | 0.722 | 0.884 |
| p-tau181 | (r) rostralmiddlefrontal area | -0.001 (-0.005, 0.003) | 0.576 | 0.884 |
| p-tau181 | (r) superiorfrontal area | -0.002 (-0.006, 0.002) | 0.363 | 0.884 |
| p-tau181 | (r) superiorparietal area | 0.002 (-0.002, 0.007) | 0.288 | 0.792 |
| p-tau181 | (r) superiortemporal area | 0.000 (-0.003, 0.003) | 0.982 | 0.999 |
| p-tau181 | (r) supramarginal area | 0.001 (-0.003, 0.005) | 0.614 | 0.884 |
| p-tau181 | (r) transversetemporal area | 0.002 (-0.006, 0.010) | 0.624 | 0.884 |

Abbreviations: Aβ, amyloid-β; GFAP, glial fibrillary acidic protein; NfL, neurofilament light chain; p-tau181, tau phosphorylated at threonine 181; CI, confidence interval; l, left hemisphere; r, right hemisphere; FDR, false discovery rates.

Model was adjusted for sex, age, ethnicity, townsend deprivation index, assessment center, smoking status, alcohol use, family history of dementia, *APOE* ε4 allele, hypertension, diabetes, cardiovascular arterial disease, history of COVID-19 infection, and total intracranial volume.

The brain regions are as defined in the Desikan-Killiany atlas (cortex).

# Supplementary Table 11. Associations of baseline plasma AD-related biomarkers with longitudinal changes in subcortical brain volumes by using linear mixed-effects models.

| **Plasma biomarkers** | **Subcortical brain volumes** | **β (95% CI)** | ***P*-value** | ***FDR-***  ***corrected P*** |
| --- | --- | --- | --- | --- |
| Aβ42/40 | Right_Thalamus_Proper | 0.006 (-0.001, 0.012) | 0.090 | 0.360 |
| Aβ42/40 | Right_Putamen | 0.001 (-0.006, 0.007) | 0.847 | 0.917 |
| Aβ42/40 | Right_Pallidum | 0.000 (-0.008, 0.008) | 0.962 | 0.962 |
| Aβ42/40 | Right_Lateral_Ventricle | -0.002 (-0.004, 0.001) | 0.291 | 0.723 |
| Aβ42/40 | Right_Hippocampus | 0.009 (0.002, 0.016) | 0.010 | 0.160 |
| Aβ42/40 | Right_Caudate | 0.001 (-0.006, 0.008) | 0.786 | 0.917 |
| Aβ42/40 | Right_Amygdala | 0.004 (-0.004, 0.013) | 0.336 | 0.723 |
| Aβ42/40 | Right_Accumbens_area | 0.003 (-0.006, 0.013) | 0.487 | 0.723 |
| Aβ42/40 | Left_Thalamus_Proper | -0.001 (-0.009, 0.007) | 0.770 | 0.917 |
| Aβ42/40 | Left_Putamen | 0.002 (-0.004, 0.009) | 0.461 | 0.723 |
| Aβ42/40 | Left_Pallidum | 0.003 (-0.006, 0.012) | 0.497 | 0.723 |
| Aβ42/40 | Left_Lateral_Ventricle | -0.002 (-0.004, 0.001) | 0.267 | 0.723 |
| Aβ42/40 | Left_Hippocampus | 0.006 (-0.001, 0.013) | 0.081 | 0.360 |
| Aβ42/40 | Left_Caudate | 0.002 (-0.003, 0.007) | 0.450 | 0.723 |
| Aβ42/40 | Left_Amygdala | 0.008 (-0.001, 0.018) | 0.084 | 0.360 |
| Aβ42/40 | Left_Accumbens_area | -0.001 (-0.012, 0.010) | 0.860 | 0.917 |
| GFAP | Right_Thalamus_Proper | -0.008 (-0.014, -0.002) | 0.009 | 0.029 |
| GFAP | Right_Putamen | -0.006 (-0.011, 0.000) | 0.044 | 0.094 |
| GFAP | Right_Pallidum | -0.002 (-0.010, 0.005) | 0.529 | 0.605 |
| GFAP | Right_Lateral_Ventricle | 0.007 (0.004, 0.010) | <0.001 | 0.000 |
| GFAP | Right_Hippocampus | -0.014 (-0.020, -0.008) | <0.001 | 0.000 |
| GFAP | Right_Caudate | -0.001 (-0.008, 0.005) | 0.667 | 0.711 |
| GFAP | Right_Amygdala | -0.006 (-0.014, 0.002) | 0.136 | 0.218 |
| GFAP | Right_Accumbens_area | -0.005 (-0.014, 0.004) | 0.284 | 0.350 |
| GFAP | Left_Thalamus_Proper | -0.004 (-0.012, 0.003) | 0.277 | 0.350 |
| GFAP | Left_Putamen | -0.004 (-0.010, 0.002) | 0.173 | 0.252 |
| GFAP | Left_Pallidum | -0.007 (-0.016, 0.001) | 0.097 | 0.172 |
| GFAP | Left_Lateral_Ventricle | 0.006 (0.004, 0.009) | <0.001 | <0.001 |
| GFAP | Left_Hippocampus | -0.011 (-0.018, -0.004) | 0.001 | <0.001 |
| GFAP | Left_Caudate | 0.000 (-0.005, 0.005) | 0.973 | 0.973 |
| GFAP | Left_Amygdala | -0.011 (-0.020, -0.002) | 0.014 | 0.037 |
| GFAP | Left_Accumbens_area | -0.011 (-0.021, 0.000) | 0.047 | 0.094 |
| NfL | Right_Thalamus_Proper | -0.006 (-0.012, 0.000) | 0.048 | 0.096 |
| NfL | Right_Putamen | -0.002 (-0.008, 0.003) | 0.396 | 0.453 |
| NfL | Right_Pallidum | -0.006 (-0.013, 0.001) | 0.098 | 0.157 |
| NfL | Right_Lateral_Ventricle | 0.008 (0.005, 0.010) | <0.001 | 0.000 |
| NfL | Right_Hippocampus | -0.014 (-0.020, -0.008) | <0.001 | 0.000 |
| NfL | Right_Caudate | 0.001 (-0.005, 0.007) | 0.843 | 0.843 |
| NfL | Right_Amygdala | -0.009 (-0.017, -0.001) | 0.020 | 0.053 |
| NfL | Right_Accumbens_area | -0.010 (-0.019, -0.001) | 0.035 | 0.080 |
| NfL | Left_Thalamus_Proper | -0.006 (-0.013, 0.001) | 0.115 | 0.167 |
| NfL | Left_Putamen | -0.003 (-0.009, 0.003) | 0.343 | 0.422 |
| NfL | Left_Pallidum | -0.011 (-0.019, -0.003) | 0.008 | 0.026 |
| NfL | Left_Lateral_Ventricle | 0.007 (0.004, 0.010) | <0.001 | <0.001 |
| NfL | Left_Hippocampus | -0.011 (-0.017, -0.004) | 0.001 | 0.004 |
| NfL | Left_Caudate | -0.001 (-0.006, 0.004) | 0.693 | 0.739 |
| NfL | Left_Amygdala | -0.008 (-0.017, 0.000) | 0.064 | 0.114 |
| NfL | Left_Accumbens_area | -0.008 (-0.018, 0.002) | 0.141 | 0.188 |
| p-tau181 | Right_Thalamus_Proper | -0.004 (-0.010, 0.002) | 0.240 | 0.480 |
| p-tau181 | Right_Putamen | 0.000 (-0.006, 0.006) | 0.984 | 0.984 |
| p-tau181 | Right_Pallidum | -0.004 (-0.011, 0.003) | 0.302 | 0.537 |
| p-tau181 | Right_Lateral_Ventricle | 0.003 (0.000, 0.005) | 0.039 | 0.156 |
| p-tau181 | Right_Hippocampus | -0.015 (-0.021, -0.009) | <0.001 | <0.001 |
| p-tau181 | Right_Caudate | -0.004 (-0.010, 0.002) | 0.215 | 0.480 |
| p-tau181 | Right_Amygdala | -0.001 (-0.009, 0.006) | 0.703 | 0.863 |
| p-tau181 | Right_Accumbens_area | -0.002 (-0.011, 0.007) | 0.599 | 0.863 |
| p-tau181 | Left_Thalamus_Proper | -0.001 (-0.008, 0.006) | 0.794 | 0.863 |
| p-tau181 | Left_Putamen | -0.007 (-0.012, -0.001) | 0.024 | 0.128 |
| p-tau181 | Left_Pallidum | -0.002 (-0.010, 0.007) | 0.720 | 0.863 |
| p-tau181 | Left_Lateral_Ventricle | 0.002 (-0.001, 0.005) | 0.120 | 0.320 |
| p-tau181 | Left_Hippocampus | -0.012 (-0.019, -0.006) | <0.001 | <0.001 |
| p-tau181 | Left_Caudate | -0.001 (-0.006, 0.004) | 0.809 | 0.863 |
| p-tau181 | Left_Amygdala | -0.008 (-0.017, 0.001) | 0.076 | 0.243 |
| p-tau181 | Left_Accumbens_area | -0.003 (-0.014, 0.007) | 0.509 | 0.814 |

Abbreviations: Aβ, amyloid-β; GFAP, glial fibrillary acidic protein; NfL, neurofilament light chain; p-tau181, tau phosphorylated at threonine 181; CI, confidence interval; FDR, false discovery rates.

Model was adjusted for sex, age, ethnicity, townsend deprivation index, assessment center, smoking status, alcohol use, family history of dementia, *APOE* ε4 allele, hypertension, diabetes, cardiovascular arterial disease, history of COVID-19 infection, and total intracranial volume.

The brain regions are as defined in the ASEG atlas (subcortex).

# Supplementary Table 12. Associations of longitudinal plasma AD-related biomarkers with longitudinal changes in cortical gray volumes by using linear mixed-effects models.

| **Plasma biomarkers** | **Cortical gray volumes** | **β (95%CI)** | ***P-value*** | ***FDR-***  ***corrected P*** |
| --- | --- | --- | --- | --- |
| Aβ42/40 | (l) bankssts volume | 0.001 (-0.007, 0.009) | 0.822 | 0.875 |
| Aβ42/40 | (l) caudalanteriorcingulate volume | -0.008 (-0.020, 0.004) | 0.202 | 0.360 |
| Aβ42/40 | (l) caudalmiddlefrontal volume | 0.004 (-0.003, 0.012) | 0.247 | 0.388 |
| Aβ42/40 | (l) cuneus volume | -0.007 (-0.016, 0.002) | 0.133 | 0.349 |
| Aβ42/40 | (l) entorhinal volume | 0.002 (-0.014, 0.017) | 0.835 | 0.875 |
| Aβ42/40 | (l) frontalpole volume | -0.012 (-0.031, 0.006) | 0.178 | 0.349 |
| Aβ42/40 | (l) fusiform volume | -0.006 (-0.015, 0.002) | 0.148 | 0.349 |
| Aβ42/40 | (l) inferiorparietal volume | -0.002 (-0.009, 0.004) | 0.477 | 0.572 |
| Aβ42/40 | (l) inferiortemporal volume | -0.006 (-0.013, 0.001) | 0.120 | 0.349 |
| Aβ42/40 | (l) insula volume | 0.000 (-0.012, 0.013) | 0.944 | 0.944 |
| Aβ42/40 | (l) isthmuscingulate volume | -0.006 (-0.014, 0.001) | 0.111 | 0.349 |
| Aβ42/40 | (l) lateraloccipital volume | -0.007 (-0.016, 0.003) | 0.152 | 0.349 |
| Aβ42/40 | (l) lateralorbitofrontal volume | -0.002 (-0.011, 0.007) | 0.672 | 0.751 |
| Aβ42/40 | (l) lingual volume | -0.009 (-0.019, 0.001) | 0.065 | 0.252 |
| Aβ42/40 | (l) medialorbitofrontal volume | -0.011 (-0.026, 0.004) | 0.146 | 0.349 |
| Aβ42/40 | (l) middletemporal volume | -0.009 (-0.017, 0.000) | 0.046 | 0.248 |
| Aβ42/40 | (l) paracentral volume | -0.011 (-0.023, 0.000) | 0.052 | 0.248 |
| Aβ42/40 | (l) parahippocampal volume | -0.009 (-0.022, 0.004) | 0.168 | 0.349 |
| Aβ42/40 | (l) parsopercularis volume | -0.004 (-0.011, 0.004) | 0.325 | 0.438 |
| Aβ42/40 | (l) parsorbitalis volume | -0.009 (-0.020, 0.002) | 0.096 | 0.333 |
| Aβ42/40 | (l) parstriangularis volume | -0.007 (-0.014, 0.000) | 0.060 | 0.248 |
| Aβ42/40 | (l) pericalcarine volume | -0.001 (-0.009, 0.008) | 0.885 | 0.913 |
| Aβ42/40 | (l) postcentral volume | -0.009 (-0.019, 0.000) | 0.050 | 0.248 |
| Aβ42/40 | (l) posteriorcingulate volume | -0.005 (-0.015, 0.004) | 0.291 | 0.409 |
| Aβ42/40 | (l) precentral volume | -0.007 (-0.018, 0.004) | 0.219 | 0.380 |
| Aβ42/40 | (l) precuneus volume | -0.005 (-0.012, 0.003) | 0.238 | 0.383 |
| Aβ42/40 | (l) rostralanteriorcingulate volume | -0.008 (-0.016, 0.001) | 0.076 | 0.279 |
| Aβ42/40 | (l) rostralmiddlefrontal volume | -0.008 (-0.016, -0.001) | 0.033 | 0.248 |
| Aβ42/40 | (l) superiorfrontal volume | -0.006 (-0.014, 0.003) | 0.191 | 0.356 |
| Aβ42/40 | (l) superiorparietal volume | -0.004 (-0.013, 0.005) | 0.438 | 0.545 |
| Aβ42/40 | (l) superiortemporal volume | -0.012 (-0.020, -0.004) | 0.003 | 0.198 |
| Aβ42/40 | (l) supramarginal volume | -0.003 (-0.010, 0.003) | 0.307 | 0.422 |
| Aβ42/40 | (l) transversetemporal volume | -0.006 (-0.014, 0.003) | 0.180 | 0.349 |
| Aβ42/40 | (r) bankssts volume | -0.004 (-0.012, 0.005) | 0.392 | 0.507 |
| Aβ42/40 | (r) caudalanteriorcingulate volume | -0.002 (-0.013, 0.009) | 0.694 | 0.751 |
| Aβ42/40 | (r) caudalmiddlefronta volume | 0.000 (-0.009, 0.008) | 0.932 | 0.944 |
| Aβ42/40 | (r) cuneus volume | -0.010 (-0.020, 0.000) | 0.040 | 0.248 |
| Aβ42/40 | (r) entorhinal volume | -0.005 (-0.021, 0.011) | 0.555 | 0.643 |
| Aβ42/40 | (r) frontalpole volume | -0.011 (-0.032, 0.009) | 0.271 | 0.402 |
| Aβ42/40 | (r) fusiform volume | -0.010 (-0.018, -0.002) | 0.018 | 0.248 |
| Aβ42/40 | (r) inferiorparietal volume | -0.007 (-0.014, 0.000) | 0.043 | 0.248 |
| Aβ42/40 | (r) inferiortemporal volume | -0.001 (-0.008, 0.005) | 0.694 | 0.751 |
| Aβ42/40 | (r) insula volume | -0.005 (-0.020, 0.009) | 0.453 | 0.554 |
| Aβ42/40 | (r) isthmuscingulate volume | -0.005 (-0.013, 0.004) | 0.276 | 0.402 |
| Aβ42/40 | (r) lateraloccipital volume | -0.006 (-0.015, 0.003) | 0.179 | 0.349 |
| Aβ42/40 | (r) lateralorbitofrontal volume | -0.003 (-0.016, 0.010) | 0.680 | 0.751 |
| Aβ42/40 | (r) lingual volume | -0.007 (-0.017, 0.003) | 0.178 | 0.349 |
| Aβ42/40 | (r) medialorbitofrontal volume | -0.007 (-0.020, 0.005) | 0.231 | 0.383 |
| Aβ42/40 | (r) middletemporal volume | -0.010 (-0.018, -0.002) | 0.010 | 0.220 |
| Aβ42/40 | (r) paracentral volume | -0.016 (-0.027, -0.004) | 0.006 | 0.198 |
| Aβ42/40 | (r) parahippocampal volume | -0.014 (-0.029, 0.000) | 0.053 | 0.248 |
| Aβ42/40 | (r) parsopercularis volume | -0.005 (-0.013, 0.002) | 0.170 | 0.349 |
| Aβ42/40 | (r) parsorbitalis volume | -0.012 (-0.023, -0.001) | 0.031 | 0.248 |
| Aβ42/40 | (r) parstriangularis volume | -0.006 (-0.013, 0.002) | 0.155 | 0.349 |
| Aβ42/40 | (r) pericalcarine volume | -0.004 (-0.014, 0.005) | 0.388 | 0.507 |
| Aβ42/40 | (r) postcentral volume | -0.010 (-0.020, 0.000) | 0.055 | 0.248 |
| Aβ42/40 | (r) posteriorcingulate volume | -0.010 (-0.021, 0.000) | 0.060 | 0.248 |
| Aβ42/40 | (r) precentral volume | -0.007 (-0.019, 0.004) | 0.194 | 0.356 |
| Aβ42/40 | (r) precuneus volume | -0.006 (-0.014, 0.001) | 0.113 | 0.349 |
| Aβ42/40 | (r) rostralanteriorcingulate volume | -0.003 (-0.011, 0.006) | 0.541 | 0.638 |
| Aβ42/40 | (r) rostralmiddlefrontal volume | -0.006 (-0.013, 0.002) | 0.150 | 0.349 |
| Aβ42/40 | (r) superiorfrontal volume | -0.005 (-0.013, 0.004) | 0.276 | 0.402 |
| Aβ42/40 | (r) superiorparietal volume | -0.006 (-0.017, 0.005) | 0.280 | 0.402 |
| Aβ42/40 | (r) superiortemporal volume | -0.009 (-0.018, -0.001) | 0.026 | 0.248 |
| Aβ42/40 | (r) supramarginal volume | -0.005 (-0.013, 0.003) | 0.235 | 0.383 |
| Aβ42/40 | (r) transversetemporal volume | -0.005 (-0.016, 0.007) | 0.417 | 0.529 |
| GFAP | (l) bankssts volume | 0.007 (-0.001, 0.015) | 0.076 | 0.601 |
| GFAP | (l) caudalanteriorcingulate volume | 0.000 (-0.012, 0.012) | 0.961 | 0.983 |
| GFAP | (l) caudalmiddlefrontal volume | -0.005 (-0.013, 0.003) | 0.201 | 0.608 |
| GFAP | (l) cuneus volume | 0.007 (-0.002, 0.016) | 0.135 | 0.608 |
| GFAP | (l) entorhinal volume | -0.010 (-0.025, 0.005) | 0.204 | 0.608 |
| GFAP | (l) frontalpole volume | 0.001 (-0.018, 0.019) | 0.935 | 0.983 |
| GFAP | (l) fusiform volume | 0.012 (0.003, 0.020) | 0.009 | 0.528 |
| GFAP | (l) inferiorparietal volume | 0.004 (-0.003, 0.010) | 0.241 | 0.608 |
| GFAP | (l) inferiortemporal volume | 0.006 (-0.001, 0.013) | 0.101 | 0.606 |
| GFAP | (l) insula volume | -0.007 (-0.019, 0.006) | 0.290 | 0.608 |
| GFAP | (l) isthmuscingulate volume | 0.005 (-0.003, 0.013) | 0.205 | 0.608 |
| GFAP | (l) lateraloccipital volume | 0.004 (-0.005, 0.014) | 0.356 | 0.636 |
| GFAP | (l) lateralorbitofrontal volume | 0.005 (-0.004, 0.014) | 0.288 | 0.608 |
| GFAP | (l) lingual volume | 0.005 (-0.005, 0.014) | 0.366 | 0.636 |
| GFAP | (l) medialorbitofrontal volume | 0.015 (0.001, 0.030) | 0.042 | 0.594 |
| GFAP | (l) middletemporal volume | 0.004 (-0.004, 0.012) | 0.342 | 0.636 |
| GFAP | (l) paracentral volume | 0.010 (-0.001, 0.022) | 0.082 | 0.601 |
| GFAP | (l) parahippocampal volume | 0.000 (-0.013, 0.013) | 0.984 | 0.984 |
| GFAP | (l) parsopercularis volume | -0.002 (-0.008, 0.005) | 0.646 | 0.820 |
| GFAP | (l) parsorbitalis volume | 0.007 (-0.004, 0.018) | 0.194 | 0.608 |
| GFAP | (l) parstriangularis volume | 0.005 (-0.002, 0.012) | 0.162 | 0.608 |
| GFAP | (l) pericalcarine volume | 0.000 (-0.009, 0.008) | 0.943 | 0.983 |
| GFAP | (l) postcentral volume | 0.005 (-0.004, 0.015) | 0.291 | 0.608 |
| GFAP | (l) posteriorcingulate volume | 0.001 (-0.008, 0.011) | 0.799 | 0.922 |
| GFAP | (l) precentral volume | 0.006 (-0.004, 0.017) | 0.245 | 0.608 |
| GFAP | (l) precuneus volume | 0.002 (-0.005, 0.010) | 0.557 | 0.781 |
| GFAP | (l) rostralanteriorcingulate volume | 0.002 (-0.007, 0.011) | 0.660 | 0.822 |
| GFAP | (l) rostralmiddlefrontal volume | 0.002 (-0.005, 0.009) | 0.568 | 0.781 |
| GFAP | (l) superiorfrontal volume | 0.003 (-0.005, 0.011) | 0.480 | 0.720 |
| GFAP | (l) superiorparietal volume | 0.004 (-0.004, 0.013) | 0.332 | 0.636 |
| GFAP | (l) superiortemporal volume | 0.005 (-0.003, 0.013) | 0.217 | 0.608 |
| GFAP | (l) supramarginal volume | 0.001 (-0.005, 0.007) | 0.731 | 0.893 |
| GFAP | (l) transversetemporal volume | -0.002 (-0.011, 0.007) | 0.629 | 0.820 |
| GFAP | (r) bankssts volume | -0.004 (-0.012, 0.005) | 0.381 | 0.645 |
| GFAP | (r) caudalanteriorcingulate volume | -0.001 (-0.012, 0.011) | 0.911 | 0.983 |
| GFAP | (r) caudalmiddlefronta volume | -0.010 (-0.019, -0.002) | 0.016 | 0.528 |
| GFAP | (r) cuneus volume | -0.006 (-0.016, 0.003) | 0.194 | 0.608 |
| GFAP | (r) entorhinal volume | 0.001 (-0.016, 0.017) | 0.943 | 0.983 |
| GFAP | (r) frontalpole volume | -0.006 (-0.026, 0.015) | 0.587 | 0.791 |
| GFAP | (r) fusiform volume | -0.005 (-0.014, 0.003) | 0.206 | 0.608 |
| GFAP | (r) inferiorparietal volume | -0.004 (-0.011, 0.003) | 0.295 | 0.608 |
| GFAP | (r) inferiortemporal volume | -0.004 (-0.011, 0.003) | 0.235 | 0.608 |
| GFAP | (r) insula volume | -0.014 (-0.028, 0.001) | 0.064 | 0.601 |
| GFAP | (r) isthmuscingulate volume | -0.002 (-0.011, 0.007) | 0.635 | 0.820 |
| GFAP | (r) lateraloccipital volume | -0.004 (-0.013, 0.005) | 0.360 | 0.636 |
| GFAP | (r) lateralorbitofrontal volume | -0.006 (-0.019, 0.008) | 0.406 | 0.654 |
| GFAP | (r) lingual volume | -0.011 (-0.021, 0.000) | 0.042 | 0.594 |
| GFAP | (r) medialorbitofrontal volume | 0.004 (-0.008, 0.016) | 0.519 | 0.745 |
| GFAP | (r) middletemporal volume | 0.000 (-0.008, 0.008) | 0.968 | 0.983 |
| GFAP | (r) paracentral volume | 0.006 (-0.005, 0.017) | 0.323 | 0.636 |
| GFAP | (r) parahippocampal volume | -0.001 (-0.016, 0.013) | 0.851 | 0.952 |
| GFAP | (r) parsopercularis volume | -0.008 (-0.016, 0.000) | 0.048 | 0.594 |
| GFAP | (r) parsorbitalis volume | -0.002 (-0.013, 0.010) | 0.775 | 0.913 |
| GFAP | (r) parstriangularis volume | -0.003 (-0.011, 0.005) | 0.508 | 0.745 |
| GFAP | (r) pericalcarine volume | -0.007 (-0.016, 0.003) | 0.176 | 0.608 |
| GFAP | (r) postcentral volume | -0.008 (-0.018, 0.002) | 0.127 | 0.608 |
| GFAP | (r) posteriorcingulate volume | 0.002 (-0.009, 0.012) | 0.762 | 0.913 |
| GFAP | (r) precentral volume | -0.006 (-0.018, 0.005) | 0.278 | 0.608 |
| GFAP | (r) precuneus volume | 0.001 (-0.007, 0.008) | 0.810 | 0.922 |
| GFAP | (r) rostralanteriorcingulate volume | -0.003 (-0.012, 0.005) | 0.453 | 0.695 |
| GFAP | (r) rostralmiddlefrontal volume | -0.007 (-0.014, 0.001) | 0.091 | 0.601 |
| GFAP | (r) superiorfrontal volume | -0.006 (-0.014, 0.003) | 0.176 | 0.608 |
| GFAP | (r) superiorparietal volume | -0.005 (-0.015, 0.006) | 0.403 | 0.654 |
| GFAP | (r) superiortemporal volume | -0.005 (-0.013, 0.004) | 0.265 | 0.608 |
| GFAP | (r) supramarginal volume | -0.003 (-0.011, 0.005) | 0.439 | 0.690 |
| GFAP | (r) transversetemporal volume | -0.011 (-0.023, 0.000) | 0.054 | 0.594 |
| NfL | (l) bankssts volume | -0.003 (-0.009, 0.004) | 0.439 | 0.957 |
| NfL | (l) caudalanteriorcingulate volume | -0.005 (-0.015, 0.005) | 0.306 | 0.957 |
| NfL | (l) caudalmiddlefrontal volume | -0.004 (-0.011, 0.002) | 0.216 | 0.957 |
| NfL | (l) cuneus volume | 0.006 (-0.002, 0.014) | 0.151 | 0.957 |
| NfL | (l) entorhinal volume | -0.010 (-0.023, 0.003) | 0.136 | 0.957 |
| NfL | (l) frontalpole volume | 0.002 (-0.013, 0.018) | 0.778 | 0.957 |
| NfL | (l) fusiform volume | 0.002 (-0.005, 0.009) | 0.603 | 0.957 |
| NfL | (l) inferiorparietal volume | 0.001 (-0.004, 0.007) | 0.697 | 0.957 |
| NfL | (l) inferiortemporal volume | 0.000 (-0.006, 0.006) | 0.943 | 0.972 |
| NfL | (l) insula volume | -0.008 (-0.019, 0.002) | 0.122 | 0.957 |
| NfL | (l) isthmuscingulate volume | 0.004 (-0.002, 0.011) | 0.210 | 0.957 |
| NfL | (l) lateraloccipital volume | 0.002 (-0.006, 0.010) | 0.618 | 0.957 |
| NfL | (l) lateralorbitofrontal volume | -0.002 (-0.010, 0.005) | 0.549 | 0.957 |
| NfL | (l) lingual volume | 0.001 (-0.007, 0.009) | 0.802 | 0.957 |
| NfL | (l) medialorbitofrontal volume | 0.007 (-0.005, 0.020) | 0.265 | 0.957 |
| NfL | (l) middletemporal volume | -0.001 (-0.008, 0.006) | 0.812 | 0.957 |
| NfL | (l) paracentral volume | 0.002 (-0.008, 0.012) | 0.663 | 0.957 |
| NfL | (l) parahippocampal volume | -0.004 (-0.015, 0.007) | 0.463 | 0.957 |
| NfL | (l) parsopercularis volume | -0.008 (-0.015, -0.002) | 0.009 | 0.594 |
| NfL | (l) parsorbitalis volume | 0.001 (-0.008, 0.011) | 0.802 | 0.957 |
| NfL | (l) parstriangularis volume | -0.005 (-0.011, 0.001) | 0.104 | 0.957 |
| NfL | (l) pericalcarine volume | 0.000 (-0.007, 0.007) | 0.998 | 0.998 |
| NfL | (l) postcentral volume | 0.001 (-0.007, 0.009) | 0.812 | 0.957 |
| NfL | (l) posteriorcingulate volume | -0.003 (-0.012, 0.005) | 0.415 | 0.957 |
| NfL | (l) precentral volume | 0.005 (-0.004, 0.015) | 0.273 | 0.957 |
| NfL | (l) precuneus volume | 0.001 (-0.005, 0.008) | 0.662 | 0.957 |
| NfL | (l) rostralanteriorcingulate volume | -0.001 (-0.009, 0.006) | 0.712 | 0.957 |
| NfL | (l) rostralmiddlefrontal volume | -0.008 (-0.014, -0.001) | 0.022 | 0.726 |
| NfL | (l) superiorfrontal volume | -0.002 (-0.009, 0.005) | 0.546 | 0.957 |
| NfL | (l) superiorparietal volume | 0.006 (-0.001, 0.014) | 0.113 | 0.957 |
| NfL | (l) superiortemporal volume | -0.001 (-0.008, 0.006) | 0.777 | 0.957 |
| NfL | (l) supramarginal volume | 0.003 (-0.003, 0.008) | 0.358 | 0.957 |
| NfL | (l) transversetemporal volume | -0.001 (-0.008, 0.006) | 0.771 | 0.957 |
| NfL | (r) bankssts volume | -0.003 (-0.010, 0.004) | 0.390 | 0.957 |
| NfL | (r) caudalanteriorcingulate volume | -0.001 (-0.011, 0.008) | 0.774 | 0.957 |
| NfL | (r) caudalmiddlefronta volume | 0.003 (-0.004, 0.011) | 0.378 | 0.957 |
| NfL | (r) cuneus volume | 0.003 (-0.005, 0.011) | 0.498 | 0.957 |
| NfL | (r) entorhinal volume | -0.008 (-0.022, 0.006) | 0.244 | 0.957 |
| NfL | (r) frontalpole volume | 0.007 (-0.010, 0.024) | 0.415 | 0.957 |
| NfL | (r) fusiform volume | -0.001 (-0.008, 0.006) | 0.779 | 0.957 |
| NfL | (r) inferiorparietal volume | -0.001 (-0.007, 0.005) | 0.838 | 0.964 |
| NfL | (r) inferiortemporal volume | -0.002 (-0.008, 0.003) | 0.416 | 0.957 |
| NfL | (r) insula volume | -0.006 (-0.019, 0.006) | 0.300 | 0.957 |
| NfL | (r) isthmuscingulate volume | 0.000 (-0.007, 0.008) | 0.915 | 0.970 |
| NfL | (r) lateraloccipital volume | -0.002 (-0.009, 0.006) | 0.658 | 0.957 |
| NfL | (r) lateralorbitofrontal volume | -0.005 (-0.016, 0.006) | 0.389 | 0.957 |
| NfL | (r) lingual volume | 0.002 (-0.007, 0.011) | 0.668 | 0.957 |
| NfL | (r) medialorbitofrontal volume | 0.007 (-0.003, 0.017) | 0.191 | 0.957 |
| NfL | (r) middletemporal volume | -0.002 (-0.008, 0.005) | 0.635 | 0.957 |
| NfL | (r) paracentral volume | 0.004 (-0.006, 0.013) | 0.416 | 0.957 |
| NfL | (r) parahippocampal volume | -0.001 (-0.014, 0.011) | 0.852 | 0.964 |
| NfL | (r) parsopercularis volume | 0.002 (-0.005, 0.009) | 0.526 | 0.957 |
| NfL | (r) parsorbitalis volume | 0.000 (-0.010, 0.009) | 0.977 | 0.992 |
| NfL | (r) parstriangularis volume | -0.005 (-0.012, 0.002) | 0.156 | 0.957 |
| NfL | (r) pericalcarine volume | 0.001 (-0.007, 0.009) | 0.871 | 0.964 |
| NfL | (r) postcentral volume | 0.000 (-0.009, 0.008) | 0.926 | 0.970 |
| NfL | (r) posteriorcingulate volume | -0.001 (-0.010, 0.008) | 0.876 | 0.964 |
| NfL | (r) precentral volume | 0.003 (-0.007, 0.012) | 0.581 | 0.957 |
| NfL | (r) precuneus volume | -0.001 (-0.008, 0.005) | 0.695 | 0.957 |
| NfL | (r) rostralanteriorcingulate volume | -0.005 (-0.012, 0.002) | 0.176 | 0.957 |
| NfL | (r) rostralmiddlefrontal volume | -0.003 (-0.009, 0.004) | 0.430 | 0.957 |
| NfL | (r) superiorfrontal volume | -0.002 (-0.009, 0.006) | 0.633 | 0.957 |
| NfL | (r) superiorparietal volume | 0.002 (-0.008, 0.011) | 0.715 | 0.957 |
| NfL | (r) superiortemporal volume | -0.001 (-0.008, 0.006) | 0.713 | 0.957 |
| NfL | (r) supramarginal volume | 0.000 (-0.007, 0.008) | 0.909 | 0.970 |
| NfL | (r) transversetemporal volume | 0.007 (-0.003, 0.017) | 0.149 | 0.957 |
| p-tau181 | (l) bankssts volume | -0.006 (-0.013, 0.002) | 0.121 | 0.705 |
| p-tau181 | (l) caudalanteriorcingulate volume | -0.008 (-0.019, 0.004) | 0.200 | 0.705 |
| p-tau181 | (l) caudalmiddlefrontal volume | 0.003 (-0.005, 0.010) | 0.504 | 0.875 |
| p-tau181 | (l) cuneus volume | -0.002 (-0.011, 0.007) | 0.663 | 0.942 |
| p-tau181 | (l) entorhinal volume | 0.010 (-0.005, 0.025) | 0.193 | 0.705 |
| p-tau181 | (l) frontalpole volume | 0.006 (-0.012, 0.024) | 0.497 | 0.875 |
| p-tau181 | (l) fusiform volume | -0.007 (-0.015, 0.002) | 0.109 | 0.705 |
| p-tau181 | (l) inferiorparietal volume | 0.002 (-0.005, 0.008) | 0.634 | 0.942 |
| p-tau181 | (l) inferiortemporal volume | -0.002 (-0.009, 0.004) | 0.485 | 0.875 |
| p-tau181 | (l) insula volume | 0.000 (-0.012, 0.013) | 0.975 | 0.991 |
| p-tau181 | (l) isthmuscingulate volume | -0.001 (-0.009, 0.007) | 0.776 | 0.958 |
| p-tau181 | (l) lateraloccipital volume | 0.001 (-0.008, 0.010) | 0.818 | 0.964 |
| p-tau181 | (l) lateralorbitofrontal volume | -0.003 (-0.012, 0.005) | 0.462 | 0.875 |
| p-tau181 | (l) lingual volume | 0.004 (-0.006, 0.014) | 0.418 | 0.875 |
| p-tau181 | (l) medialorbitofrontal volume | 0.001 (-0.014, 0.015) | 0.906 | 0.964 |
| p-tau181 | (l) middletemporal volume | 0.000 (-0.008, 0.008) | 0.984 | 0.991 |
| p-tau181 | (l) paracentral volume | 0.000 (-0.011, 0.011) | 0.991 | 0.991 |
| p-tau181 | (l) parahippocampal volume | -0.002 (-0.015, 0.010) | 0.707 | 0.952 |
| p-tau181 | (l) parsopercularis volume | 0.005 (-0.002, 0.012) | 0.178 | 0.705 |
| p-tau181 | (l) parsorbitalis volume | 0.004 (-0.007, 0.015) | 0.470 | 0.875 |
| p-tau181 | (l) parstriangularis volume | 0.001 (-0.006, 0.007) | 0.860 | 0.964 |
| p-tau181 | (l) pericalcarine volume | 0.004 (-0.004, 0.012) | 0.356 | 0.875 |
| p-tau181 | (l) postcentral volume | -0.001 (-0.010, 0.009) | 0.892 | 0.964 |
| p-tau181 | (l) posteriorcingulate volume | -0.005 (-0.014, 0.005) | 0.354 | 0.875 |
| p-tau181 | (l) precentral volume | 0.002 (-0.008, 0.013) | 0.648 | 0.942 |
| p-tau181 | (l) precuneus volume | 0.000 (-0.008, 0.007) | 0.979 | 0.991 |
| p-tau181 | (l) rostralanteriorcingulate volume | -0.010 (-0.019, -0.002) | 0.018 | 0.396 |
| p-tau181 | (l) rostralmiddlefrontal volume | 0.000 (-0.008, 0.007) | 0.896 | 0.964 |
| p-tau181 | (l) superiorfrontal volume | 0.005 (-0.004, 0.013) | 0.267 | 0.766 |
| p-tau181 | (l) superiorparietal volume | -0.001 (-0.010, 0.008) | 0.766 | 0.958 |
| p-tau181 | (l) superiortemporal volume | -0.002 (-0.010, 0.006) | 0.583 | 0.916 |
| p-tau181 | (l) supramarginal volume | -0.002 (-0.008, 0.004) | 0.472 | 0.875 |
| p-tau181 | (l) transversetemporal volume | 0.007 (-0.001, 0.016) | 0.086 | 0.631 |
| p-tau181 | (r) bankssts volume | -0.001 (-0.009, 0.007) | 0.812 | 0.964 |
| p-tau181 | (r) caudalanteriorcingulate volume | 0.004 (-0.007, 0.015) | 0.450 | 0.875 |
| p-tau181 | (r) caudalmiddlefronta volume | 0.008 (-0.001, 0.017) | 0.074 | 0.631 |
| p-tau181 | (r) cuneus volume | 0.003 (-0.007, 0.012) | 0.575 | 0.916 |
| p-tau181 | (r) entorhinal volume | 0.007 (-0.009, 0.023) | 0.403 | 0.875 |
| p-tau181 | (r) frontalpole volume | -0.005 (-0.024, 0.015) | 0.647 | 0.942 |
| p-tau181 | (r) fusiform volume | 0.003 (-0.006, 0.011) | 0.535 | 0.905 |
| p-tau181 | (r) inferiorparietal volume | 0.004 (-0.002, 0.011) | 0.203 | 0.705 |
| p-tau181 | (r) inferiortemporal volume | -0.001 (-0.007, 0.006) | 0.856 | 0.964 |
| p-tau181 | (r) insula volume | 0.003 (-0.011, 0.017) | 0.671 | 0.942 |
| p-tau181 | (r) isthmuscingulate volume | 0.006 (-0.003, 0.014) | 0.172 | 0.705 |
| p-tau181 | (r) lateraloccipital volume | 0.011 (0.002, 0.019) | 0.016 | 0.396 |
| p-tau181 | (r) lateralorbitofrontal volume | -0.004 (-0.017, 0.009) | 0.550 | 0.908 |
| p-tau181 | (r) lingual volume | 0.009 (-0.001, 0.019) | 0.079 | 0.631 |
| p-tau181 | (r) medialorbitofrontal volume | 0.009 (-0.003, 0.021) | 0.165 | 0.705 |
| p-tau181 | (r) middletemporal volume | 0.004 (-0.003, 0.012) | 0.252 | 0.759 |
| p-tau181 | (r) paracentral volume | 0.002 (-0.009, 0.013) | 0.772 | 0.958 |
| p-tau181 | (r) parahippocampal volume | -0.003 (-0.017, 0.012) | 0.705 | 0.952 |
| p-tau181 | (r) parsopercularis volume | 0.005 (-0.002, 0.013) | 0.183 | 0.705 |
| p-tau181 | (r) parsorbitalis volume | 0.002 (-0.009, 0.012) | 0.784 | 0.958 |
| p-tau181 | (r) parstriangularis volume | 0.001 (-0.007, 0.008) | 0.876 | 0.964 |
| p-tau181 | (r) pericalcarine volume | 0.004 (-0.005, 0.013) | 0.379 | 0.875 |
| p-tau181 | (r) postcentral volume | 0.010 (0.000, 0.020) | 0.051 | 0.631 |
| p-tau181 | (r) posteriorcingulate volume | 0.013 (0.002, 0.023) | 0.016 | 0.396 |
| p-tau181 | (r) precentral volume | 0.008 (-0.003, 0.019) | 0.156 | 0.705 |
| p-tau181 | (r) precuneus volume | 0.003 (-0.005, 0.011) | 0.447 | 0.875 |
| p-tau181 | (r) rostralanteriorcingulate volume | 0.001 (-0.007, 0.010) | 0.733 | 0.958 |
| p-tau181 | (r) rostralmiddlefrontal volume | 0.005 (-0.003, 0.012) | 0.234 | 0.759 |
| p-tau181 | (r) superiorfrontal volume | 0.008 (-0.001, 0.016) | 0.066 | 0.631 |
| p-tau181 | (r) superiorparietal volume | 0.011 (0.001, 0.022) | 0.036 | 0.594 |
| p-tau181 | (r) superiortemporal volume | 0.004 (-0.005, 0.012) | 0.391 | 0.875 |
| p-tau181 | (r) supramarginal volume | 0.005 (-0.003, 0.013) | 0.253 | 0.759 |
| p-tau181 | (r) transversetemporal volume | -0.004 (-0.015, 0.007) | 0.468 | 0.875 |

Abbreviations: Aβ, amyloid-β; GFAP, glial fibrillary acidic protein; NfL, neurofilament light chain; p-tau181, tau phosphorylated at threonine 181; CI, confidence interval; l, left hemisphere; r, right hemisphere; FDR, false discovery rates.

Model was adjusted for sex, age, ethnicity, townsend deprivation index, assessment center, smoking status, alcohol use, family history of dementia, *APOE* ε4 allele, hypertension, diabetes, cardiovascular arterial disease, history of COVID-19 infection, and total intracranial volume.

The brain regions are as defined in the Desikan-Killiany atlas (cortex).

# Supplementary Table 13. Associations of longitudinal plasma AD-related biomarkers with longitudinal changes in subcortical brain volumes by using linear mixed-effects models.

| **Plasma biomarkers** | **Subcortical brain volumes** | **β (95% CI)** | ***P*-value** | ***FDR-***  ***corrected P*** |
| --- | --- | --- | --- | --- |
| Aβ42/40 | Right_Thalamus_Proper | 0.006 (-0.001, 0.012) | 0.090 | 0.360 |
| Aβ42/40 | Right_Putamen | 0.001 (-0.006, 0.007) | 0.847 | 0.917 |
| Aβ42/40 | Right_Pallidum | 0.000 (-0.008, 0.008) | 0.962 | 0.962 |
| Aβ42/40 | Right_Lateral_Ventricle | -0.002 (-0.004, 0.001) | 0.291 | 0.723 |
| Aβ42/40 | Right_Hippocampus | 0.009 (0.002, 0.016) | 0.010 | 0.160 |
| Aβ42/40 | Right_Caudate | 0.001 (-0.006, 0.008) | 0.786 | 0.917 |
| Aβ42/40 | Right_Amygdala | 0.004 (-0.004, 0.013) | 0.336 | 0.723 |
| Aβ42/40 | Right_Accumbens_area | 0.003 (-0.006, 0.013) | 0.487 | 0.723 |
| Aβ42/40 | Left_Thalamus_Proper | -0.001 (-0.009, 0.007) | 0.770 | 0.917 |
| Aβ42/40 | Left_Putamen | 0.002 (-0.004, 0.009) | 0.461 | 0.723 |
| Aβ42/40 | Left_Pallidum | 0.003 (-0.006, 0.012) | 0.497 | 0.723 |
| Aβ42/40 | Left_Lateral_Ventricle | -0.002 (-0.004, 0.001) | 0.267 | 0.723 |
| Aβ42/40 | Left_Hippocampus | 0.006 (-0.001, 0.013) | 0.081 | 0.360 |
| Aβ42/40 | Left_Caudate | 0.002 (-0.003, 0.007) | 0.450 | 0.723 |
| Aβ42/40 | Left_Amygdala | 0.008 (-0.001, 0.018) | 0.084 | 0.360 |
| Aβ42/40 | Left_Accumbens_area | -0.001 (-0.012, 0.010) | 0.860 | 0.917 |
| GFAP | Right_Thalamus_Proper | -0.003 (-0.011, 0.006) | 0.550 | 0.983 |
| GFAP | Right_Putamen | 0.001 (-0.006, 0.009) | 0.749 | 0.983 |
| GFAP | Right_Pallidum | 0.000 (-0.010, 0.010) | 0.994 | 0.994 |
| GFAP | Right_Lateral_Ventricle | 0.000 (-0.004, 0.004) | 0.922 | 0.983 |
| GFAP | Right_Hippocampus | -0.005 (-0.014, 0.003) | 0.236 | 0.983 |
| GFAP | Right_Caudate | 0.003 (-0.005, 0.012) | 0.446 | 0.983 |
| GFAP | Right_Amygdala | 0.001 (-0.010, 0.012) | 0.891 | 0.983 |
| GFAP | Right_Accumbens_area | 0.006 (-0.006, 0.019) | 0.333 | 0.983 |
| GFAP | Left_Thalamus_Proper | 0.001 (-0.009, 0.011) | 0.849 | 0.983 |
| GFAP | Left_Putamen | 0.003 (-0.005, 0.011) | 0.442 | 0.983 |
| GFAP | Left_Pallidum | 0.001 (-0.011, 0.013) | 0.879 | 0.983 |
| GFAP | Left_Lateral_Ventricle | 0.000 (-0.004, 0.003) | 0.919 | 0.983 |
| GFAP | Left_Hippocampus | 0.001 (-0.008, 0.010) | 0.869 | 0.983 |
| GFAP | Left_Caudate | 0.003 (-0.004, 0.010) | 0.362 | 0.983 |
| GFAP | Left_Amygdala | 0.008 (-0.004, 0.021) | 0.197 | 0.983 |
| GFAP | Left_Accumbens_area | 0.005 (-0.009, 0.019) | 0.490 | 0.983 |
| NfL | Right_Thalamus_Proper | -0.006 (-0.013, 0.001) | 0.091 | 0.712 |
| NfL | Right_Putamen | 0.002 (-0.005, 0.009) | 0.542 | 0.964 |
| NfL | Right_Pallidum | -0.002 (-0.010, 0.007) | 0.735 | 0.965 |
| NfL | Right_Lateral_Ventricle | 0.003 (0.000, 0.006) | 0.052 | 0.712 |
| NfL | Right_Hippocampus | -0.004 (-0.011, 0.003) | 0.273 | 0.874 |
| NfL | Right_Caudate | -0.001 (-0.008, 0.006) | 0.793 | 0.965 |
| NfL | Right_Amygdala | 0.004 (-0.006, 0.013) | 0.429 | 0.942 |
| NfL | Right_Accumbens_area | -0.001 (-0.011, 0.010) | 0.905 | 0.965 |
| NfL | Left_Thalamus_Proper | 0.002 (-0.007, 0.011) | 0.641 | 0.965 |
| NfL | Left_Putamen | 0.000 (-0.007, 0.007) | 0.902 | 0.965 |
| NfL | Left_Pallidum | -0.002 (-0.012, 0.008) | 0.736 | 0.965 |
| NfL | Left_Lateral_Ventricle | 0.001 (-0.002, 0.004) | 0.379 | 0.942 |
| NfL | Left_Hippocampus | -0.005 (-0.013, 0.002) | 0.163 | 0.712 |
| NfL | Left_Caudate | 0.002 (-0.004, 0.008) | 0.471 | 0.942 |
| NfL | Left_Amygdala | 0.007 (-0.003, 0.018) | 0.178 | 0.712 |
| NfL | Left_Accumbens_area | 0.000 (-0.012, 0.012) | 0.979 | 0.979 |
| p-tau181 | Right_Thalamus_Proper | 0.004 (-0.004, 0.012) | 0.349 | 0.754 |
| p-tau181 | Right_Putamen | -0.003 (-0.011, 0.005) | 0.494 | 0.754 |
| p-tau181 | Right_Pallidum | -0.005 (-0.015, 0.005) | 0.342 | 0.754 |
| p-tau181 | Right_Lateral_Ventricle | 0.001 (-0.003, 0.005) | 0.613 | 0.754 |
| p-tau181 | Right_Hippocampus | 0.002 (-0.007, 0.010) | 0.721 | 0.769 |
| p-tau181 | Right_Caudate | 0.002 (-0.007, 0.010) | 0.667 | 0.762 |
| p-tau181 | Right_Amygdala | 0.006 (-0.005, 0.016) | 0.285 | 0.754 |
| p-tau181 | Right_Accumbens_area | 0.003 (-0.009, 0.016) | 0.594 | 0.754 |
| p-tau181 | Left_Thalamus_Proper | 0.008 (-0.002, 0.018) | 0.115 | 0.754 |
| p-tau181 | Left_Putamen | 0.003 (-0.005, 0.011) | 0.540 | 0.754 |
| p-tau181 | Left_Pallidum | -0.003 (-0.014, 0.008) | 0.589 | 0.754 |
| p-tau181 | Left_Lateral_Ventricle | 0.002 (-0.002, 0.005) | 0.382 | 0.754 |
| p-tau181 | Left_Hippocampus | -0.001 (-0.010, 0.008) | 0.838 | 0.838 |
| p-tau181 | Left_Caudate | 0.003 (-0.004, 0.010) | 0.394 | 0.754 |
| p-tau181 | Left_Amygdala | -0.007 (-0.020, 0.005) | 0.234 | 0.754 |
| p-tau181 | Left_Accumbens_area | 0.004 (-0.010, 0.018) | 0.581 | 0.754 |

Abbreviations: Aβ, amyloid-β; GFAP, glial fibrillary acidic protein; NfL, neurofilament light chain; p-tau181, tau phosphorylated at threonine 181; CI, confidence interval; FDR, false discovery rates.

Model was adjusted for sex, age, ethnicity, townsend deprivation index, assessment center, smoking status, alcohol use, family history of dementia, *APOE* ε4 allele, hypertension, diabetes, cardiovascular arterial disease, history of COVID-19 infection, and total intracranial volume.

The brain regions are as defined in the ASEG atlas (subcortex).

# Supplementary Table 14. Associations of baseline plasma AD-related biomarkers with longitudinal changes in white matter microstructure by using linear mixed-effects models.

| **Plasma biomarkers** | **White matter microstructure** | **β (95% CI)** | ***P*-value** | ***FDR-***  ***corrected P*** |
| --- | --- | --- | --- | --- |
| Aβ42/40 | uncinate fasciculus (r) FA | 0.009 (-0.004, 0.022) | 0.185 | 0.991 |
| Aβ42/40 | uncinate fasciculus (l) FA | -0.002 (-0.016, 0.011) | 0.755 | 0.991 |
| Aβ42/40 | superior thalamic radiation (r) FA | -0.005 (-0.013, 0.004) | 0.285 | 0.991 |
| Aβ42/40 | superior thalamic radiation (l) FA | -0.003 (-0.011, 0.005) | 0.413 | 0.991 |
| Aβ42/40 | superior longitudinal fasciculus (r) FA | 0.000 (-0.007, 0.007) | 0.984 | 0.991 |
| Aβ42/40 | superior longitudinal fasciculus (l) FA | 0.000 (-0.008, 0.007) | 0.944 | 0.991 |
| Aβ42/40 | posterior thalamic radiation (r) FA | -0.003 (-0.014, 0.008) | 0.556 | 0.991 |
| Aβ42/40 | posterior thalamic radiation (l) FA | 0.003 (-0.008, 0.014) | 0.643 | 0.991 |
| Aβ42/40 | parahippocampal part of cingulum (r) FA | 0.000 (-0.014, 0.015) | 0.970 | 0.991 |
| Aβ42/40 | parahippocampal part of cingulum (l) FA | -0.006 (-0.022, 0.009) | 0.426 | 0.991 |
| Aβ42/40 | middle cerebellar peduncle (l) FA | -0.004 (-0.018, 0.010) | 0.554 | 0.991 |
| Aβ42/40 | medial lemniscus (r) FA | -0.016 (-0.031, 0.000) | 0.049 | 0.991 |
| Aβ42/40 | medial lemniscus (l) FA | -0.004 (-0.019, 0.011) | 0.622 | 0.991 |
| Aβ42/40 | inferior longitudinal fasciculus (r) FA | 0.001 (-0.008, 0.009) | 0.882 | 0.991 |
| Aβ42/40 | inferior longitudinal fasciculus (l) FA | 0.000 (-0.009, 0.009) | 0.966 | 0.991 |
| Aβ42/40 | inferior fronto-occipital fasciculus (r) FA | -0.007 (-0.016, 0.001) | 0.091 | 0.991 |
| Aβ42/40 | inferior fronto-occipital fasciculus (l) FA | -0.001 (-0.011, 0.008) | 0.756 | 0.991 |
| Aβ42/40 | forceps major (r) FA | 0.004 (-0.004, 0.012) | 0.302 | 0.991 |
| Aβ42/40 | forceps major (l) FA | -0.002 (-0.011, 0.008) | 0.731 | 0.991 |
| Aβ42/40 | corticospinal tract (r) FA | -0.010 (-0.022, 0.001) | 0.078 | 0.991 |
| Aβ42/40 | corticospinal tract (l) FA | -0.005 (-0.016, 0.007) | 0.428 | 0.991 |
| Aβ42/40 | cingulate gyrus part of cingulum (r) FA | 0.002 (-0.008, 0.012) | 0.708 | 0.991 |
| Aβ42/40 | cingulate gyrus part of cingulum (l) FA | -0.001 (-0.012, 0.011) | 0.881 | 0.991 |
| Aβ42/40 | anterior thalamic radiation (r) FA | 0.000 (-0.010, 0.009) | 0.929 | 0.991 |
| Aβ42/40 | anterior thalamic radiation (l) FA | -0.003 (-0.013, 0.007) | 0.565 | 0.991 |
| Aβ42/40 | acoustic radiation (r) FA | -0.002 (-0.015, 0.011) | 0.756 | 0.991 |
| Aβ42/40 | acoustic radiation (l) FA | 0.005 (-0.009, 0.018) | 0.474 | 0.991 |
| GFAP | uncinate fasciculus (r) FA | -0.004 (-0.016, 0.008) | 0.501 | 0.689 |
| GFAP | uncinate fasciculus (l) FA | -0.005 (-0.018, 0.008) | 0.458 | 0.651 |
| GFAP | superior thalamic radiation (r) FA | 0.011 (0.003, 0.019) | 0.005 | 0.068 |
| GFAP | superior thalamic radiation (l) FA | 0.009 (0.001, 0.016) | 0.025 | 0.178 |
| GFAP | superior longitudinal fasciculus (r) FA | -0.001 (-0.008, 0.006) | 0.749 | 0.891 |
| GFAP | superior longitudinal fasciculus (l) FA | -0.007 (-0.014, 0.000) | 0.058 | 0.218 |
| GFAP | posterior thalamic radiation (r) FA | 0.014 (0.003, 0.024) | 0.011 | 0.108 |
| GFAP | posterior thalamic radiation (l) FA | -0.011 (-0.022, -0.001) | 0.035 | 0.187 |
| GFAP | parahippocampal part of cingulum (r) FA | 0.005 (-0.008, 0.019) | 0.432 | 0.630 |
| GFAP | parahippocampal part of cingulum (l) FA | 0.007 (-0.007, 0.022) | 0.327 | 0.565 |
| GFAP | middle cerebellar peduncle (l) FA | 0.002 (-0.011, 0.016) | 0.723 | 0.891 |
| GFAP | medial lemniscus (r) FA | 0.007 (-0.007, 0.022) | 0.333 | 0.565 |
| GFAP | medial lemniscus (l) FA | -0.004 (-0.019, 0.010) | 0.535 | 0.708 |
| GFAP | inferior longitudinal fasciculus (r) FA | 0.001 (-0.007, 0.009) | 0.829 | 0.917 |
| GFAP | inferior longitudinal fasciculus (l) FA | -0.004 (-0.012, 0.004) | 0.364 | 0.565 |
| GFAP | inferior fronto-occipital fasciculus (r) FA | 0.004 (-0.004, 0.012) | 0.363 | 0.565 |
| GFAP | inferior fronto-occipital fasciculus (l) FA | -0.007 (-0.016, 0.002) | 0.133 | 0.359 |
| GFAP | forceps major (r) FA | 0.001 (-0.007, 0.009) | 0.779 | 0.891 |
| GFAP | forceps major (l) FA | -0.010 (-0.019, -0.001) | 0.023 | 0.173 |
| GFAP | corticospinal tract (r) FA | 0.008 (-0.003, 0.018) | 0.158 | 0.388 |
| GFAP | corticospinal tract (l) FA | -0.004 (-0.015, 0.007) | 0.479 | 0.674 |
| GFAP | cingulate gyrus part of cingulum (r) FA | 0.001 (-0.009, 0.011) | 0.853 | 0.917 |
| GFAP | cingulate gyrus part of cingulum (l) FA | -0.001 (-0.012, 0.010) | 0.863 | 0.917 |
| GFAP | anterior thalamic radiation (r) FA | 0.012 (0.003, 0.021) | 0.007 | 0.079 |
| GFAP | anterior thalamic radiation (l) FA | 0.001 (-0.008, 0.011) | 0.785 | 0.891 |
| GFAP | acoustic radiation (r) FA | 0.007 (-0.005, 0.019) | 0.253 | 0.531 |
| GFAP | acoustic radiation (l) FA | 0.008 (-0.005, 0.020) | 0.231 | 0.518 |
| NfL | uncinate fasciculus (r) FA | -0.002 (-0.014, 0.010) | 0.751 | 0.818 |
| NfL | uncinate fasciculus (l) FA | 0.005 (-0.008, 0.017) | 0.445 | 0.630 |
| NfL | superior thalamic radiation (r) FA | 0.009 (0.001, 0.016) | 0.021 | 0.095 |
| NfL | superior thalamic radiation (l) FA | 0.012 (0.005, 0.020) | 0.001 | 0.023 |
| NfL | superior longitudinal fasciculus (r) FA | -0.002 (-0.008, 0.005) | 0.657 | 0.784 |
| NfL | superior longitudinal fasciculus (l) FA | -0.003 (-0.010, 0.004) | 0.450 | 0.630 |
| NfL | posterior thalamic radiation (r) FA | 0.002 (-0.008, 0.012) | 0.740 | 0.812 |
| NfL | posterior thalamic radiation (l) FA | -0.009 (-0.019, 0.001) | 0.082 | 0.221 |
| NfL | parahippocampal part of cingulum (r) FA | 0.001 (-0.012, 0.014) | 0.877 | 0.925 |
| NfL | parahippocampal part of cingulum (l) FA | 0.008 (-0.006, 0.023) | 0.262 | 0.463 |
| NfL | middle cerebellar peduncle (l) FA | -0.008 (-0.021, 0.005) | 0.215 | 0.409 |
| NfL | medial lemniscus (r) FA | -0.008 (-0.022, 0.006) | 0.281 | 0.478 |
| NfL | medial lemniscus (l) FA | -0.016 (-0.030, -0.002) | 0.022 | 0.096 |
| NfL | inferior longitudinal fasciculus (r) FA | -0.003 (-0.011, 0.005) | 0.478 | 0.638 |
| NfL | inferior longitudinal fasciculus (l) FA | -0.002 (-0.010, 0.006) | 0.583 | 0.729 |
| NfL | inferior fronto-occipital fasciculus (r) FA | 0.001 (-0.007, 0.008) | 0.895 | 0.931 |
| NfL | inferior fronto-occipital fasciculus (l) FA | 0.004 (-0.004, 0.013) | 0.339 | 0.538 |
| NfL | forceps major (r) FA | 0.002 (-0.005, 0.010) | 0.531 | 0.681 |
| NfL | forceps major (l) FA | -0.003 (-0.012, 0.005) | 0.462 | 0.630 |
| NfL | corticospinal tract (r) FA | 0.013 (0.002, 0.023) | 0.017 | 0.085 |
| NfL | corticospinal tract (l) FA | 0.000 (-0.011, 0.011) | 0.986 | 0.986 |
| NfL | cingulate gyrus part of cingulum (r) FA | -0.002 (-0.012, 0.007) | 0.611 | 0.743 |
| NfL | cingulate gyrus part of cingulum (l) FA | 0.003 (-0.007, 0.014) | 0.512 | 0.665 |
| NfL | anterior thalamic radiation (r) FA | 0.006 (-0.002, 0.015) | 0.154 | 0.320 |
| NfL | anterior thalamic radiation (l) FA | 0.007 (-0.003, 0.016) | 0.160 | 0.324 |
| NfL | acoustic radiation (r) FA | 0.004 (-0.008, 0.015) | 0.556 | 0.701 |
| NfL | acoustic radiation (l) FA | 0.005 (-0.007, 0.018) | 0.398 | 0.604 |
| p-tau181 | uncinate fasciculus (r) FA | 0.001 (-0.011, 0.013) | 0.860 | 0.926 |
| p-tau181 | uncinate fasciculus (l) FA | -0.005 (-0.017, 0.007) | 0.424 | 0.615 |
| p-tau181 | superior thalamic radiation (r) FA | 0.002 (-0.005, 0.010) | 0.533 | 0.713 |
| p-tau181 | superior thalamic radiation (l) FA | 0.000 (-0.007, 0.008) | 0.898 | 0.940 |
| p-tau181 | superior longitudinal fasciculus (r) FA | -0.001 (-0.007, 0.006) | 0.877 | 0.932 |
| p-tau181 | superior longitudinal fasciculus (l) FA | -0.006 (-0.013, 0.001) | 0.115 | 0.311 |
| p-tau181 | posterior thalamic radiation (r) FA | 0.004 (-0.006, 0.014) | 0.486 | 0.669 |
| p-tau181 | posterior thalamic radiation (l) FA | 0.000 (-0.010, 0.010) | 0.946 | 0.975 |
| p-tau181 | parahippocampal part of cingulum (r) FA | 0.013 (-0.001, 0.026) | 0.063 | 0.262 |
| p-tau181 | parahippocampal part of cingulum (l) FA | 0.007 (-0.008, 0.021) | 0.356 | 0.579 |
| p-tau181 | middle cerebellar peduncle (l) FA | -0.010 (-0.023, 0.003) | 0.136 | 0.311 |
| p-tau181 | medial lemniscus (r) FA | -0.006 (-0.020, 0.008) | 0.403 | 0.615 |
| p-tau181 | medial lemniscus (l) FA | -0.007 (-0.021, 0.007) | 0.306 | 0.530 |
| p-tau181 | inferior longitudinal fasciculus (r) FA | 0.001 (-0.007, 0.009) | 0.731 | 0.851 |
| p-tau181 | inferior longitudinal fasciculus (l) FA | 0.004 (-0.004, 0.012) | 0.312 | 0.530 |
| p-tau181 | inferior fronto-occipital fasciculus (r) FA | 0.005 (-0.003, 0.012) | 0.255 | 0.467 |
| p-tau181 | inferior fronto-occipital fasciculus (l) FA | 0.005 (-0.004, 0.014) | 0.256 | 0.467 |
| p-tau181 | forceps major (r) FA | 0.003 (-0.004, 0.010) | 0.417 | 0.615 |
| p-tau181 | forceps major (l) FA | -0.003 (-0.011, 0.006) | 0.551 | 0.722 |
| p-tau181 | corticospinal tract (r) FA | 0.002 (-0.008, 0.012) | 0.714 | 0.838 |
| p-tau181 | corticospinal tract (l) FA | -0.004 (-0.015, 0.006) | 0.424 | 0.615 |
| p-tau181 | cingulate gyrus part of cingulum (r) FA | 0.008 (-0.002, 0.017) | 0.116 | 0.311 |
| p-tau181 | cingulate gyrus part of cingulum (l) FA | 0.000 (-0.011, 0.010) | 0.930 | 0.966 |
| p-tau181 | anterior thalamic radiation (r) FA | -0.001 (-0.010, 0.008) | 0.824 | 0.909 |
| p-tau181 | anterior thalamic radiation (l) FA | 0.007 (-0.002, 0.016) | 0.132 | 0.311 |
| p-tau181 | acoustic radiation (r) FA | 0.008 (-0.004, 0.019) | 0.198 | 0.386 |
| p-tau181 | acoustic radiation (l) FA | -0.003 (-0.015, 0.009) | 0.601 | 0.765 |
| Aβ42/40 | uncinate fasciculus (r) MD | -0.010 (-0.024, 0.004) | 0.153 | 0.991 |
| Aβ42/40 | uncinate fasciculus (l) MD | -0.006 (-0.021, 0.008) | 0.398 | 0.991 |
| Aβ42/40 | superior thalamic radiation (r) MD | -0.002 (-0.015, 0.011) | 0.779 | 0.991 |
| Aβ42/40 | superior thalamic radiation (l) MD | -0.004 (-0.016, 0.008) | 0.469 | 0.991 |
| Aβ42/40 | superior longitudinal fasciculus (r) MD | -0.003 (-0.013, 0.006) | 0.479 | 0.991 |
| Aβ42/40 | superior longitudinal fasciculus (l) MD | -0.003 (-0.012, 0.006) | 0.479 | 0.991 |
| Aβ42/40 | posterior thalamic radiation (r) MD | -0.006 (-0.017, 0.005) | 0.323 | 0.991 |
| Aβ42/40 | posterior thalamic radiation (l) MD | -0.004 (-0.016, 0.007) | 0.454 | 0.991 |
| Aβ42/40 | parahippocampal part of cingulum (r) MD | -0.002 (-0.017, 0.013) | 0.782 | 0.991 |
| Aβ42/40 | parahippocampal part of cingulum (l) MD | 0.002 (-0.014, 0.017) | 0.841 | 0.991 |
| Aβ42/40 | middle cerebellar peduncle (l) MD | 0.004 (-0.012, 0.020) | 0.648 | 0.991 |
| Aβ42/40 | medial lemniscus (r) MD | 0.003 (-0.016, 0.022) | 0.725 | 0.991 |
| Aβ42/40 | medial lemniscus (l) MD | 0.014 (-0.005, 0.033) | 0.160 | 0.991 |
| Aβ42/40 | inferior longitudinal fasciculus (r) MD | -0.005 (-0.016, 0.007) | 0.450 | 0.991 |
| Aβ42/40 | inferior longitudinal fasciculus (l) MD | -0.001 (-0.012, 0.011) | 0.890 | 0.991 |
| Aβ42/40 | inferior fronto-occipital fasciculus (r) MD | -0.002 (-0.013, 0.009) | 0.733 | 0.991 |
| Aβ42/40 | inferior fronto-occipital fasciculus (l) MD | -0.009 (-0.019, 0.002) | 0.114 | 0.991 |
| Aβ42/40 | forceps major (r) MD | -0.012 (-0.023, -0.001) | 0.026 | 0.878 |
| Aβ42/40 | forceps major (l) MD | 0.001 (-0.010, 0.011) | 0.893 | 0.991 |
| Aβ42/40 | corticospinal tract (r) MD | -0.006 (-0.022, 0.009) | 0.440 | 0.991 |
| Aβ42/40 | corticospinal tract (l) MD | 0.000 (-0.016, 0.015) | 0.965 | 0.991 |
| Aβ42/40 | cingulate gyrus part of cingulum (r) MD | -0.003 (-0.017, 0.010) | 0.609 | 0.991 |
| Aβ42/40 | cingulate gyrus part of cingulum (l) MD | 0.005 (-0.009, 0.018) | 0.488 | 0.991 |
| Aβ42/40 | anterior thalamic radiation (r) MD | 0.001 (-0.009, 0.012) | 0.781 | 0.991 |
| Aβ42/40 | anterior thalamic radiation (l) MD | -0.003 (-0.014, 0.009) | 0.658 | 0.991 |
| Aβ42/40 | acoustic radiation (r) MD | 0.002 (-0.013, 0.018) | 0.768 | 0.991 |
| Aβ42/40 | acoustic radiation (l) MD | 0.009 (-0.006, 0.025) | 0.250 | 0.991 |
| GFAP | uncinate fasciculus (r) MD | 0.023 (0.010, 0.036) | 0.001 | 0.034 |
| GFAP | uncinate fasciculus (l) MD | 0.026 (0.012, 0.039) | 0.000 | 0.000 |
| GFAP | superior thalamic radiation (r) MD | -0.001 (-0.013, 0.012) | 0.892 | 0.927 |
| GFAP | superior thalamic radiation (l) MD | 0.001 (-0.010, 0.012) | 0.889 | 0.927 |
| GFAP | superior longitudinal fasciculus (r) MD | 0.001 (-0.008, 0.010) | 0.851 | 0.917 |
| GFAP | superior longitudinal fasciculus (l) MD | 0.009 (0.001, 0.018) | 0.032 | 0.187 |
| GFAP | posterior thalamic radiation (r) MD | 0.001 (-0.009, 0.011) | 0.842 | 0.917 |
| GFAP | posterior thalamic radiation (l) MD | 0.016 (0.005, 0.027) | 0.004 | 0.068 |
| GFAP | parahippocampal part of cingulum (r) MD | 0.007 (-0.007, 0.021) | 0.353 | 0.565 |
| GFAP | parahippocampal part of cingulum (l) MD | 0.006 (-0.008, 0.021) | 0.410 | 0.615 |
| GFAP | middle cerebellar peduncle (l) MD | 0.008 (-0.007, 0.023) | 0.287 | 0.531 |
| GFAP | medial lemniscus (r) MD | 0.001 (-0.017, 0.019) | 0.912 | 0.940 |
| GFAP | medial lemniscus (l) MD | 0.020 (0.002, 0.038) | 0.034 | 0.187 |
| GFAP | inferior longitudinal fasciculus (r) MD | 0.011 (0.000, 0.022) | 0.046 | 0.214 |
| GFAP | inferior longitudinal fasciculus (l) MD | 0.019 (0.008, 0.030) | 0.001 | 0.034 |
| GFAP | inferior fronto-occipital fasciculus (r) MD | 0.010 (0.000, 0.020) | 0.055 | 0.218 |
| GFAP | inferior fronto-occipital fasciculus (l) MD | 0.014 (0.004, 0.024) | 0.007 | 0.079 |
| GFAP | forceps major (r) MD | 0.008 (-0.002, 0.019) | 0.098 | 0.276 |
| GFAP | forceps major (l) MD | 0.015 (0.005, 0.025) | 0.003 | 0.068 |
| GFAP | corticospinal tract (r) MD | -0.001 (-0.016, 0.014) | 0.893 | 0.927 |
| GFAP | corticospinal tract (l) MD | 0.008 (-0.007, 0.023) | 0.280 | 0.531 |
| GFAP | cingulate gyrus part of cingulum (r) MD | -0.013 (-0.025, -0.001) | 0.039 | 0.195 |
| GFAP | cingulate gyrus part of cingulum (l) MD | -0.014 (-0.027, -0.001) | 0.033 | 0.187 |
| GFAP | anterior thalamic radiation (r) MD | -0.006 (-0.015, 0.004) | 0.264 | 0.531 |
| GFAP | anterior thalamic radiation (l) MD | 0.008 (-0.003, 0.018) | 0.152 | 0.387 |
| GFAP | acoustic radiation (r) MD | -0.007 (-0.022, 0.008) | 0.360 | 0.565 |
| GFAP | acoustic radiation (l) MD | 0.013 (-0.001, 0.028) | 0.080 | 0.257 |
| NfL | uncinate fasciculus (r) MD | 0.016 (0.003, 0.028) | 0.014 | 0.081 |
| NfL | uncinate fasciculus (l) MD | 0.018 (0.005, 0.031) | 0.007 | 0.053 |
| NfL | superior thalamic radiation (r) MD | 0.008 (-0.004, 0.020) | 0.175 | 0.347 |
| NfL | superior thalamic radiation (l) MD | 0.006 (-0.005, 0.017) | 0.274 | 0.474 |
| NfL | superior longitudinal fasciculus (r) MD | 0.007 (-0.002, 0.015) | 0.130 | 0.297 |
| NfL | superior longitudinal fasciculus (l) MD | 0.009 (0.001, 0.018) | 0.025 | 0.099 |
| NfL | posterior thalamic radiation (r) MD | 0.010 (0.000, 0.020) | 0.045 | 0.138 |
| NfL | posterior thalamic radiation (l) MD | 0.018 (0.008, 0.029) | 0.001 | 0.023 |
| NfL | parahippocampal part of cingulum (r) MD | 0.005 (-0.008, 0.019) | 0.456 | 0.630 |
| NfL | parahippocampal part of cingulum (l) MD | 0.006 (-0.008, 0.020) | 0.376 | 0.577 |
| NfL | middle cerebellar peduncle (l) MD | 0.005 (-0.009, 0.020) | 0.482 | 0.638 |
| NfL | medial lemniscus (r) MD | 0.020 (0.003, 0.037) | 0.024 | 0.098 |
| NfL | medial lemniscus (l) MD | 0.026 (0.008, 0.043) | 0.004 | 0.045 |
| NfL | inferior longitudinal fasciculus (r) MD | 0.015 (0.004, 0.026) | 0.006 | 0.053 |
| NfL | inferior longitudinal fasciculus (l) MD | 0.019 (0.009, 0.030) | 0.000 | 0.000 |
| NfL | inferior fronto-occipital fasciculus (r) MD | 0.011 (0.001, 0.021) | 0.034 | 0.121 |
| NfL | inferior fronto-occipital fasciculus (l) MD | 0.010 (0.000, 0.020) | 0.040 | 0.135 |
| NfL | forceps major (r) MD | 0.007 (-0.002, 0.017) | 0.142 | 0.309 |
| NfL | forceps major (l) MD | 0.007 (-0.002, 0.017) | 0.134 | 0.297 |
| NfL | corticospinal tract (r) MD | 0.015 (0.001, 0.029) | 0.033 | 0.120 |
| NfL | corticospinal tract (l) MD | 0.010 (-0.004, 0.024) | 0.150 | 0.316 |
| NfL | cingulate gyrus part of cingulum (r) MD | -0.007 (-0.019, 0.005) | 0.253 | 0.462 |
| NfL | cingulate gyrus part of cingulum (l) MD | -0.012 (-0.024, 0.000) | 0.057 | 0.160 |
| NfL | anterior thalamic radiation (r) MD | -0.004 (-0.013, 0.006) | 0.454 | 0.630 |
| NfL | anterior thalamic radiation (l) MD | 0.007 (-0.003, 0.017) | 0.161 | 0.324 |
| NfL | acoustic radiation (r) MD | 0.003 (-0.011, 0.017) | 0.699 | 0.786 |
| NfL | acoustic radiation (l) MD | 0.020 (0.006, 0.034) | 0.005 | 0.052 |
| p-tau181 | uncinate fasciculus (r) MD | 0.011 (-0.001, 0.024) | 0.074 | 0.270 |
| p-tau181 | uncinate fasciculus (l) MD | 0.010 (-0.003, 0.024) | 0.123 | 0.311 |
| p-tau181 | superior thalamic radiation (r) MD | 0.003 (-0.009, 0.015) | 0.660 | 0.809 |
| p-tau181 | superior thalamic radiation (l) MD | 0.009 (-0.002, 0.020) | 0.112 | 0.311 |
| p-tau181 | superior longitudinal fasciculus (r) MD | 0.007 (-0.002, 0.015) | 0.140 | 0.315 |
| p-tau181 | superior longitudinal fasciculus (l) MD | 0.011 (0.003, 0.020) | 0.007 | 0.152 |
| p-tau181 | posterior thalamic radiation (r) MD | 0.009 (-0.001, 0.019) | 0.065 | 0.262 |
| p-tau181 | posterior thalamic radiation (l) MD | 0.016 (0.006, 0.027) | 0.003 | 0.101 |
| p-tau181 | parahippocampal part of cingulum (r) MD | 0.003 (-0.011, 0.016) | 0.688 | 0.822 |
| p-tau181 | parahippocampal part of cingulum (l) MD | 0.014 (0.000, 0.028) | 0.056 | 0.262 |
| p-tau181 | middle cerebellar peduncle (l) MD | 0.015 (0.000, 0.029) | 0.049 | 0.260 |
| p-tau181 | medial lemniscus (r) MD | 0.016 (-0.001, 0.034) | 0.066 | 0.262 |
| p-tau181 | medial lemniscus (l) MD | 0.015 (-0.002, 0.033) | 0.092 | 0.305 |
| p-tau181 | inferior longitudinal fasciculus (r) MD | 0.013 (0.002, 0.024) | 0.017 | 0.153 |
| p-tau181 | inferior longitudinal fasciculus (l) MD | 0.014 (0.003, 0.024) | 0.011 | 0.153 |
| p-tau181 | inferior fronto-occipital fasciculus (r) MD | 0.009 (-0.001, 0.019) | 0.085 | 0.302 |
| p-tau181 | inferior fronto-occipital fasciculus (l) MD | 0.010 (0.000, 0.020) | 0.043 | 0.252 |
| p-tau181 | forceps major (r) MD | 0.008 (-0.002, 0.018) | 0.101 | 0.305 |
| p-tau181 | forceps major (l) MD | 0.003 (-0.007, 0.013) | 0.538 | 0.713 |
| p-tau181 | corticospinal tract (r) MD | 0.015 (0.001, 0.030) | 0.032 | 0.227 |
| p-tau181 | corticospinal tract (l) MD | 0.011 (-0.003, 0.026) | 0.113 | 0.311 |
| p-tau181 | cingulate gyrus part of cingulum (r) MD | -0.007 (-0.019, 0.005) | 0.226 | 0.430 |
| p-tau181 | cingulate gyrus part of cingulum (l) MD | -0.009 (-0.021, 0.004) | 0.177 | 0.351 |
| p-tau181 | anterior thalamic radiation (r) MD | 0.001 (-0.008, 0.011) | 0.828 | 0.909 |
| p-tau181 | anterior thalamic radiation (l) MD | 0.008 (-0.002, 0.018) | 0.125 | 0.311 |
| p-tau181 | acoustic radiation (r) MD | 0.003 (-0.011, 0.018) | 0.649 | 0.804 |
| p-tau181 | acoustic radiation (l) MD | 0.015 (0.001, 0.029) | 0.037 | 0.250 |
| Aβ42/40 | uncinate fasciculus (r) ICVF | 0.002 (-0.013, 0.017) | 0.770 | 0.991 |
| Aβ42/40 | uncinate fasciculus (l) ICVF | -0.001 (-0.016, 0.013) | 0.882 | 0.991 |
| Aβ42/40 | superior thalamic radiation (r) ICVF | -0.001 (-0.013, 0.012) | 0.916 | 0.991 |
| Aβ42/40 | superior thalamic radiation (l) ICVF | 0.004 (-0.008, 0.016) | 0.516 | 0.991 |
| Aβ42/40 | superior longitudinal fasciculus (r) ICVF | 0.003 (-0.007, 0.012) | 0.580 | 0.991 |
| Aβ42/40 | superior longitudinal fasciculus (l) ICVF | 0.004 (-0.005, 0.013) | 0.408 | 0.991 |
| Aβ42/40 | posterior thalamic radiation (r) ICVF | -0.001 (-0.013, 0.011) | 0.867 | 0.991 |
| Aβ42/40 | posterior thalamic radiation (l) ICVF | -0.001 (-0.013, 0.011) | 0.877 | 0.991 |
| Aβ42/40 | parahippocampal part of cingulum (r) ICVF | -0.005 (-0.022, 0.011) | 0.544 | 0.991 |
| Aβ42/40 | parahippocampal part of cingulum (l) ICVF | -0.006 (-0.023, 0.010) | 0.477 | 0.991 |
| Aβ42/40 | middle cerebellar peduncle (l) ICVF | 0.012 (-0.002, 0.026) | 0.107 | 0.991 |
| Aβ42/40 | medial lemniscus (r) ICVF | 0.005 (-0.013, 0.022) | 0.595 | 0.991 |
| Aβ42/40 | medial lemniscus (l) ICVF | 0.000 (-0.017, 0.018) | 0.982 | 0.991 |
| Aβ42/40 | inferior longitudinal fasciculus (r) ICVF | 0.000 (-0.013, 0.012) | 0.937 | 0.991 |
| Aβ42/40 | inferior longitudinal fasciculus (l) ICVF | 0.001 (-0.011, 0.013) | 0.907 | 0.991 |
| Aβ42/40 | inferior fronto-occipital fasciculus (r) ICVF | 0.001 (-0.010, 0.012) | 0.869 | 0.991 |
| Aβ42/40 | inferior fronto-occipital fasciculus (l) ICVF | 0.002 (-0.009, 0.014) | 0.698 | 0.991 |
| Aβ42/40 | forceps major (r) ICVF | -0.002 (-0.012, 0.009) | 0.727 | 0.991 |
| Aβ42/40 | forceps major (l) ICVF | 0.000 (-0.012, 0.012) | 0.993 | 0.993 |
| Aβ42/40 | corticospinal tract (r) ICVF | -0.002 (-0.016, 0.011) | 0.713 | 0.991 |
| Aβ42/40 | corticospinal tract (l) ICVF | 0.003 (-0.010, 0.016) | 0.634 | 0.991 |
| Aβ42/40 | cingulate gyrus part of cingulum (r) ICVF | -0.001 (-0.014, 0.012) | 0.886 | 0.991 |
| Aβ42/40 | cingulate gyrus part of cingulum (l) ICVF | 0.000 (-0.013, 0.012) | 0.941 | 0.991 |
| Aβ42/40 | anterior thalamic radiation (r) ICVF | -0.002 (-0.015, 0.010) | 0.701 | 0.991 |
| Aβ42/40 | anterior thalamic radiation (l) ICVF | 0.000 (-0.012, 0.012) | 0.968 | 0.991 |
| Aβ42/40 | acoustic radiation (r) ICVF | -0.003 (-0.017, 0.010) | 0.622 | 0.991 |
| Aβ42/40 | acoustic radiation (l) ICVF | -0.004 (-0.017, 0.010) | 0.599 | 0.991 |
| GFAP | uncinate fasciculus (r) ICVF | -0.015 (-0.029, -0.001) | 0.033 | 0.187 |
| GFAP | uncinate fasciculus (l) ICVF | -0.020 (-0.034, -0.007) | 0.004 | 0.068 |
| GFAP | superior thalamic radiation (r) ICVF | 0.004 (-0.008, 0.016) | 0.510 | 0.689 |
| GFAP | superior thalamic radiation (l) ICVF | -0.002 (-0.013, 0.010) | 0.779 | 0.891 |
| GFAP | superior longitudinal fasciculus (r) ICVF | -0.003 (-0.012, 0.007) | 0.587 | 0.762 |
| GFAP | superior longitudinal fasciculus (l) ICVF | -0.009 (-0.017, 0.000) | 0.053 | 0.217 |
| GFAP | posterior thalamic radiation (r) ICVF | 0.000 (-0.012, 0.012) | 0.984 | 0.984 |
| GFAP | posterior thalamic radiation (l) ICVF | -0.010 (-0.021, 0.002) | 0.094 | 0.276 |
| GFAP | parahippocampal part of cingulum (r) ICVF | -0.009 (-0.025, 0.006) | 0.247 | 0.529 |
| GFAP | parahippocampal part of cingulum (l) ICVF | -0.012 (-0.027, 0.004) | 0.146 | 0.379 |
| GFAP | middle cerebellar peduncle (l) ICVF | -0.005 (-0.019, 0.008) | 0.439 | 0.630 |
| GFAP | medial lemniscus (r) ICVF | -0.015 (-0.032, 0.001) | 0.069 | 0.245 |
| GFAP | medial lemniscus (l) ICVF | -0.010 (-0.027, 0.007) | 0.259 | 0.531 |
| GFAP | inferior longitudinal fasciculus (r) ICVF | -0.011 (-0.022, 0.001) | 0.075 | 0.253 |
| GFAP | inferior longitudinal fasciculus (l) ICVF | -0.014 (-0.025, -0.003) | 0.012 | 0.108 |
| GFAP | inferior fronto-occipital fasciculus (r) ICVF | -0.008 (-0.019, 0.002) | 0.118 | 0.325 |
| GFAP | inferior fronto-occipital fasciculus (l) ICVF | -0.010 (-0.020, 0.001) | 0.080 | 0.257 |
| GFAP | forceps major (r) ICVF | -0.002 (-0.011, 0.008) | 0.759 | 0.891 |
| GFAP | forceps major (l) ICVF | -0.014 (-0.026, -0.002) | 0.018 | 0.152 |
| GFAP | corticospinal tract (r) ICVF | 0.009 (-0.004, 0.021) | 0.165 | 0.398 |
| GFAP | corticospinal tract (l) ICVF | 0.002 (-0.010, 0.015) | 0.713 | 0.891 |
| GFAP | cingulate gyrus part of cingulum (r) ICVF | 0.002 (-0.010, 0.015) | 0.694 | 0.876 |
| GFAP | cingulate gyrus part of cingulum (l) ICVF | -0.003 (-0.015, 0.009) | 0.646 | 0.831 |
| GFAP | anterior thalamic radiation (r) ICVF | 0.002 (-0.010, 0.013) | 0.759 | 0.891 |
| GFAP | anterior thalamic radiation (l) ICVF | -0.006 (-0.017, 0.006) | 0.337 | 0.565 |
| GFAP | acoustic radiation (r) ICVF | 0.000 (-0.013, 0.013) | 0.975 | 0.982 |
| GFAP | acoustic radiation (l) ICVF | -0.004 (-0.016, 0.009) | 0.566 | 0.742 |
| NfL | uncinate fasciculus (r) ICVF | -0.016 (-0.030, -0.003) | 0.018 | 0.087 |
| NfL | uncinate fasciculus (l) ICVF | -0.017 (-0.030, -0.004) | 0.013 | 0.080 |
| NfL | superior thalamic radiation (r) ICVF | -0.006 (-0.017, 0.006) | 0.317 | 0.509 |
| NfL | superior thalamic radiation (l) ICVF | -0.006 (-0.017, 0.005) | 0.258 | 0.463 |
| NfL | superior longitudinal fasciculus (r) ICVF | -0.008 (-0.017, 0.000) | 0.059 | 0.163 |
| NfL | superior longitudinal fasciculus (l) ICVF | -0.011 (-0.020, -0.003) | 0.008 | 0.057 |
| NfL | posterior thalamic radiation (r) ICVF | -0.009 (-0.020, 0.002) | 0.127 | 0.297 |
| NfL | posterior thalamic radiation (l) ICVF | -0.014 (-0.025, -0.002) | 0.017 | 0.085 |
| NfL | parahippocampal part of cingulum (r) ICVF | -0.018 (-0.033, -0.004) | 0.015 | 0.081 |
| NfL | parahippocampal part of cingulum (l) ICVF | -0.016 (-0.031, -0.001) | 0.040 | 0.135 |
| NfL | middle cerebellar peduncle (l) ICVF | -0.020 (-0.033, -0.007) | 0.002 | 0.027 |
| NfL | medial lemniscus (r) ICVF | -0.020 (-0.036, -0.004) | 0.015 | 0.081 |
| NfL | medial lemniscus (l) ICVF | -0.009 (-0.025, 0.008) | 0.301 | 0.496 |
| NfL | inferior longitudinal fasciculus (r) ICVF | -0.015 (-0.026, -0.004) | 0.009 | 0.061 |
| NfL | inferior longitudinal fasciculus (l) ICVF | -0.016 (-0.026, -0.005) | 0.004 | 0.045 |
| NfL | inferior fronto-occipital fasciculus (r) ICVF | -0.011 (-0.021, -0.001) | 0.031 | 0.116 |
| NfL | inferior fronto-occipital fasciculus (l) ICVF | -0.012 (-0.022, -0.002) | 0.024 | 0.098 |
| NfL | forceps major (r) ICVF | -0.002 (-0.012, 0.007) | 0.609 | 0.743 |
| NfL | forceps major (l) ICVF | -0.018 (-0.029, -0.007) | 0.002 | 0.027 |
| NfL | corticospinal tract (r) ICVF | -0.007 (-0.019, 0.005) | 0.286 | 0.478 |
| NfL | corticospinal tract (l) ICVF | -0.008 (-0.020, 0.004) | 0.199 | 0.384 |
| NfL | cingulate gyrus part of cingulum (r) ICVF | -0.003 (-0.014, 0.009) | 0.664 | 0.784 |
| NfL | cingulate gyrus part of cingulum (l) ICVF | -0.006 (-0.017, 0.006) | 0.351 | 0.551 |
| NfL | anterior thalamic radiation (r) ICVF | -0.007 (-0.018, 0.004) | 0.232 | 0.435 |
| NfL | anterior thalamic radiation (l) ICVF | -0.008 (-0.020, 0.003) | 0.133 | 0.297 |
| NfL | acoustic radiation (r) ICVF | -0.011 (-0.023, 0.002) | 0.096 | 0.240 |
| NfL | acoustic radiation (l) ICVF | -0.008 (-0.021, 0.004) | 0.178 | 0.348 |
| p-tau181 | uncinate fasciculus (r) ICVF | -0.014 (-0.028, -0.001) | 0.043 | 0.252 |
| p-tau181 | uncinate fasciculus (l) ICVF | -0.021 (-0.034, -0.007) | 0.002 | 0.101 |
| p-tau181 | superior thalamic radiation (r) ICVF | 0.002 (-0.009, 0.013) | 0.747 | 0.855 |
| p-tau181 | superior thalamic radiation (l) ICVF | 0.000 (-0.011, 0.011) | 0.988 | 0.988 |
| p-tau181 | superior longitudinal fasciculus (r) ICVF | -0.005 (-0.014, 0.004) | 0.284 | 0.498 |
| p-tau181 | superior longitudinal fasciculus (l) ICVF | -0.006 (-0.015, 0.002) | 0.145 | 0.321 |
| p-tau181 | posterior thalamic radiation (r) ICVF | -0.002 (-0.013, 0.009) | 0.704 | 0.834 |
| p-tau181 | posterior thalamic radiation (l) ICVF | -0.007 (-0.019, 0.004) | 0.200 | 0.386 |
| p-tau181 | parahippocampal part of cingulum (r) ICVF | -0.010 (-0.025, 0.005) | 0.176 | 0.351 |
| p-tau181 | parahippocampal part of cingulum (l) ICVF | -0.015 (-0.030, 0.000) | 0.046 | 0.259 |
| p-tau181 | middle cerebellar peduncle (l) ICVF | -0.012 (-0.025, 0.001) | 0.070 | 0.270 |
| p-tau181 | medial lemniscus (r) ICVF | -0.014 (-0.030, 0.002) | 0.096 | 0.305 |
| p-tau181 | medial lemniscus (l) ICVF | -0.015 (-0.031, 0.001) | 0.072 | 0.270 |
| p-tau181 | inferior longitudinal fasciculus (r) ICVF | -0.007 (-0.018, 0.005) | 0.254 | 0.467 |
| p-tau181 | inferior longitudinal fasciculus (l) ICVF | -0.008 (-0.019, 0.003) | 0.158 | 0.333 |
| p-tau181 | inferior fronto-occipital fasciculus (r) ICVF | -0.007 (-0.017, 0.003) | 0.173 | 0.351 |
| p-tau181 | inferior fronto-occipital fasciculus (l) ICVF | -0.008 (-0.018, 0.002) | 0.132 | 0.311 |
| p-tau181 | forceps major (r) ICVF | -0.003 (-0.013, 0.006) | 0.504 | 0.687 |
| p-tau181 | forceps major (l) ICVF | -0.005 (-0.016, 0.006) | 0.407 | 0.615 |
| p-tau181 | corticospinal tract (r) ICVF | -0.002 (-0.014, 0.010) | 0.745 | 0.855 |
| p-tau181 | corticospinal tract (l) ICVF | -0.003 (-0.015, 0.009) | 0.666 | 0.809 |
| p-tau181 | cingulate gyrus part of cingulum (r) ICVF | 0.000 (-0.012, 0.012) | 0.988 | 0.988 |
| p-tau181 | cingulate gyrus part of cingulum (l) ICVF | -0.006 (-0.017, 0.006) | 0.346 | 0.576 |
| p-tau181 | anterior thalamic radiation (r) ICVF | -0.011 (-0.022, 0.000) | 0.050 | 0.260 |
| p-tau181 | anterior thalamic radiation (l) ICVF | -0.011 (-0.022, 0.001) | 0.063 | 0.262 |
| p-tau181 | acoustic radiation (r) ICVF | -0.010 (-0.023, 0.002) | 0.104 | 0.305 |
| p-tau181 | acoustic radiation (l) ICVF | -0.006 (-0.019, 0.006) | 0.314 | 0.530 |
| Aβ42/40 | uncinate fasciculus (r) ISOVF | 0.004 (-0.011, 0.018) | 0.634 | 0.991 |
| Aβ42/40 | uncinate fasciculus (l) ISOVF | 0.004 (-0.011, 0.018) | 0.634 | 0.991 |
| Aβ42/40 | superior thalamic radiation (r) ISOVF | 0.004 (-0.011, 0.018) | 0.634 | 0.991 |
| Aβ42/40 | superior thalamic radiation (l) ISOVF | 0.004 (-0.011, 0.018) | 0.634 | 0.991 |
| Aβ42/40 | superior longitudinal fasciculus (r) ISOVF | 0.001 (-0.012, 0.013) | 0.909 | 0.991 |
| Aβ42/40 | superior longitudinal fasciculus (l) ISOVF | 0.005 (-0.007, 0.018) | 0.398 | 0.991 |
| Aβ42/40 | posterior thalamic radiation (r) ISOVF | -0.011 (-0.025, 0.003) | 0.116 | 0.991 |
| Aβ42/40 | posterior thalamic radiation (l) ISOVF | -0.004 (-0.018, 0.010) | 0.609 | 0.991 |
| Aβ42/40 | parahippocampal part of cingulum (r) ISOVF | -0.006 (-0.022, 0.009) | 0.408 | 0.991 |
| Aβ42/40 | parahippocampal part of cingulum (l) ISOVF | -0.004 (-0.021, 0.012) | 0.617 | 0.991 |
| Aβ42/40 | middle cerebellar peduncle (l) ISOVF | 0.006 (-0.011, 0.022) | 0.488 | 0.991 |
| Aβ42/40 | medial lemniscus (r) ISOVF | 0.005 (-0.013, 0.024) | 0.574 | 0.991 |
| Aβ42/40 | medial lemniscus (l) ISOVF | 0.014 (-0.005, 0.033) | 0.160 | 0.991 |
| Aβ42/40 | inferior longitudinal fasciculus (r) ISOVF | -0.006 (-0.020, 0.008) | 0.392 | 0.991 |
| Aβ42/40 | inferior longitudinal fasciculus (l) ISOVF | 0.004 (-0.011, 0.019) | 0.605 | 0.991 |
| Aβ42/40 | inferior fronto-occipital fasciculus (r) ISOVF | -0.001 (-0.015, 0.013) | 0.901 | 0.991 |
| Aβ42/40 | inferior fronto-occipital fasciculus (l) ISOVF | -0.004 (-0.018, 0.010) | 0.556 | 0.991 |
| Aβ42/40 | forceps major (r) ISOVF | -0.020 (-0.036, -0.004) | 0.012 | 0.675 |
| Aβ42/40 | forceps major (l) ISOVF | 0.002 (-0.010, 0.014) | 0.740 | 0.991 |
| Aβ42/40 | corticospinal tract (r) ISOVF | -0.003 (-0.021, 0.016) | 0.774 | 0.991 |
| Aβ42/40 | corticospinal tract (l) ISOVF | 0.005 (-0.014, 0.024) | 0.590 | 0.991 |
| Aβ42/40 | cingulate gyrus part of cingulum (r) ISOVF | -0.004 (-0.020, 0.013) | 0.660 | 0.991 |
| Aβ42/40 | cingulate gyrus part of cingulum (l) ISOVF | 0.008 (-0.009, 0.025) | 0.379 | 0.991 |
| Aβ42/40 | anterior thalamic radiation (r) ISOVF | -0.010 (-0.026, 0.006) | 0.242 | 0.991 |
| Aβ42/40 | anterior thalamic radiation (l) ISOVF | -0.004 (-0.020, 0.012) | 0.631 | 0.991 |
| Aβ42/40 | acoustic radiation (r) ISOVF | 0.005 (-0.013, 0.022) | 0.597 | 0.991 |
| Aβ42/40 | acoustic radiation (l) ISOVF | 0.011 (-0.006, 0.028) | 0.203 | 0.991 |
| GFAP | uncinate fasciculus (r) ISOVF | -0.007 (-0.020, 0.007) | 0.358 | 0.565 |
| GFAP | uncinate fasciculus (l) ISOVF | -0.007 (-0.020, 0.007) | 0.358 | 0.565 |
| GFAP | superior thalamic radiation (r) ISOVF | -0.007 (-0.020, 0.007) | 0.358 | 0.565 |
| GFAP | superior thalamic radiation (l) ISOVF | -0.007 (-0.020, 0.007) | 0.358 | 0.565 |
| GFAP | superior longitudinal fasciculus (r) ISOVF | 0.000 (-0.011, 0.012) | 0.955 | 0.977 |
| GFAP | superior longitudinal fasciculus (l) ISOVF | 0.004 (-0.008, 0.016) | 0.501 | 0.689 |
| GFAP | posterior thalamic radiation (r) ISOVF | 0.013 (0.000, 0.026) | 0.053 | 0.217 |
| GFAP | posterior thalamic radiation (l) ISOVF | 0.015 (0.002, 0.028) | 0.027 | 0.182 |
| GFAP | parahippocampal part of cingulum (r) ISOVF | 0.006 (-0.009, 0.020) | 0.434 | 0.630 |
| GFAP | parahippocampal part of cingulum (l) ISOVF | 0.003 (-0.013, 0.018) | 0.736 | 0.891 |
| GFAP | middle cerebellar peduncle (l) ISOVF | 0.007 (-0.008, 0.023) | 0.355 | 0.565 |
| GFAP | medial lemniscus (r) ISOVF | -0.003 (-0.020, 0.015) | 0.747 | 0.891 |
| GFAP | medial lemniscus (l) ISOVF | 0.018 (0.000, 0.036) | 0.057 | 0.218 |
| GFAP | inferior longitudinal fasciculus (r) ISOVF | 0.010 (-0.003, 0.023) | 0.145 | 0.379 |
| GFAP | inferior longitudinal fasciculus (l) ISOVF | 0.014 (0.000, 0.028) | 0.049 | 0.217 |
| GFAP | inferior fronto-occipital fasciculus (r) ISOVF | 0.007 (-0.007, 0.020) | 0.336 | 0.565 |
| GFAP | inferior fronto-occipital fasciculus (l) ISOVF | 0.011 (-0.002, 0.024) | 0.098 | 0.276 |
| GFAP | forceps major (r) ISOVF | 0.008 (-0.007, 0.023) | 0.272 | 0.531 |
| GFAP | forceps major (l) ISOVF | 0.011 (0.000, 0.022) | 0.045 | 0.214 |
| GFAP | corticospinal tract (r) ISOVF | 0.003 (-0.015, 0.020) | 0.768 | 0.891 |
| GFAP | corticospinal tract (l) ISOVF | 0.011 (-0.007, 0.029) | 0.238 | 0.518 |
| GFAP | cingulate gyrus part of cingulum (r) ISOVF | -0.008 (-0.024, 0.007) | 0.278 | 0.531 |
| GFAP | cingulate gyrus part of cingulum (l) ISOVF | -0.014 (-0.030, 0.002) | 0.087 | 0.267 |
| GFAP | anterior thalamic radiation (r) ISOVF | 0.008 (-0.007, 0.023) | 0.284 | 0.531 |
| GFAP | anterior thalamic radiation (l) ISOVF | 0.003 (-0.012, 0.018) | 0.685 | 0.872 |
| GFAP | acoustic radiation (r) ISOVF | -0.007 (-0.023, 0.010) | 0.422 | 0.626 |
| GFAP | acoustic radiation (l) ISOVF | 0.011 (-0.005, 0.027) | 0.179 | 0.424 |
| NfL | uncinate fasciculus (r) ISOVF | -0.003 (-0.016, 0.011) | 0.691 | 0.784 |
| NfL | uncinate fasciculus (l) ISOVF | -0.003 (-0.016, 0.011) | 0.691 | 0.784 |
| NfL | superior thalamic radiation (r) ISOVF | -0.003 (-0.016, 0.011) | 0.691 | 0.784 |
| NfL | superior thalamic radiation (l) ISOVF | -0.003 (-0.016, 0.011) | 0.691 | 0.784 |
| NfL | superior longitudinal fasciculus (r) ISOVF | 0.003 (-0.008, 0.014) | 0.627 | 0.756 |
| NfL | superior longitudinal fasciculus (l) ISOVF | 0.001 (-0.011, 0.012) | 0.905 | 0.931 |
| NfL | posterior thalamic radiation (r) ISOVF | 0.020 (0.007, 0.033) | 0.002 | 0.027 |
| NfL | posterior thalamic radiation (l) ISOVF | 0.015 (0.002, 0.028) | 0.020 | 0.093 |
| NfL | parahippocampal part of cingulum (r) ISOVF | 0.002 (-0.012, 0.017) | 0.739 | 0.812 |
| NfL | parahippocampal part of cingulum (l) ISOVF | 0.001 (-0.014, 0.016) | 0.904 | 0.931 |
| NfL | middle cerebellar peduncle (l) ISOVF | -0.001 (-0.016, 0.014) | 0.868 | 0.923 |
| NfL | medial lemniscus (r) ISOVF | 0.013 (-0.004, 0.030) | 0.133 | 0.297 |
| NfL | medial lemniscus (l) ISOVF | 0.024 (0.007, 0.042) | 0.007 | 0.053 |
| NfL | inferior longitudinal fasciculus (r) ISOVF | 0.011 (-0.002, 0.024) | 0.090 | 0.234 |
| NfL | inferior longitudinal fasciculus (l) ISOVF | 0.015 (0.002, 0.029) | 0.026 | 0.100 |
| NfL | inferior fronto-occipital fasciculus (r) ISOVF | 0.004 (-0.009, 0.017) | 0.535 | 0.681 |
| NfL | inferior fronto-occipital fasciculus (l) ISOVF | 0.001 (-0.012, 0.013) | 0.912 | 0.931 |
| NfL | forceps major (r) ISOVF | 0.006 (-0.008, 0.021) | 0.375 | 0.577 |
| NfL | forceps major (l) ISOVF | 0.001 (-0.010, 0.012) | 0.844 | 0.904 |
| NfL | corticospinal tract (r) ISOVF | 0.013 (-0.004, 0.030) | 0.124 | 0.297 |
| NfL | corticospinal tract (l) ISOVF | 0.009 (-0.008, 0.027) | 0.308 | 0.501 |
| NfL | cingulate gyrus part of cingulum (r) ISOVF | -0.006 (-0.021, 0.009) | 0.445 | 0.630 |
| NfL | cingulate gyrus part of cingulum (l) ISOVF | -0.020 (-0.036, -0.005) | 0.010 | 0.064 |
| NfL | anterior thalamic radiation (r) ISOVF | 0.006 (-0.009, 0.020) | 0.428 | 0.630 |
| NfL | anterior thalamic radiation (l) ISOVF | 0.001 (-0.014, 0.015) | 0.917 | 0.931 |
| NfL | acoustic radiation (r) ISOVF | -0.006 (-0.022, 0.009) | 0.424 | 0.630 |
| NfL | acoustic radiation (l) ISOVF | 0.016 (0.000, 0.031) | 0.049 | 0.144 |
| p-tau181 | uncinate fasciculus (r) ISOVF | 0.011 (-0.002, 0.025) | 0.102 | 0.305 |
| p-tau181 | uncinate fasciculus (l) ISOVF | 0.011 (-0.002, 0.025) | 0.102 | 0.305 |
| p-tau181 | superior thalamic radiation (r) ISOVF | 0.011 (-0.002, 0.025) | 0.102 | 0.305 |
| p-tau181 | superior thalamic radiation (l) ISOVF | 0.011 (-0.002, 0.025) | 0.102 | 0.305 |
| p-tau181 | superior longitudinal fasciculus (r) ISOVF | 0.005 (-0.007, 0.016) | 0.418 | 0.615 |
| p-tau181 | superior longitudinal fasciculus (l) ISOVF | 0.011 (0.000, 0.023) | 0.053 | 0.262 |
| p-tau181 | posterior thalamic radiation (r) ISOVF | 0.016 (0.003, 0.028) | 0.016 | 0.153 |
| p-tau181 | posterior thalamic radiation (l) ISOVF | 0.015 (0.002, 0.028) | 0.020 | 0.169 |
| p-tau181 | parahippocampal part of cingulum (r) ISOVF | 0.000 (-0.014, 0.014) | 0.985 | 0.988 |
| p-tau181 | parahippocampal part of cingulum (l) ISOVF | 0.009 (-0.006, 0.023) | 0.260 | 0.468 |
| p-tau181 | middle cerebellar peduncle (l) ISOVF | 0.012 (-0.003, 0.027) | 0.119 | 0.311 |
| p-tau181 | medial lemniscus (r) ISOVF | 0.013 (-0.004, 0.030) | 0.132 | 0.311 |
| p-tau181 | medial lemniscus (l) ISOVF | 0.014 (-0.004, 0.031) | 0.135 | 0.311 |
| p-tau181 | inferior longitudinal fasciculus (r) ISOVF | 0.016 (0.003, 0.029) | 0.013 | 0.153 |
| p-tau181 | inferior longitudinal fasciculus (l) ISOVF | 0.013 (0.000, 0.027) | 0.058 | 0.262 |
| p-tau181 | inferior fronto-occipital fasciculus (r) ISOVF | 0.005 (-0.008, 0.018) | 0.431 | 0.619 |
| p-tau181 | inferior fronto-occipital fasciculus (l) ISOVF | 0.003 (-0.009, 0.016) | 0.617 | 0.778 |
| p-tau181 | forceps major (r) ISOVF | 0.004 (-0.010, 0.019) | 0.539 | 0.713 |
| p-tau181 | forceps major (l) ISOVF | 0.001 (-0.010, 0.012) | 0.888 | 0.937 |
| p-tau181 | corticospinal tract (r) ISOVF | 0.016 (-0.001, 0.033) | 0.064 | 0.262 |
| p-tau181 | corticospinal tract (l) ISOVF | 0.014 (-0.004, 0.032) | 0.122 | 0.311 |
| p-tau181 | cingulate gyrus part of cingulum (r) ISOVF | -0.011 (-0.026, 0.004) | 0.155 | 0.333 |
| p-tau181 | cingulate gyrus part of cingulum (l) ISOVF | -0.016 (-0.032, -0.001) | 0.039 | 0.251 |
| p-tau181 | anterior thalamic radiation (r) ISOVF | -0.006 (-0.020, 0.008) | 0.416 | 0.615 |
| p-tau181 | anterior thalamic radiation (l) ISOVF | -0.003 (-0.018, 0.011) | 0.642 | 0.803 |
| p-tau181 | acoustic radiation (r) ISOVF | -0.007 (-0.023, 0.008) | 0.350 | 0.576 |
| p-tau181 | acoustic radiation (l) ISOVF | 0.007 (-0.009, 0.022) | 0.389 | 0.615 |
| Aβ42/40 | uncinate fasciculus (r) OD | 0.000 (-0.017, 0.016) | 0.978 | 0.991 |
| Aβ42/40 | uncinate fasciculus (l) OD | 0.006 (-0.010, 0.022) | 0.483 | 0.991 |
| Aβ42/40 | superior thalamic radiation (r) OD | 0.001 (-0.006, 0.007) | 0.868 | 0.991 |
| Aβ42/40 | superior thalamic radiation (l) OD | 0.003 (-0.004, 0.010) | 0.361 | 0.991 |
| Aβ42/40 | superior longitudinal fasciculus (r) OD | 0.005 (-0.004, 0.014) | 0.276 | 0.991 |
| Aβ42/40 | superior longitudinal fasciculus (l) OD | 0.003 (-0.006, 0.013) | 0.473 | 0.991 |
| Aβ42/40 | posterior thalamic radiation (r) OD | 0.006 (-0.011, 0.023) | 0.477 | 0.991 |
| Aβ42/40 | posterior thalamic radiation (l) OD | -0.003 (-0.020, 0.014) | 0.714 | 0.991 |
| Aβ42/40 | parahippocampal part of cingulum (r) OD | 0.004 (-0.013, 0.020) | 0.675 | 0.991 |
| Aβ42/40 | parahippocampal part of cingulum (l) OD | 0.012 (-0.005, 0.028) | 0.174 | 0.991 |
| Aβ42/40 | middle cerebellar peduncle (l) OD | 0.010 (-0.005, 0.025) | 0.172 | 0.991 |
| Aβ42/40 | medial lemniscus (r) OD | 0.001 (-0.011, 0.014) | 0.835 | 0.991 |
| Aβ42/40 | medial lemniscus (l) OD | 0.004 (-0.008, 0.016) | 0.532 | 0.991 |
| Aβ42/40 | inferior longitudinal fasciculus (r) OD | 0.000 (-0.015, 0.015) | 0.979 | 0.991 |
| Aβ42/40 | inferior longitudinal fasciculus (l) OD | 0.001 (-0.013, 0.016) | 0.855 | 0.991 |
| Aβ42/40 | inferior fronto-occipital fasciculus (r) OD | 0.019 (0.005, 0.033) | 0.007 | 0.675 |
| Aβ42/40 | inferior fronto-occipital fasciculus (l) OD | 0.013 (-0.002, 0.027) | 0.086 | 0.991 |
| Aβ42/40 | forceps major (r) OD | 0.000 (-0.011, 0.011) | 0.951 | 0.991 |
| Aβ42/40 | forceps major (l) OD | 0.007 (-0.005, 0.019) | 0.272 | 0.991 |
| Aβ42/40 | corticospinal tract (r) OD | 0.014 (0.003, 0.025) | 0.015 | 0.675 |
| Aβ42/40 | corticospinal tract (l) OD | 0.002 (-0.008, 0.011) | 0.736 | 0.991 |
| Aβ42/40 | cingulate gyrus part of cingulum (r) OD | -0.002 (-0.015, 0.011) | 0.765 | 0.991 |
| Aβ42/40 | cingulate gyrus part of cingulum (l) OD | 0.004 (-0.010, 0.018) | 0.553 | 0.991 |
| Aβ42/40 | anterior thalamic radiation (r) OD | 0.005 (-0.006, 0.016) | 0.384 | 0.991 |
| Aβ42/40 | anterior thalamic radiation (l) OD | 0.003 (-0.008, 0.014) | 0.644 | 0.991 |
| Aβ42/40 | acoustic radiation (r) OD | 0.000 (-0.013, 0.014) | 0.975 | 0.991 |
| Aβ42/40 | acoustic radiation (l) OD | -0.010 (-0.024, 0.004) | 0.177 | 0.991 |
| GFAP | uncinate fasciculus (r) OD | -0.009 (-0.024, 0.006) | 0.236 | 0.518 |
| GFAP | uncinate fasciculus (l) OD | -0.005 (-0.020, 0.010) | 0.532 | 0.708 |
| GFAP | superior thalamic radiation (r) OD | -0.006 (-0.012, 0.000) | 0.036 | 0.187 |
| GFAP | superior thalamic radiation (l) OD | -0.011 (-0.017, -0.005) | 0.001 | 0.034 |
| GFAP | superior longitudinal fasciculus (r) OD | 0.000 (-0.009, 0.009) | 0.966 | 0.981 |
| GFAP | superior longitudinal fasciculus (l) OD | 0.003 (-0.006, 0.012) | 0.507 | 0.689 |
| GFAP | posterior thalamic radiation (r) OD | -0.014 (-0.030, 0.001) | 0.075 | 0.253 |
| GFAP | posterior thalamic radiation (l) OD | 0.007 (-0.009, 0.023) | 0.372 | 0.571 |
| GFAP | parahippocampal part of cingulum (r) OD | -0.014 (-0.029, 0.002) | 0.085 | 0.267 |
| GFAP | parahippocampal part of cingulum (l) OD | -0.011 (-0.026, 0.005) | 0.193 | 0.449 |
| GFAP | middle cerebellar peduncle (l) OD | -0.001 (-0.015, 0.012) | 0.837 | 0.917 |
| GFAP | medial lemniscus (r) OD | -0.014 (-0.025, -0.002) | 0.021 | 0.167 |
| GFAP | medial lemniscus (l) OD | 0.001 (-0.010, 0.012) | 0.859 | 0.917 |
| GFAP | inferior longitudinal fasciculus (r) OD | -0.022 (-0.036, -0.008) | 0.002 | 0.054 |
| GFAP | inferior longitudinal fasciculus (l) OD | -0.013 (-0.027, 0.001) | 0.065 | 0.237 |
| GFAP | inferior fronto-occipital fasciculus (r) OD | -0.019 (-0.032, -0.006) | 0.005 | 0.068 |
| GFAP | inferior fronto-occipital fasciculus (l) OD | -0.008 (-0.022, 0.005) | 0.234 | 0.518 |
| GFAP | forceps major (r) OD | -0.002 (-0.012, 0.009) | 0.757 | 0.891 |
| GFAP | forceps major (l) OD | 0.001 (-0.010, 0.013) | 0.821 | 0.917 |
| GFAP | corticospinal tract (r) OD | -0.008 (-0.018, 0.003) | 0.155 | 0.388 |
| GFAP | corticospinal tract (l) OD | 0.009 (0.000, 0.018) | 0.053 | 0.217 |
| GFAP | cingulate gyrus part of cingulum (r) OD | 0.005 (-0.007, 0.017) | 0.399 | 0.605 |
| GFAP | cingulate gyrus part of cingulum (l) OD | 0.006 (-0.007, 0.019) | 0.334 | 0.565 |
| GFAP | anterior thalamic radiation (r) OD | -0.006 (-0.016, 0.004) | 0.266 | 0.531 |
| GFAP | anterior thalamic radiation (l) OD | -0.009 (-0.019, 0.002) | 0.098 | 0.276 |
| GFAP | acoustic radiation (r) OD | -0.007 (-0.020, 0.006) | 0.283 | 0.531 |
| GFAP | acoustic radiation (l) OD | -0.017 (-0.030, -0.004) | 0.012 | 0.108 |
| NfL | uncinate fasciculus (r) OD | -0.015 (-0.029, 0.000) | 0.053 | 0.152 |
| NfL | uncinate fasciculus (l) OD | -0.015 (-0.030, 0.000) | 0.044 | 0.138 |
| NfL | superior thalamic radiation (r) OD | -0.006 (-0.012, 0.000) | 0.041 | 0.135 |
| NfL | superior thalamic radiation (l) OD | -0.016 (-0.022, -0.010) | 0.000 | 0.000 |
| NfL | superior longitudinal fasciculus (r) OD | -0.003 (-0.012, 0.005) | 0.459 | 0.630 |
| NfL | superior longitudinal fasciculus (l) OD | -0.003 (-0.012, 0.005) | 0.466 | 0.630 |
| NfL | posterior thalamic radiation (r) OD | -0.011 (-0.026, 0.004) | 0.150 | 0.316 |
| NfL | posterior thalamic radiation (l) OD | -0.006 (-0.021, 0.010) | 0.467 | 0.630 |
| NfL | parahippocampal part of cingulum (r) OD | -0.009 (-0.024, 0.006) | 0.264 | 0.463 |
| NfL | parahippocampal part of cingulum (l) OD | -0.016 (-0.031, 0.000) | 0.047 | 0.141 |
| NfL | middle cerebellar peduncle (l) OD | 0.007 (-0.006, 0.021) | 0.287 | 0.478 |
| NfL | medial lemniscus (r) OD | -0.009 (-0.020, 0.002) | 0.121 | 0.297 |
| NfL | medial lemniscus (l) OD | 0.004 (-0.007, 0.015) | 0.489 | 0.641 |
| NfL | inferior longitudinal fasciculus (r) OD | -0.019 (-0.033, -0.005) | 0.007 | 0.053 |
| NfL | inferior longitudinal fasciculus (l) OD | -0.012 (-0.025, 0.002) | 0.085 | 0.225 |
| NfL | inferior fronto-occipital fasciculus (r) OD | -0.011 (-0.023, 0.002) | 0.095 | 0.240 |
| NfL | inferior fronto-occipital fasciculus (l) OD | -0.021 (-0.034, -0.007) | 0.002 | 0.027 |
| NfL | forceps major (r) OD | -0.002 (-0.012, 0.008) | 0.689 | 0.784 |
| NfL | forceps major (l) OD | -0.011 (-0.022, 0.000) | 0.044 | 0.138 |
| NfL | corticospinal tract (r) OD | -0.018 (-0.028, -0.008) | 0.000 | 0.000 |
| NfL | corticospinal tract (l) OD | -0.002 (-0.010, 0.007) | 0.722 | 0.806 |
| NfL | cingulate gyrus part of cingulum (r) OD | 0.003 (-0.009, 0.015) | 0.610 | 0.743 |
| NfL | cingulate gyrus part of cingulum (l) OD | 0.000 (-0.013, 0.012) | 0.986 | 0.986 |
| NfL | anterior thalamic radiation (r) OD | -0.006 (-0.016, 0.004) | 0.252 | 0.462 |
| NfL | anterior thalamic radiation (l) OD | -0.014 (-0.024, -0.004) | 0.007 | 0.053 |
| NfL | acoustic radiation (r) OD | -0.002 (-0.014, 0.011) | 0.775 | 0.837 |
| NfL | acoustic radiation (l) OD | -0.022 (-0.034, -0.009) | 0.001 | 0.023 |
| p-tau181 | uncinate fasciculus (r) OD | -0.011 (-0.025, 0.004) | 0.162 | 0.336 |
| p-tau181 | uncinate fasciculus (l) OD | 0.000 (-0.015, 0.015) | 0.985 | 0.988 |
| p-tau181 | superior thalamic radiation (r) OD | -0.002 (-0.008, 0.004) | 0.486 | 0.669 |
| p-tau181 | superior thalamic radiation (l) OD | -0.004 (-0.010, 0.002) | 0.156 | 0.333 |
| p-tau181 | superior longitudinal fasciculus (r) OD | -0.002 (-0.010, 0.007) | 0.671 | 0.809 |
| p-tau181 | superior longitudinal fasciculus (l) OD | -0.001 (-0.010, 0.007) | 0.781 | 0.879 |
| p-tau181 | posterior thalamic radiation (r) OD | -0.020 (-0.035, -0.005) | 0.008 | 0.152 |
| p-tau181 | posterior thalamic radiation (l) OD | -0.019 (-0.034, -0.004) | 0.015 | 0.153 |
| p-tau181 | parahippocampal part of cingulum (r) OD | -0.023 (-0.038, -0.008) | 0.003 | 0.101 |
| p-tau181 | parahippocampal part of cingulum (l) OD | -0.021 (-0.036, -0.005) | 0.009 | 0.152 |
| p-tau181 | middle cerebellar peduncle (l) OD | -0.004 (-0.017, 0.010) | 0.593 | 0.762 |
| p-tau181 | medial lemniscus (r) OD | -0.005 (-0.017, 0.006) | 0.377 | 0.606 |
| p-tau181 | medial lemniscus (l) OD | -0.002 (-0.013, 0.009) | 0.777 | 0.879 |
| p-tau181 | inferior longitudinal fasciculus (r) OD | -0.016 (-0.030, -0.002) | 0.023 | 0.183 |
| p-tau181 | inferior longitudinal fasciculus (l) OD | -0.017 (-0.030, -0.003) | 0.014 | 0.153 |
| p-tau181 | inferior fronto-occipital fasciculus (r) OD | -0.017 (-0.030, -0.005) | 0.007 | 0.152 |
| p-tau181 | inferior fronto-occipital fasciculus (l) OD | -0.016 (-0.030, -0.003) | 0.016 | 0.153 |
| p-tau181 | forceps major (r) OD | -0.011 (-0.021, -0.001) | 0.025 | 0.188 |
| p-tau181 | forceps major (l) OD | -0.001 (-0.012, 0.010) | 0.864 | 0.926 |
| p-tau181 | corticospinal tract (r) OD | 0.001 (-0.009, 0.011) | 0.859 | 0.926 |
| p-tau181 | corticospinal tract (l) OD | 0.003 (-0.006, 0.012) | 0.472 | 0.664 |
| p-tau181 | cingulate gyrus part of cingulum (r) OD | -0.007 (-0.018, 0.005) | 0.268 | 0.476 |
| p-tau181 | cingulate gyrus part of cingulum (l) OD | 0.005 (-0.008, 0.018) | 0.439 | 0.624 |
| p-tau181 | anterior thalamic radiation (r) OD | -0.003 (-0.013, 0.007) | 0.564 | 0.732 |
| p-tau181 | anterior thalamic radiation (l) OD | -0.017 (-0.027, -0.008) | 0.001 | 0.101 |
| p-tau181 | acoustic radiation (r) OD | -0.002 (-0.014, 0.011) | 0.791 | 0.883 |
| p-tau181 | acoustic radiation (l) OD | -0.005 (-0.018, 0.008) | 0.424 | 0.615 |

Abbreviations: Aβ, amyloid-β; GFAP, glial fibrillary acidic protein; NfL, neurofilament light chain; p-tau181, tau phosphorylated at threonine 181; CI, confidence interval; FA, fractional anisotropy; MD, mean diffusivity; ICVF, intracellular volume fraction; ISOVF, isotropic volume fraction; OD, orientation dispersion; r, right; l, left; FDR, false discovery rates. Model was adjusted for sex, age, ethnicity, townsend deprivation index, assessment center, smoking status, alcohol use, family history of dementia, *APOE* ε4 allele, hypertension, diabetes, cardiovascular arterial disease, history of COVID-19 infection, and total intracranial volume.

# Supplementary Table 15. Associations of longitudinal plasma AD-related biomarkers with longitudinal changes in white matter microstructure by using linear mixed-effects models.

| **Plasma biomarkers** | **White matter microstructure** | **β (95% CI)** | ***P*-value** | ***FDR-***  ***corrected P*** |
| --- | --- | --- | --- | --- |
| Aβ42/40 | uncinate fasciculus (r) FA | 0.002 (-0.015, 0.018) | 0.830 | 0.904 |
| Aβ42/40 | uncinate fasciculus (l) FA | 0.004 (-0.013, 0.021) | 0.643 | 0.768 |
| Aβ42/40 | superior thalamic radiation (r) FA | 0.011 (0.001, 0.022) | 0.036 | 0.116 |
| Aβ42/40 | superior thalamic radiation (l) FA | 0.014 (0.004, 0.024) | 0.008 | 0.057 |
| Aβ42/40 | superior longitudinal fasciculus (r) FA | 0.010 (0.001, 0.020) | 0.029 | 0.100 |
| Aβ42/40 | superior longitudinal fasciculus (l) FA | 0.019 (0.009, 0.028) | 0.000 | 0.000 |
| Aβ42/40 | posterior thalamic radiation (r) FA | 0.000 (-0.014, 0.015) | 0.946 | 0.953 |
| Aβ42/40 | posterior thalamic radiation (l) FA | 0.009 (-0.005, 0.023) | 0.226 | 0.424 |
| Aβ42/40 | parahippocampal part of cingulum (r) FA | 0.003 (-0.016, 0.021) | 0.781 | 0.871 |
| Aβ42/40 | parahippocampal part of cingulum (l) FA | -0.011 (-0.031, 0.009) | 0.279 | 0.445 |
| Aβ42/40 | middle cerebellar peduncle (l) FA | -0.003 (-0.021, 0.015) | 0.759 | 0.857 |
| Aβ42/40 | medial lemniscus (r) FA | 0.029 (0.009, 0.049) | 0.004 | 0.042 |
| Aβ42/40 | medial lemniscus (l) FA | 0.007 (-0.012, 0.026) | 0.473 | 0.650 |
| Aβ42/40 | inferior longitudinal fasciculus (r) FA | 0.003 (-0.008, 0.015) | 0.556 | 0.695 |
| Aβ42/40 | inferior longitudinal fasciculus (l) FA | 0.010 (-0.002, 0.021) | 0.090 | 0.234 |
| Aβ42/40 | inferior fronto-occipital fasciculus (r) FA | 0.002 (-0.009, 0.013) | 0.675 | 0.799 |
| Aβ42/40 | inferior fronto-occipital fasciculus (l) FA | 0.016 (0.004, 0.028) | 0.008 | 0.057 |
| Aβ42/40 | forceps major (r) FA | -0.009 (-0.019, 0.002) | 0.098 | 0.245 |
| Aβ42/40 | forceps major (l) FA | 0.010 (-0.002, 0.022) | 0.107 | 0.245 |
| Aβ42/40 | corticospinal tract (r) FA | 0.005 (-0.010, 0.019) | 0.547 | 0.690 |
| Aβ42/40 | corticospinal tract (l) FA | 0.009 (-0.006, 0.024) | 0.252 | 0.425 |
| Aβ42/40 | cingulate gyrus part of cingulum (r) FA | 0.007 (-0.006, 0.021) | 0.268 | 0.436 |
| Aβ42/40 | cingulate gyrus part of cingulum (l) FA | 0.003 (-0.012, 0.017) | 0.714 | 0.838 |
| Aβ42/40 | anterior thalamic radiation (r) FA | -0.001 (-0.013, 0.011) | 0.844 | 0.905 |
| Aβ42/40 | anterior thalamic radiation (l) FA | 0.001 (-0.012, 0.013) | 0.920 | 0.941 |
| Aβ42/40 | acoustic radiation (r) FA | 0.004 (-0.012, 0.020) | 0.626 | 0.761 |
| Aβ42/40 | acoustic radiation (l) FA | 0.014 (-0.003, 0.031) | 0.102 | 0.245 |
| GFAP | uncinate fasciculus (r) FA | 0.002 (-0.015, 0.018) | 0.820 | 0.873 |
| GFAP | uncinate fasciculus (l) FA | -0.006 (-0.023, 0.012) | 0.506 | 0.638 |
| GFAP | superior thalamic radiation (r) FA | 0.001 (-0.009, 0.012) | 0.821 | 0.873 |
| GFAP | superior thalamic radiation (l) FA | -0.012 (-0.022, -0.001) | 0.025 | 0.113 |
| GFAP | superior longitudinal fasciculus (r) FA | -0.004 (-0.013, 0.006) | 0.432 | 0.598 |
| GFAP | superior longitudinal fasciculus (l) FA | -0.013 (-0.023, -0.003) | 0.009 | 0.061 |
| GFAP | posterior thalamic radiation (r) FA | -0.002 (-0.017, 0.012) | 0.737 | 0.828 |
| GFAP | posterior thalamic radiation (l) FA | -0.009 (-0.023, 0.005) | 0.225 | 0.442 |
| GFAP | parahippocampal part of cingulum (r) FA | -0.015 (-0.033, 0.004) | 0.129 | 0.323 |
| GFAP | parahippocampal part of cingulum (l) FA | -0.003 (-0.023, 0.017) | 0.778 | 0.851 |
| GFAP | middle cerebellar peduncle (l) FA | -0.007 (-0.025, 0.011) | 0.471 | 0.627 |
| GFAP | medial lemniscus (r) FA | -0.011 (-0.031, 0.009) | 0.289 | 0.494 |
| GFAP | medial lemniscus (l) FA | -0.018 (-0.037, 0.001) | 0.070 | 0.216 |
| GFAP | inferior longitudinal fasciculus (r) FA | 0.004 (-0.007, 0.015) | 0.492 | 0.633 |
| GFAP | inferior longitudinal fasciculus (l) FA | -0.004 (-0.015, 0.008) | 0.504 | 0.638 |
| GFAP | inferior fronto-occipital fasciculus (r) FA | 0.006 (-0.005, 0.017) | 0.285 | 0.493 |
| GFAP | inferior fronto-occipital fasciculus (l) FA | 0.006 (-0.006, 0.018) | 0.322 | 0.524 |
| GFAP | forceps major (r) FA | 0.001 (-0.010, 0.011) | 0.876 | 0.903 |
| GFAP | forceps major (l) FA | -0.003 (-0.015, 0.010) | 0.678 | 0.810 |
| GFAP | corticospinal tract (r) FA | 0.015 (0.000, 0.029) | 0.053 | 0.175 |
| GFAP | corticospinal tract (l) FA | -0.005 (-0.020, 0.010) | 0.491 | 0.633 |
| GFAP | cingulate gyrus part of cingulum (r) FA | 0.003 (-0.011, 0.016) | 0.695 | 0.816 |
| GFAP | cingulate gyrus part of cingulum (l) FA | 0.009 (-0.006, 0.023) | 0.252 | 0.466 |
| GFAP | anterior thalamic radiation (r) FA | 0.007 (-0.005, 0.019) | 0.271 | 0.493 |
| GFAP | anterior thalamic radiation (l) FA | 0.007 (-0.006, 0.020) | 0.280 | 0.493 |
| GFAP | acoustic radiation (r) FA | 0.002 (-0.014, 0.018) | 0.811 | 0.873 |
| GFAP | acoustic radiation (l) FA | -0.012 (-0.029, 0.005) | 0.178 | 0.390 |
| NfL | uncinate fasciculus (r) FA | -0.001 (-0.015, 0.013) | 0.912 | 0.944 |
| NfL | uncinate fasciculus (l) FA | -0.001 (-0.016, 0.014) | 0.896 | 0.938 |
| NfL | superior thalamic radiation (r) FA | -0.010 (-0.019, -0.001) | 0.026 | 0.176 |
| NfL | superior thalamic radiation (l) FA | -0.016 (-0.025, -0.007) | 0.000 | 0.000 |
| NfL | superior longitudinal fasciculus (r) FA | -0.016 (-0.025, -0.008) | 0.000 | 0.000 |
| NfL | superior longitudinal fasciculus (l) FA | -0.013 (-0.021, -0.004) | 0.003 | 0.068 |
| NfL | posterior thalamic radiation (r) FA | -0.015 (-0.027, -0.003) | 0.016 | 0.135 |
| NfL | posterior thalamic radiation (l) FA | -0.004 (-0.017, 0.008) | 0.472 | 0.675 |
| NfL | parahippocampal part of cingulum (r) FA | -0.007 (-0.023, 0.008) | 0.362 | 0.575 |
| NfL | parahippocampal part of cingulum (l) FA | -0.008 (-0.025, 0.009) | 0.358 | 0.575 |
| NfL | middle cerebellar peduncle (l) FA | -0.010 (-0.025, 0.006) | 0.219 | 0.531 |
| NfL | medial lemniscus (r) FA | -0.002 (-0.019, 0.015) | 0.859 | 0.906 |
| NfL | medial lemniscus (l) FA | 0.001 (-0.016, 0.017) | 0.939 | 0.946 |
| NfL | inferior longitudinal fasciculus (r) FA | -0.012 (-0.022, -0.002) | 0.014 | 0.126 |
| NfL | inferior longitudinal fasciculus (l) FA | -0.005 (-0.015, 0.004) | 0.281 | 0.566 |
| NfL | inferior fronto-occipital fasciculus (r) FA | -0.007 (-0.016, 0.002) | 0.151 | 0.425 |
| NfL | inferior fronto-occipital fasciculus (l) FA | 0.002 (-0.009, 0.012) | 0.766 | 0.869 |
| NfL | forceps major (r) FA | -0.003 (-0.011, 0.006) | 0.575 | 0.753 |
| NfL | forceps major (l) FA | -0.005 (-0.015, 0.005) | 0.333 | 0.574 |
| NfL | corticospinal tract (r) FA | -0.002 (-0.014, 0.011) | 0.806 | 0.885 |
| NfL | corticospinal tract (l) FA | -0.013 (-0.025, 0.000) | 0.054 | 0.270 |
| NfL | cingulate gyrus part of cingulum (r) FA | -0.002 (-0.013, 0.009) | 0.718 | 0.843 |
| NfL | cingulate gyrus part of cingulum (l) FA | 0.000 (-0.012, 0.013) | 0.996 | 0.996 |
| NfL | anterior thalamic radiation (r) FA | -0.007 (-0.018, 0.003) | 0.165 | 0.455 |
| NfL | anterior thalamic radiation (l) FA | -0.004 (-0.015, 0.007) | 0.517 | 0.701 |
| NfL | acoustic radiation (r) FA | -0.007 (-0.021, 0.007) | 0.349 | 0.574 |
| NfL | acoustic radiation (l) FA | -0.012 (-0.027, 0.002) | 0.101 | 0.310 |
| p-tau181 | uncinate fasciculus (r) FA | 0.006 (-0.011, 0.022) | 0.496 | 0.775 |
| p-tau181 | uncinate fasciculus (l) FA | 0.011 (-0.006, 0.028) | 0.209 | 0.561 |
| p-tau181 | superior thalamic radiation (r) FA | 0.007 (-0.003, 0.018) | 0.165 | 0.558 |
| p-tau181 | superior thalamic radiation (l) FA | 0.004 (-0.006, 0.014) | 0.478 | 0.775 |
| p-tau181 | superior longitudinal fasciculus (r) FA | 0.004 (-0.006, 0.013) | 0.420 | 0.720 |
| p-tau181 | superior longitudinal fasciculus (l) FA | 0.003 (-0.006, 0.013) | 0.490 | 0.775 |
| p-tau181 | posterior thalamic radiation (r) FA | 0.003 (-0.011, 0.017) | 0.676 | 0.837 |
| p-tau181 | posterior thalamic radiation (l) FA | 0.002 (-0.012, 0.016) | 0.780 | 0.849 |
| p-tau181 | parahippocampal part of cingulum (r) FA | 0.006 (-0.012, 0.024) | 0.520 | 0.778 |
| p-tau181 | parahippocampal part of cingulum (l) FA | 0.008 (-0.012, 0.028) | 0.425 | 0.720 |
| p-tau181 | middle cerebellar peduncle (l) FA | 0.010 (-0.008, 0.028) | 0.256 | 0.603 |
| p-tau181 | medial lemniscus (r) FA | -0.004 (-0.023, 0.016) | 0.718 | 0.849 |
| p-tau181 | medial lemniscus (l) FA | -0.002 (-0.021, 0.017) | 0.828 | 0.871 |
| p-tau181 | inferior longitudinal fasciculus (r) FA | 0.008 (-0.003, 0.019) | 0.136 | 0.558 |
| p-tau181 | inferior longitudinal fasciculus (l) FA | -0.001 (-0.012, 0.010) | 0.891 | 0.908 |
| p-tau181 | inferior fronto-occipital fasciculus (r) FA | 0.002 (-0.008, 0.013) | 0.664 | 0.830 |
| p-tau181 | inferior fronto-occipital fasciculus (l) FA | 0.004 (-0.008, 0.015) | 0.557 | 0.807 |
| p-tau181 | forceps major (r) FA | -0.003 (-0.013, 0.007) | 0.588 | 0.818 |
| p-tau181 | forceps major (l) FA | 0.007 (-0.005, 0.018) | 0.259 | 0.603 |
| p-tau181 | corticospinal tract (r) FA | 0.009 (-0.006, 0.023) | 0.229 | 0.583 |
| p-tau181 | corticospinal tract (l) FA | 0.007 (-0.007, 0.022) | 0.332 | 0.700 |
| p-tau181 | cingulate gyrus part of cingulum (r) FA | -0.002 (-0.015, 0.011) | 0.774 | 0.849 |
| p-tau181 | cingulate gyrus part of cingulum (l) FA | 0.010 (-0.005, 0.024) | 0.178 | 0.558 |
| p-tau181 | anterior thalamic radiation (r) FA | 0.005 (-0.007, 0.017) | 0.402 | 0.720 |
| p-tau181 | anterior thalamic radiation (l) FA | 0.002 (-0.011, 0.014) | 0.759 | 0.849 |
| p-tau181 | acoustic radiation (r) FA | 0.011 (-0.005, 0.027) | 0.182 | 0.558 |
| p-tau181 | acoustic radiation (l) FA | -0.005 (-0.022, 0.012) | 0.564 | 0.807 |
| Aβ42/40 | uncinate fasciculus (r) MD | -0.006 (-0.024, 0.011) | 0.474 | 0.650 |
| Aβ42/40 | uncinate fasciculus (l) MD | -0.009 (-0.028, 0.009) | 0.322 | 0.500 |
| Aβ42/40 | superior thalamic radiation (r) MD | 0.003 (-0.014, 0.020) | 0.747 | 0.855 |
| Aβ42/40 | superior thalamic radiation (l) MD | -0.003 (-0.018, 0.013) | 0.731 | 0.851 |
| Aβ42/40 | superior longitudinal fasciculus (r) MD | -0.012 (-0.024, 0.001) | 0.062 | 0.178 |
| Aβ42/40 | superior longitudinal fasciculus (l) MD | -0.022 (-0.033, -0.010) | 0.000 | 0.000 |
| Aβ42/40 | posterior thalamic radiation (r) MD | -0.015 (-0.029, -0.001) | 0.039 | 0.120 |
| Aβ42/40 | posterior thalamic radiation (l) MD | -0.028 (-0.043, -0.014) | 0.000 | 0.000 |
| Aβ42/40 | parahippocampal part of cingulum (r) MD | -0.007 (-0.026, 0.012) | 0.455 | 0.647 |
| Aβ42/40 | parahippocampal part of cingulum (l) MD | -0.013 (-0.033, 0.007) | 0.195 | 0.387 |
| Aβ42/40 | middle cerebellar peduncle (l) MD | -0.015 (-0.035, 0.006) | 0.154 | 0.320 |
| Aβ42/40 | medial lemniscus (r) MD | -0.020 (-0.045, 0.004) | 0.106 | 0.245 |
| Aβ42/40 | medial lemniscus (l) MD | -0.020 (-0.044, 0.005) | 0.122 | 0.266 |
| Aβ42/40 | inferior longitudinal fasciculus (r) MD | -0.021 (-0.036, -0.005) | 0.008 | 0.057 |
| Aβ42/40 | inferior longitudinal fasciculus (l) MD | -0.035 (-0.049, -0.020) | 0.000 | 0.000 |
| Aβ42/40 | inferior fronto-occipital fasciculus (r) MD | -0.007 (-0.020, 0.007) | 0.361 | 0.541 |
| Aβ42/40 | inferior fronto-occipital fasciculus (l) MD | -0.017 (-0.030, -0.003) | 0.018 | 0.081 |
| Aβ42/40 | forceps major (r) MD | 0.012 (-0.001, 0.026) | 0.077 | 0.208 |
| Aβ42/40 | forceps major (l) MD | -0.017 (-0.031, -0.004) | 0.012 | 0.068 |
| Aβ42/40 | corticospinal tract (r) MD | 0.003 (-0.016, 0.023) | 0.739 | 0.853 |
| Aβ42/40 | corticospinal tract (l) MD | -0.009 (-0.029, 0.011) | 0.365 | 0.541 |
| Aβ42/40 | cingulate gyrus part of cingulum (r) MD | 0.005 (-0.011, 0.022) | 0.527 | 0.681 |
| Aβ42/40 | cingulate gyrus part of cingulum (l) MD | 0.000 (-0.018, 0.017) | 0.964 | 0.964 |
| Aβ42/40 | anterior thalamic radiation (r) MD | 0.002 (-0.012, 0.015) | 0.820 | 0.901 |
| Aβ42/40 | anterior thalamic radiation (l) MD | -0.006 (-0.020, 0.009) | 0.431 | 0.626 |
| Aβ42/40 | acoustic radiation (r) MD | -0.013 (-0.033, 0.007) | 0.205 | 0.395 |
| Aβ42/40 | acoustic radiation (l) MD | -0.032 (-0.051, -0.012) | 0.002 | 0.030 |
| GFAP | uncinate fasciculus (r) MD | 0.004 (-0.014, 0.022) | 0.677 | 0.810 |
| GFAP | uncinate fasciculus (l) MD | 0.012 (-0.007, 0.031) | 0.204 | 0.428 |
| GFAP | superior thalamic radiation (r) MD | -0.009 (-0.026, 0.008) | 0.311 | 0.512 |
| GFAP | superior thalamic radiation (l) MD | 0.002 (-0.014, 0.017) | 0.847 | 0.891 |
| GFAP | superior longitudinal fasciculus (r) MD | 0.002 (-0.010, 0.015) | 0.704 | 0.819 |
| GFAP | superior longitudinal fasciculus (l) MD | 0.016 (0.005, 0.028) | 0.006 | 0.051 |
| GFAP | posterior thalamic radiation (r) MD | 0.015 (0.001, 0.029) | 0.032 | 0.131 |
| GFAP | posterior thalamic radiation (l) MD | 0.023 (0.008, 0.038) | 0.002 | 0.027 |
| GFAP | parahippocampal part of cingulum (r) MD | 0.015 (-0.004, 0.034) | 0.116 | 0.295 |
| GFAP | parahippocampal part of cingulum (l) MD | 0.008 (-0.012, 0.028) | 0.416 | 0.585 |
| GFAP | middle cerebellar peduncle (l) MD | 0.012 (-0.008, 0.033) | 0.233 | 0.445 |
| GFAP | medial lemniscus (r) MD | 0.036 (0.011, 0.060) | 0.004 | 0.042 |
| GFAP | medial lemniscus (l) MD | 0.025 (0.000, 0.050) | 0.053 | 0.175 |
| GFAP | inferior longitudinal fasciculus (r) MD | 0.015 (0.000, 0.031) | 0.047 | 0.171 |
| GFAP | inferior longitudinal fasciculus (l) MD | 0.025 (0.010, 0.040) | 0.001 | 0.019 |
| GFAP | inferior fronto-occipital fasciculus (r) MD | 0.010 (-0.004, 0.024) | 0.165 | 0.387 |
| GFAP | inferior fronto-occipital fasciculus (l) MD | 0.021 (0.008, 0.035) | 0.002 | 0.027 |
| GFAP | forceps major (r) MD | 0.009 (-0.005, 0.022) | 0.224 | 0.442 |
| GFAP | forceps major (l) MD | 0.000 (-0.013, 0.014) | 0.953 | 0.967 |
| GFAP | corticospinal tract (r) MD | 0.000 (-0.020, 0.020) | 0.994 | 0.994 |
| GFAP | corticospinal tract (l) MD | 0.006 (-0.014, 0.026) | 0.553 | 0.679 |
| GFAP | cingulate gyrus part of cingulum (r) MD | -0.010 (-0.027, 0.006) | 0.226 | 0.442 |
| GFAP | cingulate gyrus part of cingulum (l) MD | -0.012 (-0.030, 0.005) | 0.167 | 0.387 |
| GFAP | anterior thalamic radiation (r) MD | -0.011 (-0.025, 0.002) | 0.109 | 0.283 |
| GFAP | anterior thalamic radiation (l) MD | -0.005 (-0.020, 0.009) | 0.454 | 0.619 |
| GFAP | acoustic radiation (r) MD | 0.002 (-0.019, 0.022) | 0.869 | 0.902 |
| GFAP | acoustic radiation (l) MD | 0.009 (-0.011, 0.029) | 0.363 | 0.559 |
| NfL | uncinate fasciculus (r) MD | 0.013 (-0.002, 0.028) | 0.085 | 0.296 |
| NfL | uncinate fasciculus (l) MD | 0.010 (-0.006, 0.026) | 0.220 | 0.531 |
| NfL | superior thalamic radiation (r) MD | 0.020 (0.006, 0.035) | 0.006 | 0.081 |
| NfL | superior thalamic radiation (l) MD | 0.019 (0.006, 0.032) | 0.004 | 0.077 |
| NfL | superior longitudinal fasciculus (r) MD | 0.014 (0.004, 0.025) | 0.008 | 0.083 |
| NfL | superior longitudinal fasciculus (l) MD | 0.017 (0.007, 0.027) | 0.001 | 0.034 |
| NfL | posterior thalamic radiation (r) MD | 0.019 (0.007, 0.031) | 0.002 | 0.054 |
| NfL | posterior thalamic radiation (l) MD | 0.012 (-0.001, 0.024) | 0.076 | 0.296 |
| NfL | parahippocampal part of cingulum (r) MD | 0.004 (-0.012, 0.020) | 0.620 | 0.768 |
| NfL | parahippocampal part of cingulum (l) MD | 0.011 (-0.006, 0.027) | 0.216 | 0.531 |
| NfL | middle cerebellar peduncle (l) MD | 0.019 (0.001, 0.036) | 0.036 | 0.208 |
| NfL | medial lemniscus (r) MD | 0.013 (-0.008, 0.034) | 0.225 | 0.531 |
| NfL | medial lemniscus (l) MD | 0.010 (-0.011, 0.031) | 0.352 | 0.574 |
| NfL | inferior longitudinal fasciculus (r) MD | 0.023 (0.010, 0.036) | 0.001 | 0.034 |
| NfL | inferior longitudinal fasciculus (l) MD | 0.016 (0.004, 0.029) | 0.011 | 0.106 |
| NfL | inferior fronto-occipital fasciculus (r) MD | 0.013 (0.001, 0.025) | 0.037 | 0.208 |
| NfL | inferior fronto-occipital fasciculus (l) MD | 0.013 (0.002, 0.025) | 0.026 | 0.176 |
| NfL | forceps major (r) MD | 0.011 (0.000, 0.023) | 0.060 | 0.279 |
| NfL | forceps major (l) MD | 0.003 (-0.009, 0.015) | 0.597 | 0.760 |
| NfL | corticospinal tract (r) MD | -0.002 (-0.019, 0.015) | 0.793 | 0.878 |
| NfL | corticospinal tract (l) MD | 0.007 (-0.010, 0.024) | 0.437 | 0.656 |
| NfL | cingulate gyrus part of cingulum (r) MD | 0.004 (-0.010, 0.018) | 0.588 | 0.756 |
| NfL | cingulate gyrus part of cingulum (l) MD | 0.009 (-0.006, 0.024) | 0.223 | 0.531 |
| NfL | anterior thalamic radiation (r) MD | 0.012 (0.000, 0.023) | 0.044 | 0.238 |
| NfL | anterior thalamic radiation (l) MD | 0.012 (0.000, 0.024) | 0.049 | 0.254 |
| NfL | acoustic radiation (r) MD | 0.002 (-0.015, 0.019) | 0.829 | 0.888 |
| NfL | acoustic radiation (l) MD | 0.007 (-0.010, 0.023) | 0.442 | 0.656 |
| p-tau181 | uncinate fasciculus (r) MD | -0.017 (-0.035, 0.000) | 0.049 | 0.558 |
| p-tau181 | uncinate fasciculus (l) MD | -0.010 (-0.028, 0.008) | 0.287 | 0.644 |
| p-tau181 | superior thalamic radiation (r) MD | -0.004 (-0.020, 0.013) | 0.650 | 0.825 |
| p-tau181 | superior thalamic radiation (l) MD | -0.004 (-0.019, 0.011) | 0.618 | 0.819 |
| p-tau181 | superior longitudinal fasciculus (r) MD | -0.009 (-0.021, 0.003) | 0.123 | 0.558 |
| p-tau181 | superior longitudinal fasciculus (l) MD | -0.008 (-0.020, 0.003) | 0.143 | 0.558 |
| p-tau181 | posterior thalamic radiation (r) MD | -0.010 (-0.024, 0.004) | 0.149 | 0.558 |
| p-tau181 | posterior thalamic radiation (l) MD | -0.014 (-0.029, 0.001) | 0.062 | 0.558 |
| p-tau181 | parahippocampal part of cingulum (r) MD | -0.017 (-0.036, 0.002) | 0.076 | 0.558 |
| p-tau181 | parahippocampal part of cingulum (l) MD | -0.031 (-0.050, -0.012) | 0.002 | 0.203 |
| p-tau181 | middle cerebellar peduncle (l) MD | -0.015 (-0.035, 0.005) | 0.146 | 0.558 |
| p-tau181 | medial lemniscus (r) MD | -0.018 (-0.042, 0.006) | 0.136 | 0.558 |
| p-tau181 | medial lemniscus (l) MD | -0.003 (-0.027, 0.022) | 0.832 | 0.871 |
| p-tau181 | inferior longitudinal fasciculus (r) MD | -0.011 (-0.026, 0.004) | 0.137 | 0.558 |
| p-tau181 | inferior longitudinal fasciculus (l) MD | -0.017 (-0.031, -0.002) | 0.025 | 0.558 |
| p-tau181 | inferior fronto-occipital fasciculus (r) MD | -0.015 (-0.029, -0.002) | 0.028 | 0.558 |
| p-tau181 | inferior fronto-occipital fasciculus (l) MD | -0.011 (-0.025, 0.002) | 0.098 | 0.558 |
| p-tau181 | forceps major (r) MD | -0.003 (-0.016, 0.011) | 0.701 | 0.849 |
| p-tau181 | forceps major (l) MD | -0.007 (-0.021, 0.006) | 0.303 | 0.649 |
| p-tau181 | corticospinal tract (r) MD | -0.011 (-0.030, 0.009) | 0.291 | 0.644 |
| p-tau181 | corticospinal tract (l) MD | -0.009 (-0.028, 0.011) | 0.372 | 0.713 |
| p-tau181 | cingulate gyrus part of cingulum (r) MD | -0.014 (-0.030, 0.003) | 0.099 | 0.558 |
| p-tau181 | cingulate gyrus part of cingulum (l) MD | -0.008 (-0.025, 0.009) | 0.382 | 0.713 |
| p-tau181 | anterior thalamic radiation (r) MD | -0.014 (-0.027, -0.001) | 0.038 | 0.558 |
| p-tau181 | anterior thalamic radiation (l) MD | -0.010 (-0.024, 0.004) | 0.164 | 0.558 |
| p-tau181 | acoustic radiation (r) MD | -0.013 (-0.033, 0.007) | 0.191 | 0.561 |
| p-tau181 | acoustic radiation (l) MD | -0.004 (-0.023, 0.015) | 0.683 | 0.838 |
| Aβ42/40 | uncinate fasciculus (r) ICVF | 0.027 (0.008, 0.046) | 0.005 | 0.048 |
| Aβ42/40 | uncinate fasciculus (l) ICVF | 0.028 (0.009, 0.047) | 0.003 | 0.034 |
| Aβ42/40 | superior thalamic radiation (r) ICVF | 0.007 (-0.009, 0.022) | 0.412 | 0.605 |
| Aβ42/40 | superior thalamic radiation (l) ICVF | 0.005 (-0.011, 0.020) | 0.545 | 0.690 |
| Aβ42/40 | superior longitudinal fasciculus (r) ICVF | 0.016 (0.004, 0.028) | 0.010 | 0.068 |
| Aβ42/40 | superior longitudinal fasciculus (l) ICVF | 0.015 (0.003, 0.027) | 0.013 | 0.068 |
| Aβ42/40 | posterior thalamic radiation (r) ICVF | 0.013 (-0.003, 0.029) | 0.104 | 0.245 |
| Aβ42/40 | posterior thalamic radiation (l) ICVF | 0.020 (0.004, 0.035) | 0.014 | 0.070 |
| Aβ42/40 | parahippocampal part of cingulum (r) ICVF | 0.027 (0.006, 0.048) | 0.012 | 0.068 |
| Aβ42/40 | parahippocampal part of cingulum (l) ICVF | 0.018 (-0.003, 0.039) | 0.094 | 0.239 |
| Aβ42/40 | middle cerebellar peduncle (l) ICVF | 0.020 (0.002, 0.038) | 0.031 | 0.105 |
| Aβ42/40 | medial lemniscus (r) ICVF | 0.031 (0.008, 0.053) | 0.007 | 0.057 |
| Aβ42/40 | medial lemniscus (l) ICVF | 0.038 (0.015, 0.060) | 0.001 | 0.017 |
| Aβ42/40 | inferior longitudinal fasciculus (r) ICVF | 0.019 (0.004, 0.035) | 0.015 | 0.072 |
| Aβ42/40 | inferior longitudinal fasciculus (l) ICVF | 0.017 (0.001, 0.032) | 0.032 | 0.105 |
| Aβ42/40 | inferior fronto-occipital fasciculus (r) ICVF | 0.014 (0.000, 0.028) | 0.054 | 0.162 |
| Aβ42/40 | inferior fronto-occipital fasciculus (l) ICVF | 0.016 (0.002, 0.031) | 0.027 | 0.099 |
| Aβ42/40 | forceps major (r) ICVF | 0.015 (0.002, 0.029) | 0.026 | 0.098 |
| Aβ42/40 | forceps major (l) ICVF | 0.014 (-0.001, 0.030) | 0.071 | 0.200 |
| Aβ42/40 | corticospinal tract (r) ICVF | 0.009 (-0.008, 0.026) | 0.284 | 0.446 |
| Aβ42/40 | corticospinal tract (l) ICVF | 0.008 (-0.008, 0.025) | 0.332 | 0.504 |
| Aβ42/40 | cingulate gyrus part of cingulum (r) ICVF | 0.019 (0.002, 0.036) | 0.025 | 0.098 |
| Aβ42/40 | cingulate gyrus part of cingulum (l) ICVF | 0.013 (-0.003, 0.030) | 0.113 | 0.254 |
| Aβ42/40 | anterior thalamic radiation (r) ICVF | 0.020 (0.004, 0.036) | 0.013 | 0.068 |
| Aβ42/40 | anterior thalamic radiation (l) ICVF | 0.019 (0.003, 0.034) | 0.020 | 0.084 |
| Aβ42/40 | acoustic radiation (r) ICVF | 0.025 (0.007, 0.042) | 0.006 | 0.054 |
| Aβ42/40 | acoustic radiation (l) ICVF | 0.022 (0.005, 0.039) | 0.012 | 0.068 |
| GFAP | uncinate fasciculus (r) ICVF | -0.025 (-0.045, -0.006) | 0.010 | 0.064 |
| GFAP | uncinate fasciculus (l) ICVF | -0.034 (-0.053, -0.015) | 0.000 | 0.000 |
| GFAP | superior thalamic radiation (r) ICVF | -0.011 (-0.027, 0.005) | 0.190 | 0.407 |
| GFAP | superior thalamic radiation (l) ICVF | -0.013 (-0.029, 0.002) | 0.095 | 0.257 |
| GFAP | superior longitudinal fasciculus (r) ICVF | -0.015 (-0.028, -0.003) | 0.017 | 0.092 |
| GFAP | superior longitudinal fasciculus (l) ICVF | -0.014 (-0.026, -0.002) | 0.019 | 0.099 |
| GFAP | posterior thalamic radiation (r) ICVF | -0.019 (-0.035, -0.003) | 0.020 | 0.100 |
| GFAP | posterior thalamic radiation (l) ICVF | -0.022 (-0.037, -0.006) | 0.008 | 0.057 |
| GFAP | parahippocampal part of cingulum (r) ICVF | -0.031 (-0.052, -0.009) | 0.005 | 0.048 |
| GFAP | parahippocampal part of cingulum (l) ICVF | -0.040 (-0.061, -0.018) | 0.000 | 0.000 |
| GFAP | middle cerebellar peduncle (l) ICVF | -0.035 (-0.053, -0.016) | 0.000 | 0.000 |
| GFAP | medial lemniscus (r) ICVF | -0.035 (-0.057, -0.012) | 0.003 | 0.034 |
| GFAP | medial lemniscus (l) ICVF | -0.027 (-0.051, -0.004) | 0.021 | 0.101 |
| GFAP | inferior longitudinal fasciculus (r) ICVF | -0.021 (-0.037, -0.005) | 0.008 | 0.057 |
| GFAP | inferior longitudinal fasciculus (l) ICVF | -0.021 (-0.036, -0.005) | 0.008 | 0.057 |
| GFAP | inferior fronto-occipital fasciculus (r) ICVF | -0.018 (-0.032, -0.003) | 0.017 | 0.092 |
| GFAP | inferior fronto-occipital fasciculus (l) ICVF | -0.024 (-0.039, -0.010) | 0.001 | 0.019 |
| GFAP | forceps major (r) ICVF | -0.014 (-0.027, 0.000) | 0.044 | 0.170 |
| GFAP | forceps major (l) ICVF | -0.024 (-0.040, -0.009) | 0.003 | 0.034 |
| GFAP | corticospinal tract (r) ICVF | -0.012 (-0.029, 0.005) | 0.169 | 0.387 |
| GFAP | corticospinal tract (l) ICVF | -0.012 (-0.029, 0.005) | 0.179 | 0.390 |
| GFAP | cingulate gyrus part of cingulum (r) ICVF | -0.010 (-0.027, 0.007) | 0.234 | 0.445 |
| GFAP | cingulate gyrus part of cingulum (l) ICVF | -0.014 (-0.030, 0.003) | 0.107 | 0.283 |
| GFAP | anterior thalamic radiation (r) ICVF | -0.014 (-0.030, 0.002) | 0.089 | 0.245 |
| GFAP | anterior thalamic radiation (l) ICVF | -0.018 (-0.033, -0.002) | 0.029 | 0.122 |
| GFAP | acoustic radiation (r) ICVF | -0.028 (-0.046, -0.011) | 0.002 | 0.027 |
| GFAP | acoustic radiation (l) ICVF | -0.016 (-0.033, 0.002) | 0.077 | 0.221 |
| NfL | uncinate fasciculus (r) ICVF | -0.007 (-0.024, 0.009) | 0.371 | 0.582 |
| NfL | uncinate fasciculus (l) ICVF | -0.010 (-0.026, 0.006) | 0.232 | 0.531 |
| NfL | superior thalamic radiation (r) ICVF | -0.016 (-0.030, -0.003) | 0.018 | 0.135 |
| NfL | superior thalamic radiation (l) ICVF | -0.016 (-0.029, -0.003) | 0.017 | 0.135 |
| NfL | superior longitudinal fasciculus (r) ICVF | -0.012 (-0.022, -0.001) | 0.030 | 0.193 |
| NfL | superior longitudinal fasciculus (l) ICVF | -0.008 (-0.018, 0.002) | 0.126 | 0.362 |
| NfL | posterior thalamic radiation (r) ICVF | -0.012 (-0.025, 0.002) | 0.095 | 0.298 |
| NfL | posterior thalamic radiation (l) ICVF | -0.004 (-0.017, 0.010) | 0.605 | 0.763 |
| NfL | parahippocampal part of cingulum (r) ICVF | -0.007 (-0.025, 0.011) | 0.453 | 0.658 |
| NfL | parahippocampal part of cingulum (l) ICVF | -0.017 (-0.035, 0.001) | 0.069 | 0.295 |
| NfL | middle cerebellar peduncle (l) ICVF | -0.012 (-0.028, 0.003) | 0.124 | 0.362 |
| NfL | medial lemniscus (r) ICVF | -0.003 (-0.022, 0.016) | 0.751 | 0.859 |
| NfL | medial lemniscus (l) ICVF | -0.005 (-0.024, 0.015) | 0.646 | 0.788 |
| NfL | inferior longitudinal fasciculus (r) ICVF | -0.012 (-0.025, 0.001) | 0.081 | 0.296 |
| NfL | inferior longitudinal fasciculus (l) ICVF | -0.008 (-0.021, 0.005) | 0.223 | 0.531 |
| NfL | inferior fronto-occipital fasciculus (r) ICVF | -0.005 (-0.017, 0.007) | 0.413 | 0.634 |
| NfL | inferior fronto-occipital fasciculus (l) ICVF | -0.009 (-0.021, 0.004) | 0.176 | 0.475 |
| NfL | forceps major (r) ICVF | -0.001 (-0.013, 0.010) | 0.819 | 0.885 |
| NfL | forceps major (l) ICVF | -0.011 (-0.024, 0.003) | 0.123 | 0.362 |
| NfL | corticospinal tract (r) ICVF | -0.013 (-0.027, 0.002) | 0.091 | 0.296 |
| NfL | corticospinal tract (l) ICVF | -0.012 (-0.027, 0.002) | 0.090 | 0.296 |
| NfL | cingulate gyrus part of cingulum (r) ICVF | -0.006 (-0.020, 0.008) | 0.401 | 0.622 |
| NfL | cingulate gyrus part of cingulum (l) ICVF | -0.004 (-0.018, 0.010) | 0.574 | 0.753 |
| NfL | anterior thalamic radiation (r) ICVF | -0.008 (-0.021, 0.006) | 0.267 | 0.565 |
| NfL | anterior thalamic radiation (l) ICVF | -0.012 (-0.025, 0.002) | 0.083 | 0.296 |
| NfL | acoustic radiation (r) ICVF | -0.009 (-0.024, 0.006) | 0.253 | 0.560 |
| NfL | acoustic radiation (l) ICVF | -0.007 (-0.022, 0.007) | 0.334 | 0.574 |
| p-tau181 | uncinate fasciculus (r) ICVF | 0.020 (0.001, 0.039) | 0.037 | 0.558 |
| p-tau181 | uncinate fasciculus (l) ICVF | 0.017 (-0.002, 0.035) | 0.072 | 0.558 |
| p-tau181 | superior thalamic radiation (r) ICVF | 0.002 (-0.014, 0.017) | 0.832 | 0.871 |
| p-tau181 | superior thalamic radiation (l) ICVF | -0.001 (-0.016, 0.014) | 0.899 | 0.908 |
| p-tau181 | superior longitudinal fasciculus (r) ICVF | 0.004 (-0.008, 0.016) | 0.522 | 0.778 |
| p-tau181 | superior longitudinal fasciculus (l) ICVF | 0.003 (-0.008, 0.015) | 0.586 | 0.818 |
| p-tau181 | posterior thalamic radiation (r) ICVF | 0.006 (-0.010, 0.021) | 0.468 | 0.770 |
| p-tau181 | posterior thalamic radiation (l) ICVF | 0.009 (-0.006, 0.025) | 0.252 | 0.603 |
| p-tau181 | parahippocampal part of cingulum (r) ICVF | 0.017 (-0.004, 0.037) | 0.116 | 0.558 |
| p-tau181 | parahippocampal part of cingulum (l) ICVF | 0.010 (-0.011, 0.031) | 0.352 | 0.709 |
| p-tau181 | middle cerebellar peduncle (l) ICVF | 0.012 (-0.006, 0.030) | 0.193 | 0.561 |
| p-tau181 | medial lemniscus (r) ICVF | 0.006 (-0.017, 0.028) | 0.628 | 0.819 |
| p-tau181 | medial lemniscus (l) ICVF | 0.011 (-0.012, 0.034) | 0.349 | 0.709 |
| p-tau181 | inferior longitudinal fasciculus (r) ICVF | 0.007 (-0.009, 0.022) | 0.381 | 0.713 |
| p-tau181 | inferior longitudinal fasciculus (l) ICVF | 0.006 (-0.009, 0.021) | 0.418 | 0.720 |
| p-tau181 | inferior fronto-occipital fasciculus (r) ICVF | 0.010 (-0.004, 0.024) | 0.150 | 0.558 |
| p-tau181 | inferior fronto-occipital fasciculus (l) ICVF | 0.004 (-0.011, 0.018) | 0.613 | 0.819 |
| p-tau181 | forceps major (r) ICVF | 0.009 (-0.005, 0.022) | 0.201 | 0.561 |
| p-tau181 | forceps major (l) ICVF | -0.002 (-0.018, 0.013) | 0.792 | 0.855 |
| p-tau181 | corticospinal tract (r) ICVF | 0.008 (-0.009, 0.025) | 0.352 | 0.709 |
| p-tau181 | corticospinal tract (l) ICVF | 0.003 (-0.014, 0.020) | 0.717 | 0.849 |
| p-tau181 | cingulate gyrus part of cingulum (r) ICVF | 0.004 (-0.012, 0.021) | 0.607 | 0.819 |
| p-tau181 | cingulate gyrus part of cingulum (l) ICVF | 0.006 (-0.010, 0.023) | 0.432 | 0.720 |
| p-tau181 | anterior thalamic radiation (r) ICVF | 0.016 (0.001, 0.032) | 0.043 | 0.558 |
| p-tau181 | anterior thalamic radiation (l) ICVF | 0.009 (-0.006, 0.025) | 0.240 | 0.600 |
| p-tau181 | acoustic radiation (r) ICVF | 0.011 (-0.006, 0.029) | 0.212 | 0.561 |
| p-tau181 | acoustic radiation (l) ICVF | 0.004 (-0.013, 0.021) | 0.631 | 0.819 |
| Aβ42/40 | uncinate fasciculus (r) ISOVF | -0.044 (-0.063, -0.025) | 0.000 | 0.000 |
| Aβ42/40 | uncinate fasciculus (l) ISOVF | 0.034 (0.014, 0.054) | 0.001 | 0.017 |
| Aβ42/40 | superior thalamic radiation (r) ISOVF | -0.028 (-0.044, -0.012) | 0.001 | 0.017 |
| Aβ42/40 | superior thalamic radiation (l) ISOVF | -0.027 (-0.045, -0.009) | 0.003 | 0.034 |
| Aβ42/40 | superior longitudinal fasciculus (r) ISOVF | -0.027 (-0.049, -0.005) | 0.017 | 0.079 |
| Aβ42/40 | superior longitudinal fasciculus (l) ISOVF | 0.025 (0.004, 0.046) | 0.019 | 0.083 |
| Aβ42/40 | posterior thalamic radiation (r) ISOVF | 0.022 (0.001, 0.042) | 0.039 | 0.120 |
| Aβ42/40 | posterior thalamic radiation (l) ISOVF | -0.015 (-0.030, 0.001) | 0.059 | 0.173 |
| Aβ42/40 | parahippocampal part of cingulum (r) ISOVF | -0.015 (-0.033, 0.002) | 0.088 | 0.233 |
| Aβ42/40 | parahippocampal part of cingulum (l) ISOVF | -0.015 (-0.033, 0.003) | 0.102 | 0.245 |
| Aβ42/40 | middle cerebellar peduncle (l) ISOVF | -0.011 (-0.029, 0.007) | 0.220 | 0.418 |
| Aβ42/40 | medial lemniscus (r) ISOVF | 0.013 (-0.009, 0.035) | 0.234 | 0.425 |
| Aβ42/40 | medial lemniscus (l) ISOVF | -0.014 (-0.039, 0.010) | 0.254 | 0.425 |
| Aβ42/40 | inferior longitudinal fasciculus (r) ISOVF | -0.011 (-0.030, 0.008) | 0.255 | 0.425 |
| Aβ42/40 | inferior longitudinal fasciculus (l) ISOVF | -0.011 (-0.030, 0.008) | 0.255 | 0.425 |
| Aβ42/40 | inferior fronto-occipital fasciculus (r) ISOVF | -0.011 (-0.030, 0.008) | 0.255 | 0.425 |
| Aβ42/40 | inferior fronto-occipital fasciculus (l) ISOVF | -0.011 (-0.030, 0.008) | 0.255 | 0.425 |
| Aβ42/40 | forceps major (r) ISOVF | 0.011 (-0.008, 0.031) | 0.260 | 0.428 |
| Aβ42/40 | forceps major (l) ISOVF | -0.012 (-0.036, 0.012) | 0.331 | 0.504 |
| Aβ42/40 | corticospinal tract (r) ISOVF | -0.008 (-0.029, 0.013) | 0.455 | 0.647 |
| Aβ42/40 | corticospinal tract (l) ISOVF | 0.007 (-0.013, 0.028) | 0.488 | 0.652 |
| Aβ42/40 | cingulate gyrus part of cingulum (r) ISOVF | -0.005 (-0.021, 0.011) | 0.511 | 0.676 |
| Aβ42/40 | cingulate gyrus part of cingulum (l) ISOVF | -0.006 (-0.031, 0.019) | 0.636 | 0.767 |
| Aβ42/40 | anterior thalamic radiation (r) ISOVF | 0.003 (-0.018, 0.025) | 0.762 | 0.857 |
| Aβ42/40 | anterior thalamic radiation (l) ISOVF | 0.002 (-0.021, 0.026) | 0.856 | 0.910 |
| Aβ42/40 | acoustic radiation (r) ISOVF | -0.001 (-0.022, 0.020) | 0.897 | 0.932 |
| Aβ42/40 | acoustic radiation (l) ISOVF | 0.001 (-0.018, 0.019) | 0.945 | 0.953 |
| GFAP | uncinate fasciculus (r) ISOVF | -0.034 (-0.055, -0.013) | 0.001 | 0.019 |
| GFAP | uncinate fasciculus (l) ISOVF | -0.029 (-0.049, -0.008) | 0.006 | 0.051 |
| GFAP | superior thalamic radiation (r) ISOVF | -0.024 (-0.046, -0.002) | 0.033 | 0.131 |
| GFAP | superior thalamic radiation (l) ISOVF | 0.020 (0.000, 0.039) | 0.046 | 0.171 |
| GFAP | superior longitudinal fasciculus (r) ISOVF | 0.016 (0.000, 0.032) | 0.050 | 0.173 |
| GFAP | superior longitudinal fasciculus (l) ISOVF | -0.019 (-0.040, 0.002) | 0.071 | 0.216 |
| GFAP | posterior thalamic radiation (r) ISOVF | -0.020 (-0.042, 0.002) | 0.072 | 0.216 |
| GFAP | posterior thalamic radiation (l) ISOVF | 0.016 (-0.002, 0.034) | 0.072 | 0.216 |
| GFAP | parahippocampal part of cingulum (r) ISOVF | 0.022 (-0.002, 0.046) | 0.076 | 0.221 |
| GFAP | parahippocampal part of cingulum (l) ISOVF | 0.012 (-0.006, 0.030) | 0.177 | 0.390 |
| GFAP | middle cerebellar peduncle (l) ISOVF | -0.009 (-0.024, 0.007) | 0.294 | 0.496 |
| GFAP | medial lemniscus (r) ISOVF | -0.008 (-0.023, 0.008) | 0.326 | 0.524 |
| GFAP | medial lemniscus (l) ISOVF | -0.009 (-0.028, 0.011) | 0.379 | 0.559 |
| GFAP | inferior longitudinal fasciculus (r) ISOVF | -0.009 (-0.028, 0.011) | 0.379 | 0.559 |
| GFAP | inferior longitudinal fasciculus (l) ISOVF | -0.009 (-0.028, 0.011) | 0.379 | 0.559 |
| GFAP | inferior fronto-occipital fasciculus (r) ISOVF | -0.009 (-0.028, 0.011) | 0.379 | 0.559 |
| GFAP | inferior fronto-occipital fasciculus (l) ISOVF | -0.011 (-0.035, 0.013) | 0.381 | 0.559 |
| GFAP | forceps major (r) ISOVF | 0.011 (-0.014, 0.036) | 0.392 | 0.563 |
| GFAP | forceps major (l) ISOVF | -0.008 (-0.030, 0.013) | 0.434 | 0.598 |
| GFAP | corticospinal tract (r) ISOVF | -0.008 (-0.028, 0.013) | 0.461 | 0.622 |
| GFAP | corticospinal tract (l) ISOVF | 0.007 (-0.013, 0.027) | 0.474 | 0.627 |
| GFAP | cingulate gyrus part of cingulum (r) ISOVF | 0.009 (-0.016, 0.034) | 0.481 | 0.630 |
| GFAP | cingulate gyrus part of cingulum (l) ISOVF | 0.006 (-0.012, 0.024) | 0.523 | 0.654 |
| GFAP | anterior thalamic radiation (r) ISOVF | -0.003 (-0.021, 0.015) | 0.736 | 0.828 |
| GFAP | anterior thalamic radiation (l) ISOVF | 0.004 (-0.017, 0.025) | 0.738 | 0.828 |
| GFAP | acoustic radiation (r) ISOVF | 0.003 (-0.019, 0.025) | 0.782 | 0.851 |
| GFAP | acoustic radiation (l) ISOVF | 0.001 (-0.017, 0.019) | 0.925 | 0.946 |
| NfL | uncinate fasciculus (r) ISOVF | 0.021 (0.006, 0.036) | 0.006 | 0.081 |
| NfL | uncinate fasciculus (l) ISOVF | 0.021 (0.005, 0.036) | 0.008 | 0.083 |
| NfL | superior thalamic radiation (r) ISOVF | 0.018 (0.005, 0.032) | 0.008 | 0.083 |
| NfL | superior thalamic radiation (l) ISOVF | 0.016 (-0.001, 0.032) | 0.059 | 0.279 |
| NfL | superior longitudinal fasciculus (r) ISOVF | 0.015 (-0.001, 0.030) | 0.065 | 0.293 |
| NfL | superior longitudinal fasciculus (l) ISOVF | 0.014 (-0.001, 0.029) | 0.070 | 0.295 |
| NfL | posterior thalamic radiation (r) ISOVF | 0.016 (-0.002, 0.034) | 0.083 | 0.296 |
| NfL | posterior thalamic radiation (l) ISOVF | 0.015 (-0.002, 0.032) | 0.087 | 0.296 |
| NfL | parahippocampal part of cingulum (r) ISOVF | 0.015 (-0.002, 0.032) | 0.092 | 0.296 |
| NfL | parahippocampal part of cingulum (l) ISOVF | 0.008 (-0.005, 0.022) | 0.230 | 0.531 |
| NfL | middle cerebellar peduncle (l) ISOVF | 0.008 (-0.007, 0.024) | 0.281 | 0.566 |
| NfL | medial lemniscus (r) ISOVF | 0.009 (-0.008, 0.025) | 0.300 | 0.570 |
| NfL | medial lemniscus (l) ISOVF | 0.009 (-0.008, 0.025) | 0.300 | 0.570 |
| NfL | inferior longitudinal fasciculus (r) ISOVF | 0.009 (-0.008, 0.025) | 0.300 | 0.570 |
| NfL | inferior longitudinal fasciculus (l) ISOVF | 0.009 (-0.008, 0.025) | 0.300 | 0.570 |
| NfL | inferior fronto-occipital fasciculus (r) ISOVF | 0.010 (-0.011, 0.030) | 0.353 | 0.574 |
| NfL | inferior fronto-occipital fasciculus (l) ISOVF | -0.008 (-0.028, 0.012) | 0.447 | 0.656 |
| NfL | forceps major (r) ISOVF | -0.006 (-0.024, 0.012) | 0.516 | 0.701 |
| NfL | forceps major (l) ISOVF | 0.006 (-0.015, 0.027) | 0.579 | 0.753 |
| NfL | corticospinal tract (r) ISOVF | 0.006 (-0.015, 0.027) | 0.580 | 0.753 |
| NfL | corticospinal tract (l) ISOVF | -0.004 (-0.023, 0.014) | 0.654 | 0.788 |
| NfL | cingulate gyrus part of cingulum (r) ISOVF | 0.004 (-0.015, 0.023) | 0.668 | 0.798 |
| NfL | cingulate gyrus part of cingulum (l) ISOVF | 0.003 (-0.014, 0.020) | 0.703 | 0.833 |
| NfL | anterior thalamic radiation (r) ISOVF | 0.003 (-0.015, 0.021) | 0.732 | 0.852 |
| NfL | anterior thalamic radiation (l) ISOVF | 0.003 (-0.015, 0.020) | 0.778 | 0.875 |
| NfL | acoustic radiation (r) ISOVF | -0.001 (-0.014, 0.012) | 0.920 | 0.944 |
| NfL | acoustic radiation (l) ISOVF | 0.001 (-0.018, 0.020) | 0.929 | 0.944 |
| p-tau181 | uncinate fasciculus (r) ISOVF | -0.031 (-0.052, -0.011) | 0.003 | 0.203 |
| p-tau181 | uncinate fasciculus (l) ISOVF | -0.018 (-0.035, 0.000) | 0.047 | 0.558 |
| p-tau181 | superior thalamic radiation (r) ISOVF | -0.015 (-0.031, 0.001) | 0.059 | 0.558 |
| p-tau181 | superior thalamic radiation (l) ISOVF | -0.018 (-0.037, 0.001) | 0.066 | 0.558 |
| p-tau181 | superior longitudinal fasciculus (r) ISOVF | -0.014 (-0.029, 0.002) | 0.086 | 0.558 |
| p-tau181 | superior longitudinal fasciculus (l) ISOVF | -0.016 (-0.035, 0.002) | 0.087 | 0.558 |
| p-tau181 | posterior thalamic radiation (r) ISOVF | -0.016 (-0.035, 0.002) | 0.087 | 0.558 |
| p-tau181 | posterior thalamic radiation (l) ISOVF | -0.016 (-0.035, 0.002) | 0.087 | 0.558 |
| p-tau181 | parahippocampal part of cingulum (r) ISOVF | -0.016 (-0.035, 0.002) | 0.087 | 0.558 |
| p-tau181 | parahippocampal part of cingulum (l) ISOVF | -0.019 (-0.043, 0.004) | 0.111 | 0.558 |
| p-tau181 | middle cerebellar peduncle (l) ISOVF | -0.012 (-0.030, 0.005) | 0.164 | 0.558 |
| p-tau181 | medial lemniscus (r) ISOVF | -0.014 (-0.035, 0.006) | 0.170 | 0.558 |
| p-tau181 | medial lemniscus (l) ISOVF | -0.012 (-0.030, 0.005) | 0.170 | 0.558 |
| p-tau181 | inferior longitudinal fasciculus (r) ISOVF | -0.012 (-0.030, 0.005) | 0.175 | 0.558 |
| p-tau181 | inferior longitudinal fasciculus (l) ISOVF | -0.009 (-0.024, 0.006) | 0.224 | 0.582 |
| p-tau181 | inferior fronto-occipital fasciculus (r) ISOVF | -0.010 (-0.027, 0.008) | 0.273 | 0.625 |
| p-tau181 | inferior fronto-occipital fasciculus (l) ISOVF | -0.011 (-0.034, 0.013) | 0.361 | 0.713 |
| p-tau181 | forceps major (r) ISOVF | -0.010 (-0.031, 0.012) | 0.376 | 0.713 |
| p-tau181 | forceps major (l) ISOVF | -0.009 (-0.029, 0.011) | 0.391 | 0.713 |
| p-tau181 | corticospinal tract (r) ISOVF | -0.008 (-0.028, 0.011) | 0.414 | 0.720 |
| p-tau181 | corticospinal tract (l) ISOVF | -0.008 (-0.029, 0.012) | 0.428 | 0.720 |
| p-tau181 | cingulate gyrus part of cingulum (r) ISOVF | -0.008 (-0.032, 0.017) | 0.528 | 0.778 |
| p-tau181 | cingulate gyrus part of cingulum (l) ISOVF | -0.005 (-0.027, 0.016) | 0.625 | 0.819 |
| p-tau181 | anterior thalamic radiation (r) ISOVF | 0.004 (-0.016, 0.023) | 0.714 | 0.849 |
| p-tau181 | anterior thalamic radiation (l) ISOVF | 0.004 (-0.018, 0.025) | 0.750 | 0.849 |
| p-tau181 | acoustic radiation (r) ISOVF | 0.004 (-0.021, 0.029) | 0.759 | 0.849 |
| p-tau181 | acoustic radiation (l) ISOVF | 0.003 (-0.017, 0.023) | 0.770 | 0.849 |
| Aβ42/40 | uncinate fasciculus (r) OD | 0.033 (0.012, 0.054) | 0.003 | 0.034 |
| Aβ42/40 | uncinate fasciculus (l) OD | 0.027 (0.006, 0.049) | 0.012 | 0.068 |
| Aβ42/40 | superior thalamic radiation (r) OD | 0.025 (0.004, 0.047) | 0.022 | 0.090 |
| Aβ42/40 | superior thalamic radiation (l) OD | 0.016 (0.002, 0.030) | 0.026 | 0.098 |
| Aβ42/40 | superior longitudinal fasciculus (r) OD | 0.022 (0.002, 0.041) | 0.028 | 0.099 |
| Aβ42/40 | superior longitudinal fasciculus (l) OD | -0.007 (-0.015, 0.001) | 0.077 | 0.208 |
| Aβ42/40 | posterior thalamic radiation (r) OD | -0.011 (-0.026, 0.003) | 0.115 | 0.255 |
| Aβ42/40 | posterior thalamic radiation (l) OD | -0.012 (-0.029, 0.004) | 0.145 | 0.310 |
| Aβ42/40 | parahippocampal part of cingulum (r) OD | 0.010 (-0.004, 0.024) | 0.147 | 0.310 |
| Aβ42/40 | parahippocampal part of cingulum (l) OD | -0.009 (-0.021, 0.004) | 0.174 | 0.356 |
| Aβ42/40 | middle cerebellar peduncle (l) OD | -0.008 (-0.020, 0.004) | 0.190 | 0.383 |
| Aβ42/40 | medial lemniscus (r) OD | 0.012 (-0.007, 0.031) | 0.205 | 0.395 |
| Aβ42/40 | medial lemniscus (l) OD | 0.013 (-0.008, 0.034) | 0.233 | 0.425 |
| Aβ42/40 | inferior longitudinal fasciculus (r) OD | -0.007 (-0.019, 0.005) | 0.253 | 0.425 |
| Aβ42/40 | inferior longitudinal fasciculus (l) OD | -0.010 (-0.029, 0.008) | 0.280 | 0.445 |
| Aβ42/40 | inferior fronto-occipital fasciculus (r) OD | 0.008 (-0.013, 0.028) | 0.471 | 0.650 |
| Aβ42/40 | inferior fronto-occipital fasciculus (l) OD | -0.006 (-0.024, 0.011) | 0.477 | 0.650 |
| Aβ42/40 | forceps major (r) OD | 0.007 (-0.012, 0.026) | 0.487 | 0.652 |
| Aβ42/40 | forceps major (l) OD | -0.003 (-0.011, 0.006) | 0.519 | 0.680 |
| Aβ42/40 | corticospinal tract (r) OD | 0.004 (-0.009, 0.018) | 0.530 | 0.681 |
| Aβ42/40 | corticospinal tract (l) OD | -0.004 (-0.020, 0.012) | 0.595 | 0.736 |
| Aβ42/40 | cingulate gyrus part of cingulum (r) OD | 0.006 (-0.015, 0.026) | 0.600 | 0.736 |
| Aβ42/40 | cingulate gyrus part of cingulum (l) OD | -0.002 (-0.017, 0.014) | 0.821 | 0.901 |
| Aβ42/40 | anterior thalamic radiation (r) OD | 0.002 (-0.014, 0.017) | 0.845 | 0.905 |
| Aβ42/40 | anterior thalamic radiation (l) OD | 0.001 (-0.016, 0.019) | 0.884 | 0.928 |
| Aβ42/40 | acoustic radiation (r) OD | -0.001 (-0.019, 0.016) | 0.887 | 0.928 |
| Aβ42/40 | acoustic radiation (l) OD | 0.001 (-0.017, 0.019) | 0.909 | 0.937 |
| GFAP | uncinate fasciculus (r) OD | -0.038 (-0.056, -0.019) | 0.000 | 0.000 |
| GFAP | uncinate fasciculus (l) OD | -0.025 (-0.045, -0.006) | 0.011 | 0.068 |
| GFAP | superior thalamic radiation (r) OD | -0.023 (-0.042, -0.004) | 0.016 | 0.092 |
| GFAP | superior thalamic radiation (l) OD | -0.016 (-0.031, -0.002) | 0.022 | 0.102 |
| GFAP | superior longitudinal fasciculus (r) OD | -0.024 (-0.046, -0.003) | 0.028 | 0.122 |
| GFAP | superior longitudinal fasciculus (l) OD | -0.018 (-0.036, 0.000) | 0.050 | 0.173 |
| GFAP | posterior thalamic radiation (r) OD | 0.008 (-0.001, 0.016) | 0.085 | 0.239 |
| GFAP | posterior thalamic radiation (l) OD | -0.012 (-0.028, 0.004) | 0.133 | 0.326 |
| GFAP | parahippocampal part of cingulum (r) OD | -0.013 (-0.030, 0.005) | 0.157 | 0.378 |
| GFAP | parahippocampal part of cingulum (l) OD | -0.014 (-0.036, 0.008) | 0.206 | 0.428 |
| GFAP | middle cerebellar peduncle (l) OD | -0.010 (-0.026, 0.006) | 0.221 | 0.442 |
| GFAP | medial lemniscus (r) OD | 0.005 (-0.003, 0.013) | 0.247 | 0.463 |
| GFAP | medial lemniscus (l) OD | -0.012 (-0.033, 0.010) | 0.283 | 0.493 |
| GFAP | inferior longitudinal fasciculus (r) OD | -0.010 (-0.029, 0.009) | 0.284 | 0.493 |
| GFAP | inferior longitudinal fasciculus (l) OD | -0.011 (-0.032, 0.010) | 0.300 | 0.500 |
| GFAP | inferior fronto-occipital fasciculus (r) OD | -0.010 (-0.031, 0.011) | 0.345 | 0.548 |
| GFAP | inferior fronto-occipital fasciculus (l) OD | 0.005 (-0.006, 0.017) | 0.371 | 0.559 |
| GFAP | forceps major (r) OD | 0.008 (-0.010, 0.026) | 0.386 | 0.560 |
| GFAP | forceps major (l) OD | 0.005 (-0.007, 0.018) | 0.407 | 0.578 |
| GFAP | corticospinal tract (r) OD | -0.004 (-0.018, 0.009) | 0.529 | 0.655 |
| GFAP | corticospinal tract (l) OD | -0.003 (-0.017, 0.011) | 0.674 | 0.810 |
| GFAP | cingulate gyrus part of cingulum (r) OD | 0.004 (-0.014, 0.021) | 0.686 | 0.812 |
| GFAP | cingulate gyrus part of cingulum (l) OD | 0.004 (-0.017, 0.025) | 0.710 | 0.819 |
| GFAP | anterior thalamic radiation (r) OD | 0.002 (-0.010, 0.014) | 0.742 | 0.828 |
| GFAP | anterior thalamic radiation (l) OD | -0.003 (-0.019, 0.014) | 0.763 | 0.844 |
| GFAP | acoustic radiation (r) OD | -0.001 (-0.017, 0.014) | 0.851 | 0.891 |
| GFAP | acoustic radiation (l) OD | 0.000 (-0.014, 0.014) | 0.962 | 0.969 |
| NfL | uncinate fasciculus (r) OD | -0.023 (-0.039, -0.007) | 0.005 | 0.081 |
| NfL | uncinate fasciculus (l) OD | -0.017 (-0.033, -0.001) | 0.033 | 0.203 |
| NfL | superior thalamic radiation (r) OD | -0.011 (-0.023, 0.001) | 0.086 | 0.296 |
| NfL | superior thalamic radiation (l) OD | -0.011 (-0.027, 0.006) | 0.210 | 0.531 |
| NfL | superior longitudinal fasciculus (r) OD | -0.007 (-0.019, 0.005) | 0.250 | 0.560 |
| NfL | superior longitudinal fasciculus (l) OD | -0.009 (-0.024, 0.006) | 0.258 | 0.562 |
| NfL | posterior thalamic radiation (r) OD | -0.007 (-0.018, 0.005) | 0.271 | 0.565 |
| NfL | posterior thalamic radiation (l) OD | -0.010 (-0.029, 0.008) | 0.272 | 0.565 |
| NfL | parahippocampal part of cingulum (r) OD | -0.004 (-0.011, 0.003) | 0.307 | 0.573 |
| NfL | parahippocampal part of cingulum (l) OD | -0.009 (-0.027, 0.009) | 0.312 | 0.573 |
| NfL | middle cerebellar peduncle (l) OD | 0.008 (-0.008, 0.023) | 0.314 | 0.573 |
| NfL | medial lemniscus (r) OD | -0.009 (-0.027, 0.009) | 0.327 | 0.574 |
| NfL | medial lemniscus (l) OD | 0.009 (-0.009, 0.027) | 0.331 | 0.574 |
| NfL | inferior longitudinal fasciculus (r) OD | -0.007 (-0.022, 0.008) | 0.343 | 0.574 |
| NfL | inferior longitudinal fasciculus (l) OD | -0.006 (-0.020, 0.007) | 0.348 | 0.574 |
| NfL | inferior fronto-occipital fasciculus (r) OD | -0.006 (-0.021, 0.009) | 0.444 | 0.656 |
| NfL | inferior fronto-occipital fasciculus (l) OD | -0.004 (-0.014, 0.007) | 0.475 | 0.675 |
| NfL | forceps major (r) OD | -0.005 (-0.018, 0.009) | 0.490 | 0.689 |
| NfL | forceps major (l) OD | -0.005 (-0.019, 0.009) | 0.507 | 0.701 |
| NfL | corticospinal tract (r) OD | 0.003 (-0.007, 0.013) | 0.519 | 0.701 |
| NfL | corticospinal tract (l) OD | -0.004 (-0.021, 0.012) | 0.614 | 0.768 |
| NfL | cingulate gyrus part of cingulum (r) OD | -0.004 (-0.023, 0.014) | 0.649 | 0.788 |
| NfL | cingulate gyrus part of cingulum (l) OD | 0.002 (-0.009, 0.012) | 0.751 | 0.859 |
| NfL | anterior thalamic radiation (r) OD | -0.001 (-0.008, 0.006) | 0.790 | 0.878 |
| NfL | anterior thalamic radiation (l) OD | -0.001 (-0.013, 0.010) | 0.818 | 0.885 |
| NfL | acoustic radiation (r) OD | 0.001 (-0.012, 0.015) | 0.851 | 0.905 |
| NfL | acoustic radiation (l) OD | 0.001 (-0.017, 0.019) | 0.930 | 0.944 |
| p-tau181 | uncinate fasciculus (r) OD | 0.015 (0.001, 0.028) | 0.037 | 0.558 |
| p-tau181 | uncinate fasciculus (l) OD | 0.016 (-0.002, 0.033) | 0.074 | 0.558 |
| p-tau181 | superior thalamic radiation (r) OD | -0.011 (-0.025, 0.003) | 0.111 | 0.558 |
| p-tau181 | superior thalamic radiation (l) OD | 0.016 (-0.004, 0.037) | 0.116 | 0.558 |
| p-tau181 | superior longitudinal fasciculus (r) OD | 0.015 (-0.006, 0.036) | 0.166 | 0.558 |
| p-tau181 | superior longitudinal fasciculus (l) OD | 0.009 (-0.005, 0.023) | 0.205 | 0.561 |
| p-tau181 | posterior thalamic radiation (r) OD | 0.009 (-0.005, 0.022) | 0.209 | 0.561 |
| p-tau181 | posterior thalamic radiation (l) OD | 0.011 (-0.008, 0.029) | 0.257 | 0.603 |
| p-tau181 | parahippocampal part of cingulum (r) OD | -0.009 (-0.027, 0.008) | 0.297 | 0.647 |
| p-tau181 | parahippocampal part of cingulum (l) OD | -0.007 (-0.023, 0.009) | 0.387 | 0.713 |
| p-tau181 | middle cerebellar peduncle (l) OD | 0.006 (-0.010, 0.022) | 0.485 | 0.775 |
| p-tau181 | medial lemniscus (r) OD | 0.007 (-0.014, 0.028) | 0.505 | 0.775 |
| p-tau181 | medial lemniscus (l) OD | 0.003 (-0.005, 0.011) | 0.505 | 0.775 |
| p-tau181 | inferior longitudinal fasciculus (r) OD | -0.006 (-0.023, 0.012) | 0.530 | 0.778 |
| p-tau181 | inferior longitudinal fasciculus (l) OD | 0.005 (-0.013, 0.023) | 0.568 | 0.807 |
| p-tau181 | inferior fronto-occipital fasciculus (r) OD | -0.005 (-0.026, 0.015) | 0.614 | 0.819 |
| p-tau181 | inferior fronto-occipital fasciculus (l) OD | 0.003 (-0.009, 0.015) | 0.654 | 0.825 |
| p-tau181 | forceps major (r) OD | -0.002 (-0.010, 0.006) | 0.654 | 0.825 |
| p-tau181 | forceps major (l) OD | -0.003 (-0.018, 0.013) | 0.731 | 0.849 |
| p-tau181 | corticospinal tract (r) OD | 0.002 (-0.013, 0.018) | 0.755 | 0.849 |
| p-tau181 | corticospinal tract (l) OD | 0.003 (-0.018, 0.024) | 0.757 | 0.849 |
| p-tau181 | cingulate gyrus part of cingulum (r) OD | 0.003 (-0.018, 0.024) | 0.771 | 0.849 |
| p-tau181 | cingulate gyrus part of cingulum (l) OD | -0.002 (-0.021, 0.017) | 0.826 | 0.871 |
| p-tau181 | anterior thalamic radiation (r) OD | -0.002 (-0.020, 0.017) | 0.857 | 0.890 |
| p-tau181 | anterior thalamic radiation (l) OD | 0.001 (-0.011, 0.012) | 0.896 | 0.908 |
| p-tau181 | acoustic radiation (r) OD | -0.001 (-0.020, 0.018) | 0.901 | 0.908 |
| p-tau181 | acoustic radiation (l) OD | 0.000 (-0.012, 0.012) | 0.970 | 0.970 |

Abbreviations: Aβ, amyloid-β; GFAP, glial fibrillary acidic protein; NfL, neurofilament light chain; p-tau181, tau phosphorylated at threonine 181; CI, confidence interval; FA, fractional anisotropy; MD, mean diffusivity; ICVF, intracellular volume fraction; ISOVF, isotropic volume fraction; OD, orientation dispersion; r, right; l, left; FDR, false discovery rates.

Model was adjusted for sex, age, ethnicity, townsend deprivation index, assessment center, smoking status, alcohol use, family history of dementia, *APOE* ε4 allele, hypertension, diabetes, cardiovascular arterial disease, history of COVID-19 infection, and total intracranial volume.

# Supplementary Table 16. Interactive associations of educational attainment and elevated plasma AD-related biomarkers on cognitive decline.

| **Plasma biomarkers** | **Cognitive function** | **β (95% CI)** | ***P-*value** |
| --- | --- | --- | --- |
| GFAP × education × time | Reaction time | 0.002 (-0.050, 0.053) | 0.954 |
| GFAP × education × time | Symbol digit substitution | 0.075 (0.025, 0.125) | **0.003** |
| GFAP × education × time | Trail making | -0.020 (-0.077, 0.038) | 0.506 |
| GFAP × education × time | Pairs matching | 0.008 (-0.064, 0.081) | 0.821 |
| GFAP × education × time | Paired associate learning | 0.035 (-0.018, 0.089) | 0.196 |
| GFAP × education × time | Fluid intelligence | 0.036 (-0.008, 0.080) | 0.111 |
| NfL × education × time | Reaction time | -0.010 (-0.062, 0.041) | 0.691 |
| NfL × education × time | Symbol digit substitution | 0.060 (0.010, 0.110) | **0.020** |
| NfL × education × time | Trail making | -0.013 (-0.071, 0.044) | 0.656 |
| NfL × education × time | Pairs matching | 0.028 (-0.044, 0.101) | 0.440 |
| NfL × education × time | Paired associate learning | 0.052 (-0.002, 0.105) | 0.060 |
| NfL × education × time | Fluid intelligence | 0.021 (-0.024, 0.065) | 0.359 |
| p-tau181 × education × time | Reaction time | -0.044 (-0.094, 0.006) | 0.087 |
| p-tau181 × education × time | Symbol digit substitution | -0.006 (-0.054, 0.042) | 0.807 |
| p-tau181 × education × time | Trail making | -0.035 (-0.090, 0.021) | 0.218 |
| p-tau181 × education × time | Pairs matching | 0.028 (-0.042, 0.098) | 0.427 |
| p-tau181 × education × time | Paired associate learning | 0.001 (-0.051, 0.053) | 0.978 |
| p-tau181 × education × time | Fluid intelligence | -0.005 (-0.048, 0.037) | 0.812 |
| Aβ42/40 × education × time | Reaction time | 0.016 (-0.033, 0.065) | 0.524 |
| Aβ42/40 × education × time | Symbol digit substitution | -0.015 (-0.063, 0.033) | 0.535 |
| Aβ42/40 × education × time | Trail making | 0.004 (-0.051, 0.059) | 0.894 |
| Aβ42/40 × education × time | Pairs matching | 0.010 (-0.059, 0.078) | 0.785 |
| Aβ42/40 × education × time | Paired associate learning | 0.014 (-0.037, 0.065) | 0.603 |
| Aβ42/40 × education × time | Fluid intelligence | -0.017 (-0.059, 0.026) | 0.441 |

Abbreviations: Aβ, amyloid-β; GFAP, glial fibrillary acidic protein; NfL, neurofilament light chain; p-tau181, tau phosphorylated at threonine 181; CI, confidence interval.

Model was adjusted for sex, age, ethnicity, townsend deprivation index, assessment center, smoking status, alcohol use, *APOE* ε4 allele, hypertension, diabetes, cardiovascular arterial disease, and history of COVID-19 infection.

**
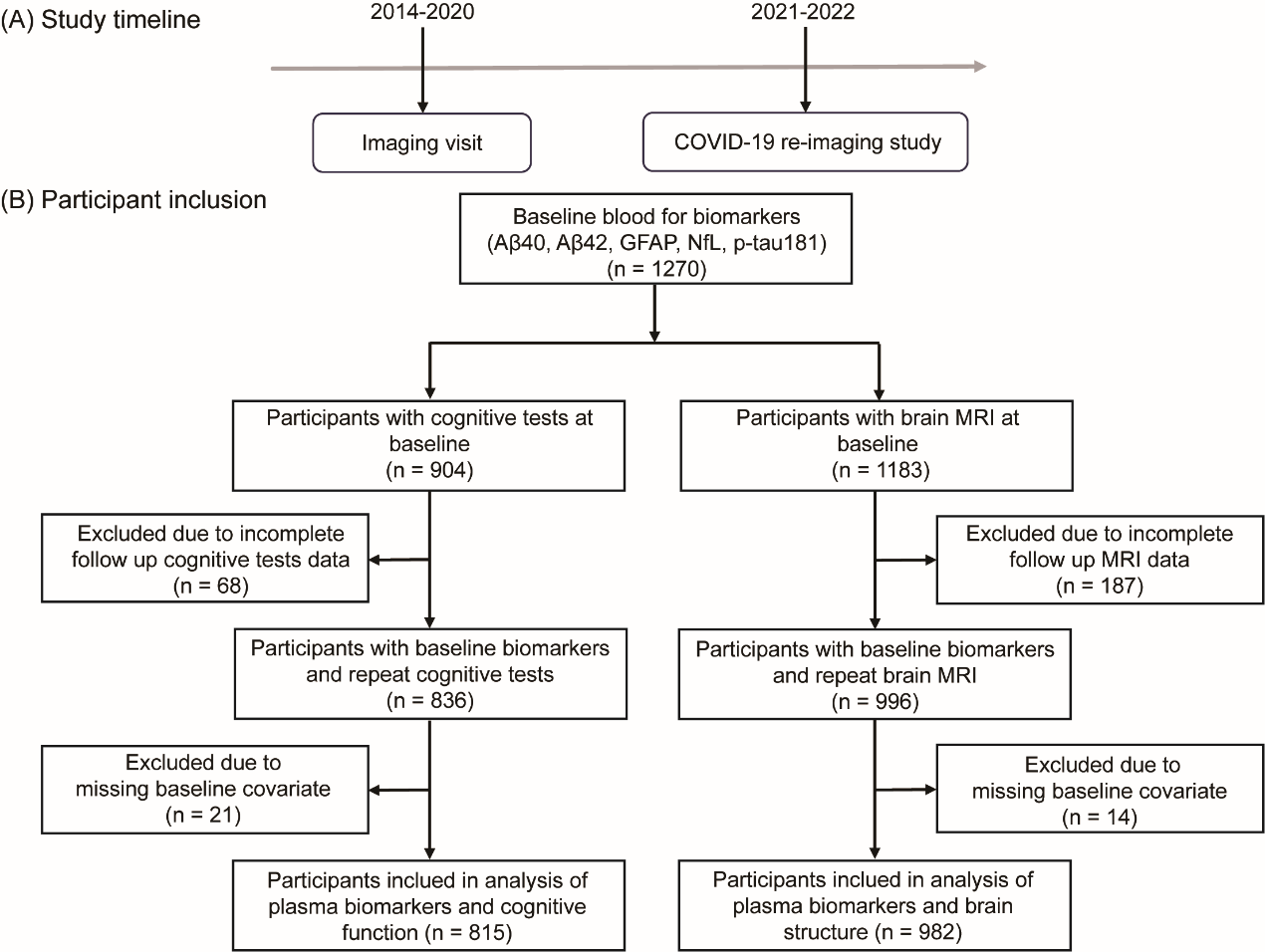
**

# Supplementary Figure 1. Study timeline (A) and flowchart (B) of study participants.

Abbreviations: Aβ, amyloid-β; GFAP, glial fibrillary acidic protein; NfL, neurofilament light chain; p-tau181, tau phosphorylated at threonine 181; MRI, magnetic resonance imaging.
